# T cells in health and disease

**DOI:** 10.1038/s41392-023-01471-y

**Published:** 2023-06-19

**Authors:** Lina Sun, Yanhong Su, Anjun Jiao, Xin Wang, Baojun Zhang

**Affiliations:** 1 Department of Pathogenic Microbiology and Immunology, School of Basic Medical Sciences, Xi’an Jiaotong University, Xi’an, Shaanxi 710061 China; 2 Institute of Infection and Immunity, Translational Medicine Institute, Xi’an Jiaotong University Health Science Center, Xi’an, Shaanxi 710061 China; 3 Key Laboratory of Environment and Genes Related to Diseases, Ministry of Education, Xi’an, Shaanxi 710061 China; 4 Xi’an Key Laboratory of Immune Related Diseases, Xi’an, Shannxi 710061 China

**Keywords:** Adaptive immunity, Tumour immunology, Infectious diseases, Immunological disorders

## Abstract

T cells are crucial for immune functions to maintain health and prevent disease. T cell development occurs in a stepwise process in the thymus and mainly generates CD4^+^ and CD8^+^ T cell subsets. Upon antigen stimulation, naïve T cells differentiate into CD4^+^ helper and CD8^+^ cytotoxic effector and memory cells, mediating direct killing, diverse immune regulatory function, and long-term protection. In response to acute and chronic infections and tumors, T cells adopt distinct differentiation trajectories and develop into a range of heterogeneous populations with various phenotype, differentiation potential, and functionality under precise and elaborate regulations of transcriptional and epigenetic programs. Abnormal T-cell immunity can initiate and promote the pathogenesis of autoimmune diseases. In this review, we summarize the current understanding of T cell development, CD4^+^ and CD8^+^ T cell classification, and differentiation in physiological settings. We further elaborate the heterogeneity, differentiation, functionality, and regulation network of CD4^+^ and CD8^+^ T cells in infectious disease, chronic infection and tumor, and autoimmune disease, highlighting the exhausted CD8^+^ T cell differentiation trajectory, CD4^+^ T cell helper function, T cell contributions to immunotherapy and autoimmune pathogenesis. We also discuss the development and function of γδ T cells in tissue surveillance, infection, and tumor immunity. Finally, we summarized current T-cell-based immunotherapies in both cancer and autoimmune diseases, with an emphasis on their clinical applications. A better understanding of T cell immunity provides insight into developing novel prophylactic and therapeutic strategies in human diseases.

## Introduction

T lymphocytes (T cells) are the major cell components of the adaptive immune system, responsible for mediating cell-based immune responses to keep the host healthy and prevent various types of diseases. T cells are developed from bone marrow (BM)-derived thymocyte progenitors in the thymus, and broadly grouped into CD4^+^ and CD8^+^ αβ T cells in addition to rear populations of γδ T cells and natural killer T (NKT) cells. αβ T cells recognize antigens that are presented by major histocompatibility complex (MHC) molecules on antigen-presenting cells (APCs). Upon recognition of cognate antigens (signals 1) by T cell receptor (TCR) and costimulatory molecules (signals 2) on APCs, and cytokines (signals 3), naïve CD4^+^ and CD8^+^ T cells undergo activation, clonal expansion, and differentiation to execute their effector functions of killing infected cells, producing cytokines and regulating immune responses. A small population of T cells develops into memory T cells which exhibit rapid effector functions upon reencountering the same antigens and provide the host with potent and long-term protection. In parallel, there exists a subpopulation of CD4^+^ T cells, named regulatory T (T_reg_) cells, that maintain peripheral immune tolerance. Over the past few decades, our knowledge of T cells regarding their classification, differentiation, cellular and molecular regulatory mechanisms, particularly phenotypes and functions in healthy conditions and immune-related diseases, has expanded significantly. Hence, novel strategies engaging T cell functions have been extensively developed and demonstrated unprecedented clinical efficacy in the past few decades.

In this review, we comprehensively summarize the current understandings of T cell biology and functions in both physiological and pathological settings, including the following points: (1) describe the T cell development regarding their differentiation process, T cell lineage commitment, β-selection, and CD4/CD8 lineage choice; (2) introduce major CD4^+^ and CD8^+^ T cell classification, differentiation, and the underlying regulatory mechanisms; (3) further discuss how CD8^+^ and CD4^+^ T cells respond, differentiate and contribute in infectious diseases, chronic infections and tumors, and autoimmune diseases; (4) γδ T cell development, effector subsets and function in tissue surveillance, infection, and tumor immunity; (5) T cell-based immunotherapies in cancer and autoimmune diseases and their clinical applications. Specifically, we highlight the cell signature, differentiation trajectory, regulatory mechanisms, and contributions to anti-tumor immunity of exhausted CD8^+^ T cells, as well as the roles of CD4^+^ T cells in helping CD8^+^ T cell responses.

## T cell development

**Fig. 1:**
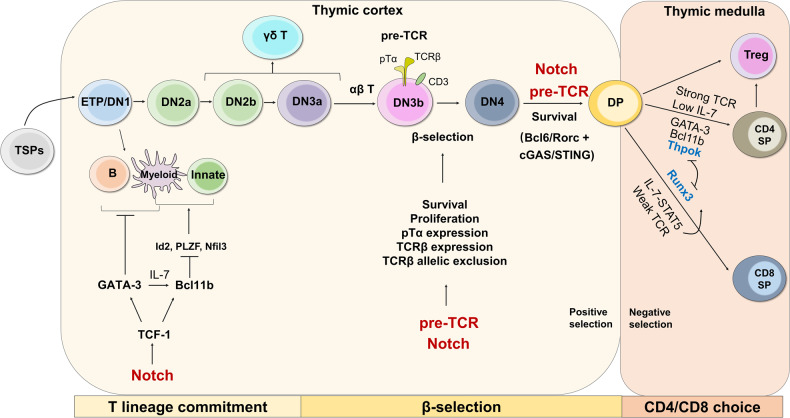
Overview of thymocyte development and regulatory mechanism. T cell development experiences three key steps: T cell lineage commitment, β-selection, and CD4/CD8 lineage choice, where T cells undergo sequential developmental stages from TSPs to DN, DP, and SP. ETPs (DN1) possess the potential to differentiate into B cells, myeloid cells, and innate-type of T cells, while DN3 can differentiate into γδ T cells. Induced by Notch signaling, transcription factors TCF-1, GATA-3, and Bcl11b play critical roles in promoting T cell lineage commitment by limiting other lineage differentiation. A pre-TCR complex consisting of TCRβ, pTα, and CD3 molecules on DN3 enforces β-selection and DN3 to DN4 development. Both pre-TCR and Notch signaling play critical roles in driving β-selection and DN to DP transition. Following positive and negative selection in the thymic cortex and medulla, respectively, DP cells differentiate into either CD4^+^ SP under the regulation of strong TCR and Thpok or CD8^+^ SP under the regulation of weak TCR and Runx3

T cell development begins with BM-derived thymic seeding progenitors (TSPs) in the thymus, where T cells undergo a series of developmental stages including double negative (CD4^−^CD8^−^, DN), double positive (CD4^+^CD8^+^, DP), and single positive (CD4^−^CD8^+^ or CD4^+^CD8^−^, SP)^1–3^ (Fig. 1). DN thymocytes can be divided into four distinct stages from DN1 to DN4 based on CD44 and CD25 expression among lineage negative population.^2,4–6^ Upon Notch signaling, ETPs (DN1) acquire CD25 expression and progress into the DN2a stage, which launches the T cell lineage commitment.^4,5^ Bifurcation of αβ and γδ T cell lineage occurs at DN2b and DN3a stage along with upregulation of genes associated with TCRγ, TCRδ, and TCRβ rearrangement.^7^ A functional pre-TCR complex, consisting of CD3 protein, TCRβ and invariant pre-TCRα (pTα), drives DN3 cells to DN4, CD4^+^CD8^−^ immature single positive (ISP), and DP cell development.^7^ Those expressing a TCRβ chain can initiate TCRα rearrangement and then form a fully functional αβTCR on the surface, which recognizes MHC I- or MHC II-peptide complexes presented by thymic APCs to become either CD8^+^ SP or CD4^+^ SP thymocytes.^8^ On one hand, the interaction of peptide-MHC with moderate affinity rescues DP thymocytes from apoptosis (known as positive selection) in the thymic cortex and progresses into the SP stage.^8^ On the other hand, recognition of self-peptide triggers immense death (known as negative selection) or skews CD4^+^ T cells towards T_reg_ cells in the thymic medulla.^9^ The following three steps and relevant signals are required for T cell fate decision and development.

### Orchestrated trajectory for T cell lineage commitment

ETPs still possess the potential to differentiate into other immune cell lineages, such as B cells, NK cells, dendritic and myeloid cells.^10,11^ How ETPs commit to T cell lineage and lose the ability to convert to alternative lineages? It is well-appreciated that Notch signaling is essential for the initial commitment of T cell lineage in the thymus.^12,13^ Notch1 signaling induces the expression of transcription factor (TF) T cell factor 1 (TCF-1, encoded by *Tcf7*), which is required for the generation, survival, and proliferation of ETPs.^14–16^ TCF-1 promotes the upregulation of T cell-specific TFs GATA-3 and Bcl11b,^15,16^ and GATA-3 as well as IL-7/IL-7R signal are required for Bcl11b activation.^17–19^ GATA-3 suppresses both B cell and myeloid cell differentiation in TCF-1-deficient ETPs,^15^ whereas Bcl11b restricts the progenitor differentiation into innate lymphoid and myeloid lineages.^20–22^ Mechanistically, Bcl11b blocks expression of Id2, PLZF, and Nfil3 expression,^21,23,24^ in which Id2-repressed E protein E2A is critical for innate lymphoid cells including NK cell development,^25–27^ while PLZF and Nfil3 promote innate-type T cell development.^28–30^ Hence, enforced expression of Bcl11b can restore the DN1 to DN2 transition block resulted from TCF-1 deficiency.^15^ Future research needs to clarify whether GATA-3 facilitates T cell lineage and limits other lineages independent of Bcl11b. Taken together, following T cell lineage specification, the committed DN2b cells completely step on the T cell development journey.^31^

### DN-DP transition driven by β-selection

Following the accomplishment of TCRβ rearrangement, DN3 cells expressing pre-TCR assembled with the TCRβ chain together with pTα and CD3 molecules (known as β-selection) differentiate into αβ T cells, otherwise, skew into γδ T cells.^7,32,33^ To date, two major signals are involved in the β-selection process: pre-TCR and Notch signaling. The pre-TCR signaling prevents thymocytes from apoptosis, stimulates their proliferation, induces allelic exclusion at the TCRβ locus in DN3b cells post-β-selection and promotes DN to DP transition.^34–37^ However, pre-TCR signaling alone is not sufficient for thymocyte development, as isolated DN3 thymocytes fail to differentiate into DP cells in the absence of a stromal cell-derived Notch signal.^38–40^ Notch signaling has been shown to promote T lineage commitment,^41^ thymocyte survival,^42^ DN to DP stage transition,^42^ and expression of pre-TCR components.^43,44^ Recently, Notch-induced endoplasmic reticulum (ER)-associated degradation (ERAD) mediates proteasomal degradation of misfolded proteins, which becomes a prerequisite for thymocyte β-selection.^45^ Pre-TCR and Notch signaling, by targeting ubiquitin ligase subunits Fbxl1 and Fbxl12, respectively, promote the cell cycle progression of β-selected thymocytes via accelerating degradation of cyclin-dependent kinase inhibitor Cdkn1b.^46^ Furthermore, β-selected thymocytes form an immunological synapse to promote proliferation, which relies on the cooperation between Notch and pre-TCR signaling.^47^ Interestingly, pre-TCR independent mechanisms also regulate thymocyte development. Recent studies from our and other groups demonstrated that zinc finger protein Zfp335 controlled thymocyte survival and DN to DP transition by inducing Bcl-6/Rorc expression or cGAS/STING suppression in a pre-TCR independent manner.^48,49^

### Choice to become CD4^+^ or CD8^+^ T cells

Following positive selection, DP cells bearing MHC class I- or MHC class II-TCRs differentiate into either CD8^+^ or CD4^+^ T cells, termed as CD4/CD8 lineage choice.^50,51^ A well-known theory holds that DP thymocytes received positive selection signals initially terminate CD8 gene transcription and become CD4^+^CD8^lo^ intermediate cells which further progress into CD4^+^ or CD8^+^ T cells depending on TCR signaling or cytokines stimulation.^52–54^ Persistent and strong TCR signals in intermediate thymocytes trigger differentiation into CD4^+^CD8^-^ SP cells largely by inhibiting IL-7-mediated signaling, whereas transient and weak TCR signals force these cells into CD4^-^CD8^+^ SP cells, which relies on signals from IL-7 and other common gamma chain (γc) cytokines.^55–57^

Thpok and Runx3 are two antagonistic TFs controlling the lineage choice between CD4^+^ or CD8^+^ T cells. Thpok is highly expressed in CD4^+^ but not CD8^+^ thymocytes, and serves as a master regulator for CD4 lineage commitment.^58,59^ Mice with Thpok depletion or a missense mutation lack CD4^+^ T cells,^58,60–63^ whereas ectopic expression of Thpok strongly drives DP thymocytes into CD4^+^ SP cells.^58,59^ Mechanistically, Thpok represses Runx3 and CD8 lineage-related genes.^61,64,65^ In contrast, Runx3 facilitates CD8^+^ T cell development by directly downregulating CD4 and Thpok expression.^62,66^ In addition, Bcl11b promotes CD4 lineage commitment by directly targeting to several Thpok locus^67,68^ and Runx3 promoter region.^67^ TCR signaling-induced GATA-3 is also required for CD4 lineage commitment by enhancing Thpok expression,^69,70^ while the IL-7-STAT5 axis acts upstream of Runx3 to enhance its expression and promote CD8^+^ T cell development.^71^ Therefore, the balance between Thpok and Runx3 decides the lineage choice of CD4^+^ versus CD8^+^ T cells.

## CD4^+^ T cell classification and differentaiton

**Fig. 2:**
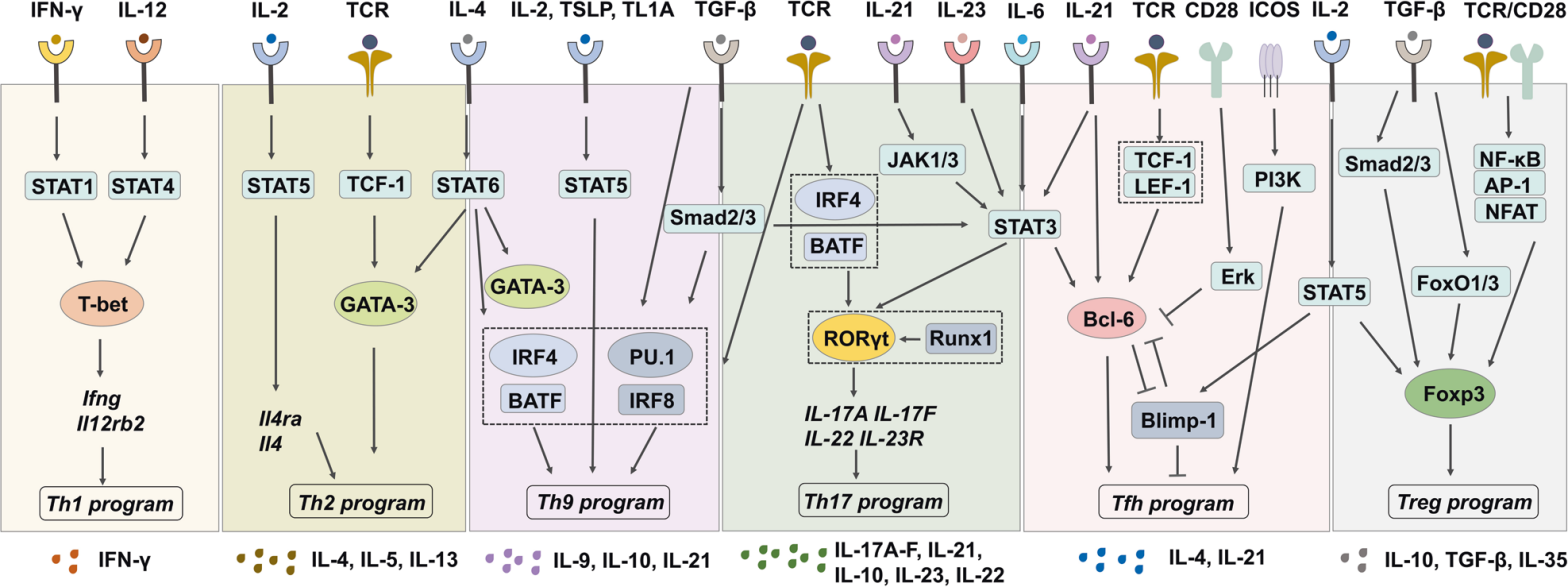
Cytokine signalings regulate CD4^+^ Th cell differentiation. Upon TCR stimulation, naïve CD4^+^ T cells can be differentiated into distinct effector Th subsets under different cytokines and costimulatory stimulation. IFN-γ and IL-12 drive Th1 cell differentiation by inducing the master TF T-bet expression through STAT1 and STAT4, respectively. Th2 cells are induced by TCR-stimulated TCF-1 activation and cytokine IL-2 and IL-4 signaling, expressing key TF GATA-3. Th9 cells are induced under TCR stimulation in the presence of IL-4 and TGF-β, and enhanced development by STAT5 activation. While IL-6 and TGF-β drive Th17 cell differentiation, IL-21 and IL-23 stabilize Th17 lineage by inducing RORγt. Cytokines IL-6 and IL-21 promote, while IL-2 inhibits Tfh cell differentiation. Costimulatory signaling from CD28 and ICOS play opposite roles in Tfh cell development. T_reg_ cells can be differentiated upon TCR/CD28 stimulation in the presence of TGF-β and IL-2 through inducing Foxp3 expression. Shared cytokines are illustrated between cells: IL-4 for Th2 and Th9, TGF-β for Th9 and Th17, IL-6 for Th17 and Tfh, and IL-2 for Tfh and T_reg_ cells. The same cytokines may induce different downstream signaling cascade and differentiation fate. For instance, IL-6-induced STAT3 activation leads to the expression of RORγt in Th17 cells but Bcl-6 in Tfh cells. Signaling complexes formed are indicated in the dashed squares

CD4^+^ T helper (Th) cells are a heterogeneous group of T cells playing central roles in almost all aspects of immune responses. CD4^+^ T cells can be activated by peptide-MHC class II complex on APCs, costimulatory stimulation, and cytokine signaling^72–74^ and differentiate into several subsets with a distinct expression of surface molecules, cytokines, and key TFs,^75,76^ such as Th1, Th2, T_reg_, follicular helper T (Tfh), Th17, Th9, Th22, and CD4^+^ cytotoxic T lymphocytes (CTLs), etc.^77^ Here, we will introduce six major Th subsets and the regulatory pathways of their differentiation (Fig. 2).

Th1 cells are the major participants in protecting hosts against intracellular bacteria and viruses by producing the pro-inflammatory cytokine IFN-γ. IL-12 and IFN-γ are two cytokines essential for Th1 differentiation.^78^ TCR stimulation and IFN-γ-STAT1 signaling induce the expression of T-bet (encoded by *Tbx21*), the major TF driving Th1 differentiation while suppressing Th2/Th17 lineages.^79,80^ T-bet can directly bind to the *Ifng* gene to increase the expression of IFN-γ^80,81^ and meanwhile promote the expression of IL-12Rβ2, conferring IL-12 responsiveness.^82^ IL-12 signaling via STAT4 activation, in turn, maintains T-bet expression.^83^ These feedback loops all contribute to Th1 differentiation.

Th2 cells, defined by expression of TF GATA-3 and cytokines IL-4, IL-5, and IL-13, protect the host against helminth infections, facilitate tissue repair, as well as contribute to chronic inflammation such as asthma and allergy.^84^ IL-4 secreted by dendritic cells (DCs) and innate lymphoid cell group 2 (ILC2) binds to IL-4R on CD4^+^ T cells, leading to the expression of GATA-3 through STAT6 phosphorylation and subsequent production of Th2-related cytokines.^85^ Autocrine production of IL-4 by activated CD4^+^ T cells further promotes Th2 differentiation.^86^ In addtion, GATA-3 mediates the repression of Th1 cell development by sliencing Th1-related genes such as *Tbx21*, *Ifng*, *Stat4*, and *Il12rb2*.^87^ STAT5 signaling primed by IL-2 is required for maintaining the expression of *Il4ra* and increasing the accessibility of *Il4* chromatin.^87,88^ Other TFs such as NFAT1, c-Maf, IRF4, and JunB can promote Th2 program by inducing IL-4 production.^87^ In addition, TCF-1, activated by TCR stimulation, has been found to initiate Th2 cell differentiation by promoting GATA-3 expression.^89^

Th9 cells are a newly identified subset of CD4^+^ T cells, playing critical roles in infectious diseases, allergy, cancer, and autoimmune immunity.^90–94^ Th9 cells can be induced in vitro by TCR stimulation in the presence of IL-4 and TGF-β, and are characterized by expressing high levels of IL-9 and prominent TFs IRF4 and PU.1.^90,95–97^ Besides IL-9, IL-10, and IL-21 are also produced by Th9 cells.^98^ STAT6 phosphorylation mediated by IL-4 signaling induces expression of GATA-3, IRF4 and BATF to promote *IL-9* transcription and Th9 cell development.^99,100^ Besides, TGF-β signaling activates Smads (Smad2/3), PU.1 and IRF8, contributing to Th9 cell differentiation.^99,100^ Furthermore, IRF4, PU.1, IRF8, and BATF form a TF complex which binds to *Il9* locus and regulate Th9 differentiation.^101^ In addition, STAT5 phosphorylation induced by IL-2, TSLP, and TL1A promotes Th9 cell development.^99^ The differentiation of Th9 cells is also regulated by costimulation signaling (CD28, OX40, GITR, Notch, and DR3) and other cytokines (IL-1, IL-25, IL-7, and IL-21).^91,99,100^

Th17 cells, characterized by expression of featured cytokines IL-17A-F, IL-21, IL-10, IL-23, and IL-22, and steroid receptor–type nuclear receptor RORγt as the master TF,^102^ contribute to protection against extracellular pathogens, especially at mucosal tissue,^103^ as well as chronic inflammation and autoimmune diseases.^104^ IL-6 and TGF-β drive Th17 cell differentiation while IL-21 and IL-23 stabilize Th17 lineage.^105–109^ IL-6 prompts the expression of RORγt by phosphorylation of STAT3, while inhibits the expression of Foxp3 induced by TGF-β.^110^ RORγt induces the expression of IL-17A, IL-17F, IL-22, and IL-23R by directly targeting to their promoters.^111^ TGF-β signaling through Smad2/3 could sustain STAT3 activation.^112^ Autocrine IL-21 activates STAT3 through Janus kinase (JAK)1/3 activation, which can further increase the expression of IL-23R and confer IL-23 responsiveness of Th17 cells.^113^ IL-23 then enhances STAT3 activation to stabilize Th17 development.^114^ Recent studies have revealed a great degree of plasticity of Th17 cells depending on the presence of TGF-β. TGF-β and IL-6 induce the “classical” Th17 cells characterized by the production of IL-17, IL-21, and IL-10, whereas IL-6, IL-1β, and IL-23 induce “pathogenic” Th17 cells producing high levels of IFN-γ, GM-CSF, and IL-22.^115–117^ Besides RORγt, TCR signal induced transcriptional complex formed by IRF4 and BATF contributes to the initial chromatin accessibility of Th17-related genes such as *Il17*, *Il21*, *Il23r*, and *RORc*, as well as Foxp3 suppression.^118–120^ Runx1 enhances Th17 development through both inducing and directly interacting with RORγt.^121,122^ Other TFs, including RORα, c-Maf, p65, NFAT, and c-Rel, also participate in Th17 differentiation.^123–127^

Tfh cells are specialized CD4^+^ Th cells involved in supporting humoral immune responses by promoting B cell proliferation and maturation, germinal center (GC) response, and high-affinity antibody production.^80,128,129^ Tfh cells are featured by high expression of surface markers PD-1 and CXCR5, costimulatory receptors CD40, CD40LG, and ICOS, cytokines IL-4 and IL-21, signaling molecules SAP, as well as TF STAT3 and Bcl-6.^128^ Tfh cells play central roles in regulating antibody responses during infectious diseases, allergy, autoimmune diseases, and vaccination.^130–132^ Tfh cell development is mainly regulated by the master TF Bcl-6^133^ which primarily represses alternative, non-Tfh, cell fates.^134–136^ Bcl-6 constrains Th1, Th2 and Th17 cell differentiation by repressing their lineage-defining TFs T-bet, GATA-3, and RORγt expression.^133,137,138^ Suppression of B lymphocyte induced maturation protein 1 (Blimp-1, encoded by *Prdm1*) by Bcl-6 is also required for Tfh lineage.^139^ TCF-1 is involved in early induction of Bcl-6 by orchestrating with LEF-1.^140,141^ Other TFs, such as BATF, STAT1/3/4/5, Foxp1, KLF2, IRF4, Ets1, BACH2, Ascl2, Tox2, and Bhlhe40, have been also identified in regulating Tfh cell development.^136,142–144^ Additionally, Tfh cell development is regulated by costimulatory signaling in which CD28 stimulation activates ERK to suppress Tfh cell differentiation,^145^ whereas ICOS activates PI3K to promote and maintain Tfh cells.^146^ In terms of the driver cytokines for Tfh cells, IL-6 and IL-21 promote the differentiation of Tfh cells by acting STAT3 and inducing Bcl-6 expression, respectively.^147,148^ However, IL-2/STAT5 signaling strongly inhibits Tfh development by inducing Blimp-1 expression.^149,150^

T_reg_ cells are a specialized CD4^+^ T cell subset for maintaining immune tolerance by suppressing an immune response. T_reg_ cells are characterized by high expression of IL-2 receptor alpha chain (IL-2Rα, CD25), inhibitory cytokines IL-10, TGF-β, and IL-35, and master TF Foxp3.^151,152^ Two major subsets of T_reg_ cells are identified based on their developmental origin: thymic T_reg_ (tT_reg_) cells, also known as natural T_reg_ (nT_reg_) cells that derive from thymus, and induced T_reg_ (iT_reg_) cells that differentiate from conventional CD4^+^ T (Tconv) cells in the periphery after antigen stimulation and in the presence of TGF-β and IL-2.^153,154^ Given the importance of Foxp3, regulation of Foxp3 expression is critical for T_reg_ cell development, maintenance, and function, in which both transcriptional and epigenetic mechanisms are involved.^155–158^ TCR/CD28 stimulation triggers Foxp3 expression by inducing bindings of NF-κB, AP-1 and NFAT to Foxp3 enhancer/promoter regions.^153,159–161^ In addition, TGF-β enhances Foxp3 transcription by inducing bindings of phosphorylated Smad2 and Smad3, as well as forkhead box protein O1 (FoxO1) and FoxO3 to the conserved non-coding sequences (CNSs) region of Foxp3.^162^ As the downstream of IL-2 signaling, STAT5 also increases the expression of Foxp3 through binding to CNS0 and CNS2.^163,164^ Regulation of Foxp3 stability will be further discussed in autoimmune disease section.

## CD8^+^ T cell differentiation and regulation

CD8^+^ T cells play critical roles in fighting against intracellular pathogens as well as eliminating malignant cells in cancer.^165^ Upon antigen stimulation, naïve CD8^+^ T cells undergo robust expansion to give rise to effector and memory T cells. Effector CD8^+^ T cells, known as CD8^+^ CTLs, can directly induce target cell death by the interaction between Fas/Fas ligand, and secretion of cytolytic mediator perforin, which creates pores in the target cells allowing the delivery of granule serine proteases (granzymes), to induce apoptosis. Memory CD8^+^ T cells provide rapid and strong protection upon antigen reencounter, which is critical for effective and long-term immunity. During CD8^+^ T cell differentiation, heterogeneous effector and memory populations have been identified, including short-live effector CD8^+^ T cells (T_E_), exhausted CD8^+^ T cells (Tex), long-live memory CD8^+^ T cells (T_M_), memory precursor CD8^+^ T cells (T_MP_), central and effector memory CD8^+^ T cells (T_CM_ and T_EM_), and tissue-resident memory (T_RM_) cells, which are named by their phenotype, differentiation potential and functionality.^166,167^ Of note, these subsets are produced at different time window and tissue location upon immune challenge, and their differentiation is under orchestrated regulation of TFs, epigenetic modification, and metabolic programs.

### Key transcription factors

**Fig. 3:**
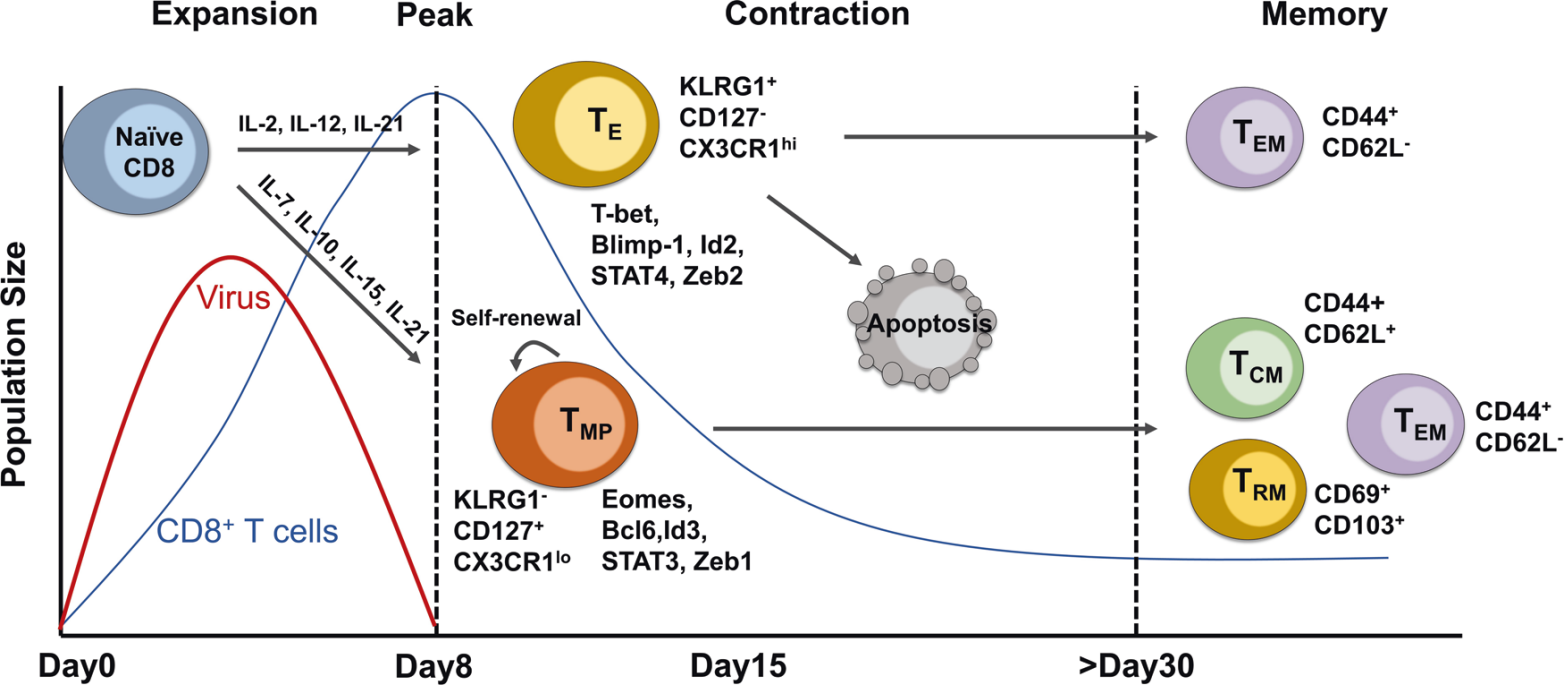
Temporal dynamics of CD8^+^ T cell response in acute infection. The population size of the virus (red line) and CD8^+^ T cells (blue line), as well as CD8^+^ T cell response along with the infection course, are indicated. Upon infection, CD8^+^ T cells undergo robust proliferation and reach the expansion peak on day 8, where the pathogens are rapidly cleared. CD8^+^ T cells at this stage can be separated into T_E_ and T_MP_ populations with distinct surface marker and differentiation potential. The differentiation of effector and memory CD8^+^ T cells is regulated by different transcriptional factors and cytokines. The majority of CD8^+^ T_E_ cells undergo apoptosis at the contraction phase (8–15 days) and leave a subpopulation differentiating into T_EM_, whereas T_MP_ cells keep self-renewal and give rise to T_CM_, T_EM_ and T_RM_ cells over 30 days post-infection

Several key TFs have been well-characterized to control effector versus memory CD8^+^ T cell differentiation in a reciprocal and antagonistic manner (Fig. 3). These TFs include T-bet versus Eomesodermin (Eomes),^168,169^ Blimp-1 versus Bcl-6,^170–172^ Id2 versus Id3,^169,173,174^ STAT4 versus STAT3,^173,175,176^ and Zeb2 versus Zeb1.^177^ While T-bet, Blimp-1, Id2, STAT4, and Zeb2 are predominantly expressed in T_E_ populations and required for effector T cell lineage and/or acquisition of CTL functions, Eomes, Bcl-6, Id3, STAT3, and Zeb1 are enriched in T_M_ populations and support memory T cell formation and maintenance. Those two sets of TFs can either enhance or antagonize each other. For example, Id2 positively regulates T-bet, which induces Zeb2 expression; STAT3 sustains Bcl-6 and Eomes expression; Blimp-1 represses Id3 expression; Bcl-6 can both repress and be repressed by Blimp-1.^169,171^ Currently, collective evidence has supported that the first set of TFs are activated by TCR/costimulatory signals and/or coupled with cytokine signaling (IL-2, IL-12, type I IFN, IFN-γ, IL-21, and IL-27).^170,171,173^ For instance, IL-2 and IL-12 drive effector CD8^+^ T cell differentiation by inducing expression of Blimp-1, T-bet, and Id2 expression.^171^ IFN-α/β stimulates the clonal expansion and production of IFN-γ in CD8^+^ T cells via a STAT4-dependent pathway.^178^ The autocrine IFN-γ further synergizes with IFN-α to promote T-bet expression.^173,179^ Additionally, IL-21 and IL-27 promote Blimp-1 expression in effector CD8^+^ T cells.^180^ The second set of TFs are predominantly driven by cytokine signaling (IL-7, IL-10, lL-15, and IL-21).^169,173,181^ TCF-1 (a downstream factor of the Wnt-signaling pathway) and FoxO1 (a factor related to metabolic pathway) are identified as indispensable TFs for memory CD8^+^ T cell differentiation and maintenance.^182^ It will be interesting to clarify how TCR and cytokine signaling sequentially activate these two sets of TFs and how the cross-regulation occurs among them.

### Epigenetic mechanisms

DNA methylation and histone modifications regulate chromatin accessibility of the regulatory regions of lineage-specific TFs and orchestrate the transcription of key genes to control CD8^+^ T cell development.^183^ DNA methylation, predominantly on CpG islands (CG dinucleotide-sense regions), has repressive effects on gene transcription by hindering the binding of TFs to promoters. During CD8^+^ T cell differentiation, DNA methylation is highly involved in regulating the transcriptional program of effector and memory CD8^+^ T cells.^184–187^ DNA methyltransferase DNMT3A catalyzes DNA methylation at sites such as the promoter of *Tcf7*, thus suppresses memory differentiation and supports effector differentiation.^188^ Methylcytosine dioxygenase TET2 induces DNA demethylation to promote effector differentiation while restrict memory T cell differentiation.^189,190^ In addition, histone modifications has either activating or repressive effects on gene transcription via organizing DNA into structural units termed nucleosomes.^191^ H3K4me3 and H3K9ac are activation-associated modifications, whereas H3K27me3 modification is associated with repressive transcription.^191^ T_E_-associated genes (*Tbx21*, *Prdm1*, *Klrg1*, *Ifng*, *Gzma*, *Gzmb*, and *Prf1*) and T_M-_associated genes (*Foxo1*, *Klf2*, *Lef1*, *Tcf7*, *Il2ra*, *Cd27*, *Ccr7*, and *Sell*) display decreased repressively but increases activating histone modifications during effector or memory lineage differentiation, respectively.^184,186,187,192,193^ Polycomb complex protein BMI1 and histone-lysine N-methyltransferase EZH2, components of the H3K27me3 reader complex, are induced by TCR stimulation and functionally support the expansion, survival and cytokine production of T_E_ population.^193^ Similarly, PR domain zinc finger protein 1 (PRDM1) facilitates effector cell differentiation and suppresses memory lineage through recruiting repressive histone modifiers histone-lysine N-methyltransferase EHMT2 and histone deacetylase 2 (HDAC2) to the *Il2ra* and *Cd27* loci.^194^ Moreover, BATF enhances effector CD8^+^ T cell differentiation by decreasing the expression of histone deacetylase sirtuin 1 (SIRT1) which inhibits T-bet expression though downregulating histone acetylation of the *Tbx21* locus.^195^

### Metabolic regulation

Growing evidence indicates that profound metabolic reprogramming is highly involved in CD8^+^ T cell differentiation. Naïve CD8^+^ T cells primarily depend on basal glycolysis and mitochondrial oxidative phosphorylation to meet their basal cellular processes.^196–199^ T_E_ cells ensure high metabolic flux for the proliferation and functions by upregulating glycolysis^197,199,200^ and glutaminolysis.^201^ Upon TCR and costimulatory stimulation, activation of AKT-mTOR signaling in T_E_ cells upregulates MYC expression, which induces glucose transporter type 1 (GLUT1) expression to promote glucose uptake as well as amino acid transporter SLC32A1/2 expression to increase glutamine uptake.^201–203^ At the same time, NFAT is also induced to upregulate GLUT1/3^204^ and MYC/HIF1α.^197,205^ T_M_ cells differentiate and maintain the population through fatty acid oxidation fueled by long-chain and short/branched-chain fatty acids.^206–208^

During the process of T_E_ towards T_M_ differentiation, the metabolic program turns from an activated status back to a relative quiescent status. T_M_ cells express high level of mitochondrial lipid transporter CPT1A, supporting that lipid oxidation is indispensable for memory T cell differentiation.^209^ In response to IL-15, T_E_ cells upregulate CPT1A expression which mediates the transport of long-chain fatty acids into mitochondria and thereby promotes fatty acid oxidation.^209^ Additionally, short/branched-chain amino acid metabolism, beta-oxidation of 2-methylbutyrate, isobutyrate and isovalerate to generate ATP molecules, play a compensatory role in supporting memory T cell differentiation when long-chain fatty acids become limited.^208^ Upon recall stimulation, T_M_ cells rapidly switch to glycolysis dependent on an epigenetic reprogramming controlled by TCF-1.^210^

Of note, there exists cross-regulations among TFs, epigenetic modification and metabolism.^194,211,212^ TFs and epigenetic modification co-regulate with each other, while they collaboratively regulate metabolic status.^213,214^ These integrated signals are involved in the fate decision and maintenance of CD8^+^ T_E_ and T_M_ populations.

## T cells in acute infection and inflammation

Microbial pathogens including viruses, bacteria, fungi, and protozoa can cause acute and chronic infections in mammalian hosts, leading to various diseases even lethal damage. Owing to advances in public health management and development of vaccination, the number of deaths from pathogenic infection has reduced substantially in recent years. While infectious diseases seem faded out of the public consciousness over the past years, COVID-19 pandemic due to severe acute respiratory syndrome coronavirus 2 (SARS-CoV-2) infection has caused 660 million confirmed cases and 6.6 million deaths by the end of 2022, alerting us to the danger of infectious pathogens. Though innate immune system offers the first-line defense, T cells are crucial in infectious immunity, including efficient clearance of pathogens, helping B cell response and antibody production, rapid control of reinfection, and providing long-term protection by memory formation.

### Effector CD8^+^ T cells contribute to protective immunity during acute infections

CD8^+^ T cells are main responders to viral infection but also participate in defense against bacterial and protozoal pathogens. Effector CD8^+^ T cells secrete pro-inflammatory cytokines such as IFN-γ and tumor necrosis factor (TNF) to inhibit viral replication,^215^ and express various chemokines to attract other inflammatory cells to sites of infection. Acute infections, defined as infections of only a short duration where the pathogens are eliminated rapidly after the peak of the immune response, are caused by infections of Armstrong strain of lymphocytic choriomeningitis virus (LCMV), *Listeria monocytogenes* (LM), influenza virus, hepatitis A virus, and vaccinia virus. The dynamics of CD8^+^ T cell response to acute infections has been studied extensively.^216–218^ The response of antigen-specific CD8^+^ T cells can be roughly divided into distinct stages (Fig. 3): the expansion phase (0–7 days) where CD8^+^ T cells are actively proliferating; the peak of expansion (day 8) where the effector CD8^+^ T cells reach the maximum number and stop proliferating; the contraction phase (8–15 days) where majority of effector CD8^+^ T cells undergo apoptosis; and the memory phase (>30 days) with only a small population of cells are survived and differentiated into distinct types of memory cells: CD44^+^CD62L^−^ T_EM_, CD44^+^CD62L^+^ T_CM_, and CD69^+^CD103^+^ T_RM_.^219^ The fate decision between effector and memory T cells occurs as early as the first division of activated CD8^+^ T cells, in which the daughter cells with high MYC and high canonical BRG1/BRM-associated factor (cBAF) preferentially differentiate into T_E_ cells, whereas those with low MYC and low cBAF develop into T_M_ cells.^220^ At the peak of acute infection, expression of KLRG1 and CD127, the IL-7 receptor subunit-α (IL-7Rα), is used to identify short-lived terminally differentiated effector cells (T_E_, KLRG1^+^CD127^-^) and long-lived memory precursor cells (T_MP_, KLRG1^-^CD127^+^). Besides KLRG1, T_E_ cells express a range of effector molecules including cytotoxic granzymes, perforin, cytokines (IL-2, IFN-γ, and TNF), chemokines (CCL5 and CCL3), and chemokine receptors (CX3CR1, CXCR6 and CCR5). Recently, the expression of chemokine receptor CX3CR1 on CD8^+^ T cells has been used to classify effector and memory T cells.^221^ The level of CX3CR1 on CD8^+^ T cells correlates with the degree of effector differentiation as CX3CR1^hi^ subset contains the terminally differentiated effector T cells.^222^ The differentiation and function of effector/memory CD8^+^ T cells are precisely and elaborately regulated at multiple levels, which have been described in the previous section.

Overall, CD8^+^ T cell responses to different microbial pathogens are similar regarding to the kinetics of T cell expansion and contraction, effector function and regulation, and memory formation. However, CD8^+^ T cell priming, costimulatory signaling, persistence of response and intensity of the inflammation can be different in various pathogenic infections.^223–227^ In the acute phase of SARS-CoV-2 infection, CD8^+^ T cells in severe and convalescent COVID-19 patients exhibit activated phenotypes characterized by elevated expression of CD38, HLA-DR, Ki67, PD-1, perforin, and granzyme B.^228–232^ Comprehensive single-cell RNA-sequencing (scRNA-seq) analysis reveals that SARS-CoV-2-specific CD8^+^ T cells display increased “exhaustion” phenotype with high expression of inhibitory receptors (IRs) (Tim-3 and Lag-3) than influenza A virus- and Respiratory syncytial virus (RSV)-reactive CD8^+^ T cells. Interestingly, such “exhausted” CD8^+^ T cells are not dysfunctional but enriched for cytotoxicity-related genes.^233^ Nevertheless, SARS-CoV-2-reactive CD8^+^ T cells have reduced cytokine production.^233^ Therefore, further studies are needed to fully elucidate the function of SARS-CoV-2-specific CD8^+^ T cells in COVID-19 patients.

### Effector CD4^+^ Th cells in infection

**Fig. 4:**
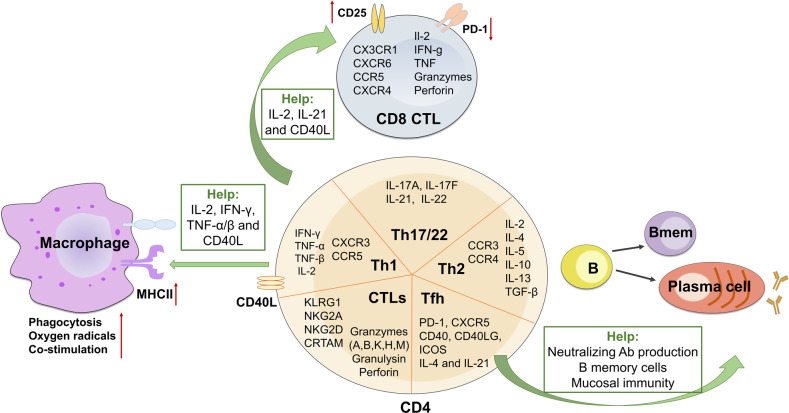
Effector CD4^+^ and CD8^+^ T cells contribute to infectious immunity. In response to infection, naïve CD8^+^ T cells develop into CD8^+^ CTLs expressing a range of chemokine receptors and effector molecules, whereas naïve CD4^+^ T cells develop into distinct Th1, Th2, Th17, Th22, Tfh, and CTL subsets with indicated phenotypes to exert protective functions. In addition, CD4^+^ T cells indirectly contribute to pathogen clearance by providing help to macrophages, CD8^+^ CTLs and B cell and antibody responses

CD4^+^ T cells play multifaceted roles in modulating immune responses (Fig. 4), contributing to protection from a broad range of pathogenic microbes. Th1 and Th2 subsets have been long identified as crucial players in protective immunity against pathogens.^234^ Although effector Th cells found in vivo after infections are often heterogeneous populations, CD4^+^ T cells in response to viruses mainly display Th1-associated phenotypes.^235^ Particularly, enriched Th1 lineage is a typical feature of pulmonary infections and plays crucial roles in fighting against *Mycobacterium tuberculosis* (Mtb), influenza virus, *Staphylococcus aureus (S. aureus)*, Middle East respiratory syndrome coronavirus (MERS-CoV), SARS and SARS-CoV-2.^236–239^ Th1 cells, characterized by expressing cytokines IFN-γ, TNF-α/β and IL-2, chemokine receptors CXCR3 and CCR5, and TFs T-bet and STAT4, mainly fight intracellular pathogens of viruses, bacteria, fungi and protozoa.^76^ By contrast, Th2 cells, expressing cytokines IL-2, IL-4, IL-5, IL-10, IL-13, chemokine receptors CCR3 and CCR4, and TFs GATA-3 and STAT6, are strong drivers of humoral immune reactions against extracellular helminthic parasites and allergic inflammation.^240,241^

Th17 response, featured by massive pro-inflammatory cytokine production, is often elicited together with Th1 cells in infections by bacterial and viral microorganisms, such as Mtb,^242^ *S. aureus*,^243^ MERS-CoV,^244^ Dengue virus,^245^ RSV,^246^ hepatitis B virus (HBV)^247^ and SARS-CoV-2.^248^ Additionally, fungal microbes, such as *Pneumocystis carinii* and *Candida albicans* can trigger strong Th17 response by inducing large amounts of IL-23 which is the key cytokine for full Th17 differentiation and function.^102,249,250^ Furthermore, Th22 cells are a newly identified Th subset producing IL-22 but not IFN-γ, IL-4, or IL-17.^251^ Th17/Th22-related cytokines can target on diverse cell types, including non-immune cell populations, such as epithelial cells, fibroblasts, and endothelium cells. Hence, Th17 and Th22 cells tend to protect against infections locally on the mucosal tissue and skin, respectively.^252,253^ IL-17 and IL-22 corporately augment the host immunity against infections at mucosal sites via promoting antimicrobial peptides production by mucosal epithelium and recruitment of neutrophils to eliminate bacteria and fungi.^254^

Moreover, CD4^+^ CTLs contribute to pathogen clearance through direct cytolytic activity.^77,255,256^ This subset of CD4^+^ T cells attracts much attention recently owing to their important functions in protecting against infectious disease, promoting human longevity, and mitigating tumor progression.^257–259^ CD4^+^ CTLs have been largely observed in both human and mice infected with viruses,^235^ such as cytomegalovirus (CMV),^260^ human immunodeficiency virus (HIV)-1,^261^ hepatitis viruses (HBV, HCV and HDV),^262^ Epstein–Barr virus (EBV),^263^ Dengue virus,^264^ influenza virus,^265,266^ and SARS-CoV-2.^267^ CD4^+^ CTLs are characterized by expression of KLRG1, natural killer group 2 (NKG2A), NKG2D, the class I-restricted T cell-associated molecule (CRTAM) and downregulated CD27/CD28.^77,256^ The cytotoxic activities of CD4^+^ CTLs attribute to the expression of pro-inflammatory cytokines, perforin, granzymes (A, B, K, H, and M), granulysin, and death receptor-dependent signaling (Fas and TRAIL).^268–270^ The transcriptional regulation of CD4^+^ CTLs is highly comparable to that of CD8^+^ CTLs, in which TFs T-bet, Eomes and Runx3 play critical roles in driving CD4^+^ CTL programming while ThPOK expression limits cytotoxic functions in CD4^+^ T cells.^271–273^ Additionally, IL-2 could drive the cytolytic phenotype of CD4^+^ CTLs,^274^ while pro-inflammatory cytokines IL-12, IL-6, and IFN-α increase granzyme B and perforin production and target killing activity.^275^ It remains unclear about the precursors of CD4^+^ CTLs or whether this population is merely the terminal differentiated Th1 cells. However, more evidence claims that CD4^+^ CTLs are a separate Th subset in regards to its differentiation trajectory, effector function and regulatory networks.^255,276,277^ Furthermore, heterogeneous populations within CD4^+^ CTLs have been identified in viral infection.^277,278^ In general, CD4^+^ CTLs are highly associated with antiviral immunity, however, aberrant CD4^+^ CTL activity has also been linked with immunopathology in some settings.^279–281^ For example, CD4^+^ CTLs contribute to the disease severity during SARS-CoV-2 infection^267,282^ and lung fibrosis.^267,283^

Accumulating evidence has suggested that more than one type of Th subsets can be triggered during the infection, and both synergy and balance among Th cells contribute to infection control. For instance, costimulation of Th1, Th2 and Th17 responses is commonly observed in various microbial infections, such as Mtb,^284,285^ *Echinococcus multilocularis*,^286^ *Aspergillus fumigatus*,^287^ HIV,^288^ SARS-CoV-2.^248^ Meanwhile, T_reg_ cells can be induced during infection to prevent overstimulation of immune response and “self-attacking”.^289–292^ During Mtb infection, activation of macrophages induced by Th1-derived IFN-γ is crucial to control the tuberculosis. However, persistent Th1 response and pro-inflammatory cytokines can cause lung fibrosis and necrosis. Th2 cytokines IL-4, IL-10, and TGF-β are prominent to prevent pathology induced by aberrant Th1 response.^293^ Enhanced Th2 response during SARS-CoV-2 and influenza infection is associated with severe disease symptoms by inhibiting antiviral responses.^241^

### Effective control of infection relies on CD4^+^ T cell help

CD4^+^ Th cells are indirectly involved in pathogen control by regulating functions of other immune cells, such as activating innate immune populations, assisting CD8^+^ CTL response and B cell maturation and antibody production (Fig. 4). CD4^+^ T cells, mainly Th1 population, are central for activation of pro-inflammatory macrophages by releasing cytokines IL-2, IFN-γ, and TNF-α/β and expressing CD40L.^76^ Activated macrophages augment their antimicrobial effectiveness by increasing microbial phagocytosis, production of nitric oxide (NO) and oxygen radicals, expression of MHC class II molecules and a number of costimulatory molecules for effective antigen presentation to T cells.^294^ Activated macrophages are also important for efficiently eliminating intracellular pathogens such as mycobacteria which grow primarily inside of macrophages and are shieled from CTLs and neutralizing antibodies.^295^

Furthermore, CD4^+^ T cell help is essential for optimal and effective CD8^+^ T cell response,^51^ although the requirement for primary CD8^+^ T cell response remains controversial. Some studies have shown that in the absence of CD4^+^ T cells, the primary CD8^+^ T cell expansion and cytotoxic functions during LCMV and LM infection are unaffected.^296,297^ However, other studies have reported that CD4^+^ T cells, particularly their memory subset, are required for primary effector CD8^+^ T cell response to herpes simplex virus (HSV) and influenza virus.^298–301^ The controversial effects of CD4^+^ T cell help for primary CD8^+^ T cell response are likely derived from different help-evaluation models.^301^ On the other hand, profound and consistent evidence indicates that CD4^+^ T cell help is indispensable for memory CD8^+^ T cell generation and their recall response to antigen restimulation.^302–304^ Mechanistically, CD4^+^ T cells support CD8^+^ T cell responses via cytokines IL-2 and IL-21, and CD40L signaling.^301,305–307^ Additionally, CD4^+^ T cells have been shown to help CD8^+^ T cells by enhancing their CD25 expression and downregulating PD-1 expression.^308,309^

CD4^+^ Tfh cells are essential for B cell responses and generating protective antibodies against viral, bacterial, parasite, and fungal pathogens in mice, non-human primates, and humans.^131,310^ The protective effects of Tfh cells on humoral immunity attribute to multiple mechanisms.^132^ First, Tfh cells help the production of protective antibodies that directly neutralize pathogens and inhibit their replication, and indirectly promote pathogen clearance through antibody opsonization. Tfh cells have long been known to highly correlate with broadly neutralizing antibodies in HIV infection.^311^ During SARS-CoV-2 infection, increased circulating Tfh (CCR7^lo^PD-1^+^ICOS^+^CD38^+^) cells and production of neutralizing antibodies were observed in COVID-19 convalescent individuals and associated with mild symptoms.^312,313^ In contrast, defective Tfh cell response and delayed development of neutralizing antibodies were found in deceased patients.^314^ Second, Tfh cells support memory B cell formation and response, which is important for rapid humoral response upon reinfection. Thirdly, Tfh cells in mucosal-associated lymphoid tissue (MALT) can also promote IgA production and function to modulate respiratory and gastrointestinal-tract infections.^315^ Collectively, CD4^+^ T cells are crucial mediators for supporting, promoting, and regulating both humoral and cellular immunity to resolve the infections effectively.

## Chronic infection and cancer: persistent antigenic stimulation

In contrast to acute infections, antigen stimulation is persistent in chronic infection and cancer. It is now well-accepted that most T cells in such circumstances adopt a unique differentiation trajectory—exhaustion.^316,317^ Exhausted T (Tex) cells have been identified in many high grade chronic viral infections, such as HIV, HBV, HCV, and LCMV-Clone 13 strain,^318–321^ and in almost every mouse and human cancer.^322,323^ A wealth of recent studies at single-cell level have revealed that Tex cells constitute heterogenous populations with distinct transcriptional, epigenetic and functional signatures, playing critical roles in protecting against infections and tumors. The discovery of stem-like progenitor CD8^+^ Tex (Tpex) cells, the main responder to immune checkpoint blockade (ICB), attracts a large attention in both preclinical and clinical research field for developing next-generation cancer immunotherapies.^322,324^ In this section, we will summarize current understandings of the cellular and functional features of CD8^+^ and CD4^+^ T cells in chronic infection and tumor, their developmental pathways, regulatory mechanisms, CD4^+^ T cell help for CD8^+^ CTL responses, as well as contributions to anti-tumor immunity and checkpoint blockade.

## Exhausted CD8^+^ T cells

Exhausted CD8^+^ T cells represent an entirely distinct differentiation trajectory with unique cellular phenotype, heterogeneity, and functional capacity.^219,325,326^ Along with the exhaustion, CD8^+^ T cells gradually lose production of IL-2 and TNF-α, and cytotoxic function.^327^ Compromised IFN-γ production occurs at more later stage of exhaustion and is associated with terminally differentiated Tex.^328^ But terminal CD8^+^ Tex may retain the ability to degranulate and produce chemokines and cytokines, such as MIP1α, MIP1β, RANTES, and IL-10 ^329^. Different from T_M_ cells in acute infection that undergo steady homeostatic self-renewal responding to cytokines IL-7 and IL-15,^330^ Tex cells display defects in responsiveness to homeostatic cytokines due to impaired IL-7Rα and IL-2/15Rβ signaling pathways.^331,332^ Instead, persisting antigen stimulation drives a proliferative progenitor pool of Tex cells,^333,334^ that Tex cells adopt a self-renewing mechanism dependent on continuous TCR stimulation.^333^ In addition, a key hallmark of CD8^+^ Tex cells is the upregulated and sustained expression of multiple IRs, such as PD-1, CTLA-4, Lag-3, TIGIT, Tim-3, CD39, 2B4, CD160, etc.^329,335^ The extent and coexpression of IRs directly correlate with the severity of exhaustion.^335,336^ On the other hand, Tex cells also express costimulatory molecules which, however, favor T cell exhaustion during chronic infection and tumor. For example, costimulation of CD27 and CD28 results in an enhanced T cell exhaustion.^337,338^ CD28 signaling is compromised due to loss of competition to CTLA-4 for B7 family ligands.^338^ PD-1 signaling further suppresses T cell function by specifically inducing CD28 dephosphorylation.^339^

### Heterogeneity and differential trajectory of CD8^+^ Tex cells

The exhaustion/dysfunction of CD8^+^ T cells in chronic infection is established progressively with sequential phases.^340,341^ Analysis of CD8^+^ cell chromatin states define two discrete dysfunctional states: early reprogrammable and late non-reprogrammable T cells that the former ones are plastic and retain the potential to form memory after adoptive transfer, whereas the latter are fixed dysfunction with massive IR expression.^341,342^ Regarding to Tex cell origin, it was pointed out that CD8^+^ Tex cells arise from the same pool of KLRG1^-^CD127^+^ T_MP_ cells in acute infection.^343^ The differentiation divergence of virus-specific CD8^+^ T cells responding to acute and chronic viral infections occurs as early as 4.5 days post-infection.^344^ However, under persistent antigen stimulation, these precursors progressively lose memory potential and develop into Tex cell state.^342,343^ With the rapid development of single-cell technologies, extensive analysis of tumor infiltrating lymphocytes (TILs) reveal a diverse spectrum of exhausted CD8^+^ T cells in non-small cell lung cancer (NSCLC), melanoma, breast cancer, liver cancer, and colorectal cancer.^324,345–351^

**Fig. 5:**
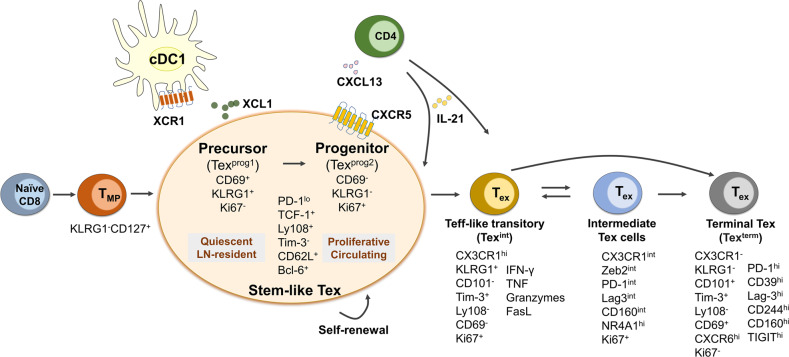
Heterogenous populations and differential trajectory of CD8^+^ Tex cells in chronic infection and tumor. Under persistent antigen stimulation, CD8^+^ T cells adopt an exhaustion differentiation trajectory of naïve → T_MP_ → stem-like Tpex → effector-like transitory → intermediate → terminal Tex cells. Expression of signature markers and effector molecules at each Tex population is indicated. The stem-like Tpex cells are further divided into early precursor and late progenitor stages with discrete phenotype, proliferative status and preferential location. Tex subsets identified from different studies may use different names which are marked in the parentheses. CXCL13 and IL-21 derived from CD4^+^ T cells are critical for differentiation of CXCR5^+^ Tpex cells and CX3CR1^+^ Teff-like transitory Tex cells, respectively. CD8^+^ Tpex cells interplay with cDC1s through XCL1/XCR1 axis

The CD8^+^ Tex cells being a distinct differentiation trajectory largely attributes to the identification of the stem-like, self-renewing Tpex population which is marked by expression of TCF-1 and surface profile of PD-1^lo^Tim-3^-^Ly108^+^CXCR5^+^.^340,352^ TCF-1-expressing Tpex cells are responsible for the maintenance of Tex cell populations in chronic viral infection and tumor.^353,354^ Tpex cells adopt a branched differentiation paradigm (Fig. 5), where they both self-renew and give rise to terminally differentiated exhausted T cells.^334,344^ Despite sharing similar phenotypes, the stem-like Tpex cells can be further separated into early precursor and late progenitor stages: the CD69^+^KLRG1^+^Ki67^-^ CD8^+^ Tex precursors are more quiescent, lymph node (LN)-resident and having a baseline level of proliferation, whereas CD69^-^KLRG1^-^Ki67^+^ progenitors have robust proliferation and access to circulation.^352,355^ Recently, more markers are identified to define Tpex subsets. Tsui et al. reported that a small subset of TCF-1^+^CD62L^+^ Tpex cells are the stem-like population essential for long-term self-renewal, maintenance of Tex lineage and responsiveness to immunotherapy.^356^ In human individuals experienced latent infection such as CMV or EBV, TCF-1^+^ progenitors are comprised of two subsets based on PD-1 and TIGIT expression. The PD-1^-^TIGIT^-^ progenitors are committed to a functional Tex differentiation, whereas PD-1^+^TIGIT^+^ progenitors are differentiated into a dysfunctional and exhausted state.^357^ Additionally, XCL1 is found expressed in CD8^+^ Tpex cells and associated with XCR1^+^ conventional type I dendritic cells (cDC1s).^358^

Persistent antigen exposure induces downregulation of TCF-1, and drives Tpex differentiation into a “transitory” effector state and terminal exhausted T cells (Fig. 5). The transitory effector T (Teff)-like cells are critical for viral and tumor control and characterized by expression of chemokine receptor CX3CR1, producing IFN-γ, TNF and granzyme B, and enhanced cytotoxicity and cell proliferation.^359,360^ Generation of CX3CR1^+^ subset strongly depends on CD4^+^ T cell help and IL-21.^360,361^ Hudson et al. propose that Tpex differentiation follows a linear developmental trajectory where Tpex cells generate CX3CR1^+^Tim-3^+^CD101^-^ transitory Teff-like T cells that further give rise to CX3CR1^-^Tim-3^+^CD101^+^ terminal Tex cells.^359^ Similarly, the expression of Ly108 and CD69 defines four subsets of Tex cells with a hierarchical developmental progression from Ly108^+^CD69^+^ (referred to Tex^prog1^) to Ly108^+^CD69^−^ (Tex^prog2^) to intermediate differentiated Ly108^−^CD69^−^ (Tex^int^) cells and the most terminally differentiated Ly108^−^CD69^+^(Tex^term^) subset.^355^ Of note, Tex^int^ cells share similar transcriptional program to the CX3CR1^+^ Teff-like Tex cells identified in previous studies.^359,360^ Recently, a novel Tex subset expressing NK-associated genes (NKG2A and CD94) was uncovered within the Tex^int^ cell population.^362^ More evidence supporting the Tex cell differentiation trajectory comes from comprehensive analysis of antigen-specific T cells in patients with human papillomavirus (HPV)-positive head and neck cancer. Paired scRNA-seq analysis and TCR sequencing of HPV-specific CD8^+^ T cells sorted by MHC class I tetramers revealed that antigen-specific PD-1^+^TCF-1^+^ stem-like CD8^+^ T cells could proliferate and differentiate into Teff-like transitory and terminally differentiated cells.^363^ In addition, epigenetic landscape analysis demonstrates that the phenotypic changes of Tex cell development coincide with the chromatin accessibility of key genes.^355,359^ Long-term antigen stimulation leads to epigenetic reprogram which enforces the terminal exhaustion of T cells marked by high expression of IRs, diminished effector-related molecules (IFN-γ, TNF, granzymes, and T-bet) and loss of stemness and proliferation potential (TCF-1, MYB, MYC, and Ki67).^219,355,359^ Furthermore, in infection with chronic LCMV-Clone 13, a “bridging population” between Teff-like transitory and terminal exhausted Tex cells is characterized by intermediate expression of CX3CR1, Zeb2 and IRs, but high expression of NR4A1 (encoding NUR77), suggesting a recent activation by TCR stimulation.^364^ Chemokine receptors CXCR6 and CX3CR1 can be used to discriminate these three populations: Teff-like transitory cells (CX3CR1^hi^), intermediate Tex cells (CX3CR1^int^) and terminal exhausted Tex cells (CX3CR1^lo^CXCR6^hi^).^364^ Recent high-dimensional single-cell multi-omics have revealed more heterogenous Tex clusters with distinct phenotypic, transcriptomic, epigenetic and functional patterns, which also display disease- and tissue-specificity.^364–366^ It is noteworthy that exhausted T cells can be also induced in acute infection with strong T cell stimulation. For instance, severe acute respiratory syndrome elicited during SARS-CoV-2 infection induces T cell exhaustion phenotypes with high level of IRs expression.^229,233^

### Transcriptional and epigenetic regulation of CD8^+^ Tex cells

The differentiation of CD8^+^ Tex cells is tightly controlled by transcriptional and epigenetic networks. In chronic infection and tumors, TCF-1 identifies the stem-like CD8^+^ Tpex cells.^354,367,368^ Accordingly, mice with *Tcf7* deficiency could not develop stem-like Tpex cells and Tex populations,^353^ whereas overexpression of *Tcf7* led to enhanced Tpex program as well as antiviral and anti-tumor immunity.^369^ TCF-1 plays central roles in Tpex cells by organizing transcriptional regulatory networks.^354,370^ TCF-1 coordinates with FoxO1 which also acts as an upstream regulator of TCF-1 expression to promote and maintain the stemness in CD8^+^ T cells by augmenting pro-memory TFs Eomes, Id3, c-Myc, Bcl-2, and Bcl-6 expression while inhibiting effector-related TFs T-bet, Id2, Runx3, and Blimp-1.^367,368,370–372^ MYB (also known as c-Myb) is a pivotal TF for CD8^+^ central memory and Tpex cell generation and maintenance by acting as a transcriptional activator of *Tcf7*.^356,373^ Moreover, BACH2 promotes stem-like CD8^+^ T cell commitment in chronic infection and cancer by enforcing the transcriptional and epigenetic programs.^374^

TOX, a high-mobility group box DNA-binding protein, has recently emerged as a critical regulator for Tex cell programs.^375–377^ Enforced expression of TOX is sufficient to induce an exhausted T cell-associated transcriptional program with increased expression of IRs.^376,378^ While TOX deficiency has no impact on CD8^+^ T cells differentiation and effector function in acute infections, deletion of TOX in tumor-specific T cells inhibits the upregulation of IRs and augments the cytokine production, effector functions, and TCF-1 expression.^375,378^ Although TOX deficient T cells display a “non-exhausted” immunophenotype, those T cells remain hyporesponsive and ultimately diminish.^375,378^ In fact, TOX deficient CD8^+^ T cells fail to persist and differentiate into Tex cells, indicating that TOX-regulated exhaustion indeed protects T cells from overstimulation and activation-induced cell death.^375,376,378^ Additionally, TOX and nuclear receptor NR4A form positive feedback loops to impose CD8^+^ T cell dysfunction and exhaustion.^379–381^ BATF is another important TF regulating T cell exhaustion, however, its role remains controversial. Some studies report that BATF facilitates viral clearance by driving the transition from TCF-1^+^ Tex progenitors to CX3CR1^+^ effector cells during chronic viral infection.^382^ Moreover, BATF cooperates with IRF4 to resist exhaustion; overexpression of BATF promotes the survival and anti-tumor immunity in chimeric antigen receptor (CAR) T cells.^383^ However, others claim that BATF drives T cell exhaustion by directly upregulating exhaustion-associated genes, thus BATF depletion could significantly enhance T-cell resistance to exhaustion and exhibit superior efficacy against solid tumors in CAR-T cells.^384–386^

Intriguingly, Tex cells express certain TFs shared by T cells in acute infection, but with distinct gene transcription,^387^ suggesting context-dependent functions of these TFs. For instance, Eomes and T-bet are dually required for Tex cell generation.^334^ Eomes expression is elevated in tumor-infiltrating CD8^+^ T cells and high level of Eomes promotes exhaustion.^388,389^ But high expression of T-bet was found associated with Tpex and effector-like Tex subset.^334,390,391^ In addition, TF NFAT family which has a well-established role in mediating T cell activation when partners with AP-1,^392^ has been shown to regulate Tex cell differentiation. NFATc1 drives exhaustion program by promoting IR expression,^393^ whereas NFATc2 prevents the dysfunction of CD8^+^ Tex cells.^394^ The major differences of CD8^+^ T cells in acute and chronic infections are compared (Table 1).

**Table 1:** Characteristics of CD8^+^ T cells in acute and chronic infections

Infection type	Infectious gents/condition	Characteristics	Stages	Fate	Subsets	Surface marker	Key TF	Refs
Acute	LCMV-Armstrong, LM, influenza virus, HAV, RSV, vaccinia virus	IFN-γ, TNF, IL-2, KLRG1, Granzymes, Perforin	expansion, contraction, memory	T_E_, T_MP_, T_EM_, T_CM,_ T_RM_	T_E_	KLRG1, CX3CR1, CXCR6, CCR5	T-bet, Blimp-1, Id2, STAT4, Zeb2	^169,173,182,193,219,223^
					T_MP_	CD127, CD62L	Eomes, TCF-1, FoxO1, Bcl-6, Id3, STAT3, Zeb1	
Chronic	LCMV-Clone 13, HIV, HBV, HCV, CMV, EBV, SARS-CoV-2, Cancer	Loss of IL-2, IFN-γ, TNF-α; Expression of IRs (PD-1, CTLA-4, Lag-3, TIGIT, Tim-3, CD39, 2B4, CD160)	Tex precursor, Tex progenitor, Teff-like transitory, Intermediate Tex, Terminal Tex	Tex	Teff-like	KLRG1, CX3CR1, Tim-3	T-bet, Id2, Runx3, Blimp-1	^219,323,325,326,340,355,359,367,370,377,387^
					Tpex	Ly108, CD62L, CXCR5, XCL1	TCF-1, FoxO1, Eomes, Id3, c-Myc, Bcl-2, Bcl-6, MYB, BACH2	
					Tex	PD-1, CD101, Tim-3, CXCR6	TOX, BATF, Eomes, T-bet, NFAT	

The underlying mechanisms that govern the distinct transcriptional features of Tex cells remain poorly understood, but at least partially, are controlled by epigenetic programming. CD8^+^ Tex cells exhibit a unique chromatin landscape different from effector and memory T cells.^342,355,362,395^ The chromatin accessibility of key exhausted-associated genes such as TCR signaling, cytokines, costimulatory and coinhibitory receptors has experienced dynamically epigenetic reprogram.^365,396^ For instance, the gene regions around *Tcf7* and *Id3* are more accessible in stem-like Tpex cells while that in *Prdm1, Id2*, and *Pdcd1* are more accessible in exhausted CD8^+^ T cells.^397,398^ Particularly, TOX acts as a crucial regulator of epigenetic programming of CD8^+^ Tex cells by repressing the chromatin accessibility of genes involved in effector cell differentiation. Additionally, TCF-1 regulates gene transcription by altering the three-dimensional (3D) genome organization.^399,400^ A prominent feature of Tpex cells is that the exhaustion commitment can be transmitted to their progeny even when adoptive transferred into new hosts received acute infection.^401^ The underlying mechanisms of such exhaustion inheritage are derived from epigenetic imprints which once are established, they can not be reversed by change of exogenous environment or by PD-(L)1 blockade.^402–404^

### Tex subsets contributing to anti-tumor immunity and ICB

Tumors with high infiltration of T cells are generally considered as immune-inflamed or “hot” tumors. However, intratumoral T cells may not be tumor-reactive. TCR repertoire analysis reveals that the tumor recognizing T cells were limited to merely 10% of intratumoral CD8^+^ T cells.^405^ ICB can robustly reinvigorate Tex cell function, making it one of the most promising cancer therapies in the clinic.^406–408^ Antibodies targeting IRs on tumor-infiltrating T cells, such as PD-1/PD-L1 (among others), have been demonstrated impressive clinical activities across a variety of cancer types. Despite large success, ICB faces clinical challenges of low responsive rate, drug resistance, and immune-related adverse events (irAEs).^409,410^ Thus, it is of great significance to understand which subset of CD8^+^ T cells respond to ICB and how. Among heterogenous CD8^+^ Tex cells, it is now well-appreciated that the PD-1^+^TCF-1^+^ stem-like Tpex cell population mainly mediates tumor responses to checkpoint blockade.^353,410,411^ Comparison between the responder and non-responder of melanoma patients receiving ICB treatment demonstrates that the frequency of TCF-1^hi^ tumor-infiltrating CD8^+^ T cells predicts positive clinical outcome.^412^ This CD8^+^ Tpex cell population has also been observed in human NSCLC, colorectal cancer, HPV-positive head and neck cancer and bladder cancer, and their number was augmented following ICB treatment.^363,411,413,414^ Interestingly, ICB could control tumor growth in mice depleted TCF-1-expressing T cells, indicating that later differentiated Tex cells may also be targeted by ICB.^411^ Indeed, comprehensive transcriptomic and TCR clonal analysis reveal that tumor/ICB-responsive CD8^+^ T cells including neoantigen-specific ones exhibit enhanced exhaustion compared to non-tumor-reactive bystander CD8^+^ T cells.^415,416^ Accordingly, differentiation from TCF-1^+^ Tpex cells into late stage of Tex cells expressing PD-1 and Tim-3 favors the tumor control.^417,418^ Thus, high expression of PD-1 and/or CTLA-4 on tumor infiltrating CD8^+^ T cells provides a predictive biomarker for responsiveness to ICB therapy.^419,420^

Beyond, it is also critical to address the effects of ICB on CD8^+^ T cell state. It has been shown that effective immunotherapies can induce remarkable remodeling of tumor environment (TME) and systemic immune activation in multiple tissues.^421^ Paired scRNA-seq and TCR-seq on tumor biopsies from NSCLC patients revealed that the Tpex population was accumulated in responsive tumors but not in non-responsive ones after anti-PD-1 therapy.^422^ The data also depicts that the increased Tpex cells are mainly derived from local expansion or replenishment from peripheral T cells with pre-existing clonotypes, a phenomenon called “clonal revival”.^422^ While the effect of ICB primarily relies on pre-existing state of intratumoral T cells, ICB can alter the TCR repertoire to generate novel T cell clonotypes, which is referred to as “clonal replacement”.^422,423^ Moreover, intratumoral exhausted T cell populations and their immunological responses to ICB exhibit features of spatial distribution.^424^ Studies in both mouse and human tumors have demonstrated that tumor-draining LNs (TdLNs) are the preferential reservoirs for TCF-1^+^ Tpex cells that remain stable regardless of the changes in TME and sustain continuous development of anti-tumor T cells.^425,426^ Blockade of sphingosine 1-phosphate receptor 1 (S1P1)-mediated T cell egress from TdLNs remarkably decreased the frequency of intratumoral CD8^+^ Tpex cells and the tumor eradication efficacy of anti-PD-1 therapy.^421,426^ The clonal overlapping between tumor-infiltrating CD8^+^ T cells and proliferating CD8^+^ T cells in the circulation in cancer patients following anti-PD-1 treatment highly suggests a recruitment from secondary lymphoid organs.^427^ A group of bona fide tumor-specific memory CD8^+^ T cells within TdLNs are important responders to PD-1-based ICB, highlighting their potentials in anti-tumor immunotherapy.^428^ Inherent in this theory, local (intratumoral, intradermal or intrapleural) administration of ICB antibodies, compared to systemic (intravenous or intraperitoneal) injection, results in enhanced tumor regression due to antibody accumulation and Tpex cell expansion within TdLNs.^429,430^

## Complex CD4^+^ T helper cells

Robust and functional CD4^+^ T cell responses are essential for effective pathogen clearance and tumor eradication. Compared to well-defined CD8^+^ Tex cell differentiation, the cellular and functional signatures of CD4^+^ T cells in chronic disease settings are little characterized, especially with the complexity of multiple Th subsets. CD4^+^ T cells play multifaceted roles in chronic infection and tumor: constituting both favorable and deleterious subsets, enhancing CD8^+^ T cell function, and responding to ICB,^427,431^ which highlights potential next-generation therapeutics of harnessing CD4^+^ T cell function.

### Are CD4^+^ T cells exhausted?

The effects of persistent antigenic stimulation on CD4^+^ T cell phenotype, differentiation and function remain less understood. Whether CD4^+^ T cells become “exhausted” during chronic infection remains a question for a long time. Controversial results were obtained as viral-specific CD4^+^ T cells lose effector function and produce reduced IFN-γ, TNF-α and IL-2 during chronic infection,^432,433^ but the production of IL-10 and IL-21, the important cytokines in chronic infection for sustaining CD8^+^ T cell and B cell responses,^434–436^ are increased.^434,437,438^ Transcriptional analysis of CD4^+^ T cells during chronic (LCMV-Clone 13) infection has demonstrated a unique exhaustion-associated molecular and transcriptional profile, which is distinct from CD8^+^ Tex cells and effector or memory CD4^+^ T cells in acute (LCMV-Armstrong) infection.^439^ In addition to reduced cytokine production, CD4^+^ Tex cells express markedly upregulated IRs including PD-1, CTLA-4, CD200 and BTLA, and costimulatory receptors OX40, CD27 and ICOS.^439^ Core TFs involved in CD4^+^ Tex cells include Eomes, Blimp-1, Helios, Klf4, and T-bet.^439^ During LCMV-Clone 13 infection, viral-specific CD4^+^ T cells formed multiple clusters which could be broadly grouped into Th1, Tfh and Th1/Tfh hybrid clusters at different stages, suggesting an altered Th lineage differentiation in chronic infection.^431^ Notably, persistent viral infection drives a progressive loss of Th1 response likely due to PD-1/PD-L1 inhibitory signaling pathway,^431,440^ but skews CD4^+^ T cells toward Th2, Th17, T_reg_, Tfh, and allergic CD4^+^ T cell lineages.^439^ Different from TCF-1^+^ CD8^+^ Tpex cells, TCF-1 expression in chronic virus-specific CD4^+^ T cells does not adequately define stem-like progenitor CD4^+^ T cells, rather marks and promotes Tfh cell development.^431^ Recently, Xia et al. identified a population of memory-like TCF-1^+^Bcl-6^lo/−^ virus-specific CD4^+^ T cells emerged as the progenitor cells that gives rise to Teff and Tfh cells, sustaining CD4^+^ T cell response in chronic infection.^441^ Importantly, such CD4^+^ progenitor cells play pivotal roles in anti-tumor response preferentially at site of TdLNs.^441^ Hence, CD4^+^ T cells display exhausted yet functional phenotype in chronic infection.

### CD4^+^ Th cell subsets

#### Th1 and Th2

Th1 cells predominantly exert the anti-tumor activity. The frequency of Th1 subset and IFN-γ production in TME correlate positively with better clinical outcomes in multiple tumor types including melanoma,^442^ breast,^443,444^ ovarian,^445^ lung,^446^ colorectal,^447^ and laryngeal cancers^448^ (Table 2). Th1 cells promote tumor rejection by shaping an anti-tumor immune environment and indirectly supporting effector functions of other immune cells.^449,450^ Th1 cells are an important CD4^+^ T cell subset providing help for CD8^+^ T cell response and function,^451^ which will be elaborated at the later section. The migration of effector CD8^+^ T cells and NK cells in TME depends on chemokine receptor CXCR3 and its ligand CXCL9 and CXCL10 which are predominantly expressed by Th1-related IFN-γ-activated macrophages, cancer-associated fibroblasts (CAFs) and tumor cells.^452–454^ In addition, IFN-γ and IL-2 produced by Th1 cells enhance the survival, proliferation and cytolytic function of CD8^+^ CTLs and NK cells.^449,455^ IFN-γ can significantly enhance MHC I and MHC II expression and tumor-derived antigen presentation on tumor cells.^456,457^

**Table 2:** CD4^+^ T helper cell subsets in tumor immunity

Th subset	Phenotype	Tumor immunity	Tumor types	Functions	Refs
Th1	CXCR3, IFN-γ, TNF-α, IL-2, T-bet	anti-tumor	Melanoma, breast, ovarian, lung, colorectal and laryngeal cancers	activate macrophages, CAFs and tumor cells	^452–454^
				enhance MHC I and MHC II expression	^456,457^
				attract NK and CD8^+^ T cells	^452–454^
				support effector functions of NK and CD8^+^ T cells	^449,455^
Th2	IL-3, IL-4, IL-5, IL-13, GM-CSF	anti-tumor	Plasmacytoma, melanoma, myeloma, breast cancer	activate eosinophils and M2-type macrophages	^461–463^
				enhance NK cell cytotoxic activities	^464^
				induce cancer cell terminal differentiation	^465^
	IL-4, IL-10, TGF-β	pro-tumor	Pancreatic and breast cancer	promote breast cancer metastasis	^466^
				suppress Th1 response	^467,468^
Th17	IL-17A, IL-17B, IL-17F, IL-21, IL-22, IL-23	anti-tumor	Chronic lymphocytic leukemia, gastric adenocarcinoma, cervical adenocarcinoma ovarian, colorectal, lung and breast cancers	induce cancer cell apoptosis	^512^
				enhance recruitment of anti-tumor NK cells, DCs, neutrophils and macrophages	^513–516^
				attract effector CD4^+^ and CD8^+^ T cell infiltration	^474,514,517,518^
	IL-17A, IL-17D, IL-25/IL-17E	pro-tumor	Breast cancer, melanoma, bladder carcinoma, B cell acute lymphoblastic leukemia, colorectal, lung, prostate, liver, pancreatic and gastric cancers	stimulate tumor cell growth and inhibit apoptosis	^482–485^
				promote CSCs maintenance and activation	^486,487^
				enhance tumor invasion and metastasis	^488–490^
				promote angiogenesis	^491–493^
				promote MDSCs, TAMs and neutrophils	^494–500^
				constrain effector NK and CD8^+^ T cells	^501,502^
				induce terminal CD8^+^ Tex cell differentiation	^503^
				affect vascular endothelial cells and keratinocytes	^504–506^
Th9	IL-9, IL-21	anti-tumor	Melanoma, chronic lymphocytic leukemia, non-Hodgkins lymphoma, lung, breast and colorectal cancers	direct tumor cell killing by granzymes	^521,522^
				promote recruitment of DCs	^524,525^
				induce CD8^+^ CTL and NK cell responses	^98,522,523^
				elicit IFN-α/β production by monocytes	^526^
				induce mast cell activation	^521,527^
		pro-tumor	Hodgkin lymphoma, anaplastic large cell lymphoma, B and T cell lymphomas, CRC, HCC, lung, mammary, breast cancers	enhance tumor cell survival and migration	^532–536^
				induce EMT and metastatic spreading	^488^
				mediate immunosuppression of mast and Treg cells	^537^
Treg	IL-17, IFN-γ, TNF-α	anti-tumor	CRC, HNSCC, Hodgkin’s lymphoma, estrogen receptor-negative breast cancer, esophageal cancer, oral and oropharyngeal squamous cell carcinomas	suppress pro-tumor Th17 responses	^548^
				express pro-inflammatory cytokines	^549,550^
	CD25, ICOS, OX40, 4-1BB, GITR, PD-1, CTLA-4, Lag-3, Tim-3, TIGIT, CCR4, CCR8 IL-10, TGF-β, IL-35, IL-33, IL-37 Foxp3, FoxO1, STAT5, NFAT, T-bet, Helios, Nr4a, Foxp1	pro-tumor	HCC, melanoma, breast, lung, cervical, gastric, bladder, renal, endometrial and ovarian cancers	kill effector T cells, APCs and NK cells	^554,555^
				produce inhibitory cytokines	^556–558^
				express coinhibitory molecules	^539,559–561^
				suppress APCs function	^541,567^
				suppress NKT cell cytotoxic activity	^568^
				facilitate suppressive activity of MDSCs	^569,570^
				produce adenosine by CD73 and CD39	^571,572^
				compete IL-2 with effector T cells	^541,573^
				produce IDO	^574,575^
Tfh	CXCR5, PD-1, ICOS, Bcl-6 IL-4, CXCL13, IL-21	anti-tumor	Melanoma, breast, colorectal and lung cancers	promote the formation of TLSs	^479,597^
				induce pro-inflammatory cytokines	^132,598^
				activate complement cascade	^132,598^
				promote effective cytotoxic lymphocytes	^132,598^
				enhance CD8^+^ T cell response	^436,592,602^
				promote GC response and antibody production	^312,603^^,1109^
				support B cells and memory B cells	^606,607^
				respond to PD-1-based ICB	^590,608^

The role of Th2 cells in tumor progression remains controversial with both favorable and deleterious effects^458–460^ (Table 2). Previously, Th2 cells have been shown to suppress tumor growth by activating eosinophils as the cytotoxic effector cells in murine plasmacytoma and melanoma.^461,462^ Adoptive transfer of tumor-specific Th2 cells induced massive accumulation of M2-type macrophages at the tumor site, which triggered an inflammatory immune response to eliminate myeloma cells.^463^ Memory Th2 cells display potent anti-tumor activity by producing IL-4 to enhance NK cell cytotoxic activities.^464^ Moreover, Th2 cells can directly block breast carcinogenesis by secreting IL-3, IL-5, IL-13, and GM-CSF, which induce the terminal differentiation of the cancer cells.^465^ However, in pancreatic cancer, thymic stromal lymphopoietin (TSLP) produced by CAFs attracts and induces Th2 cells, which correlates with reduced patient survival.^459^ Th2 associated IL-4 signaling in monocytes and macrophages promotes breast cancer metastasis.^466^ Th2 cells can also attenuate Th1-associated anti-tumor responses through IL-4 signaling.^467,468^ In accordance with this notion, Th1-dominant immune response—upregulation of Th1-related response while downregulation of Th2-associated response—can be used as positive prognostic indicators for certain cancers.^469–471^ The discrepancy of Th2-mediated tumor immunity may attribute to different tumor types and distinct Th2 cell state. For example, studies have suggested that tumor-promoting Th2 cells have high levels of IL-10 and TGF-β, whereas Th2 cells with high expression of IL-3, IL-5, IL-13, and GM-CSF exhibit pro-inflammatory and anti-tumor immunity.^465,472^

#### Th17

Th17 cells are specifically accumulated in many types of human tumors.^473^ Cytokine milieu formed by IL-1β, IL-6, IL-23, and TGF-β produced by tumor cells, CAFs and tumor-associated macrophages (TAMs) supports Th17 cell differentiation and expansion.^474,475^ However, the effects of Th17 cells and cytokine IL-17 on tumor immunity are contradictory.^473,476^ Therefore, the presence of Th17 cells is associated with either good or poor prognosis depending on tumor types^477–479^ (Table 2). The pro-tumor function of Th17 cells is attributed to both direct effects on tumor cells and indirect effects of inducing a pro-inflammatory environment.^480,481^ Th17 cells and IL-17 strongly stimulate tumor cell proliferation by activating growth-related kinases and TFs, while inhibit their apoptosis by acting on anti-apoptotic proteins.^482–485^ Th17 cells and IL-17 promote cancer stem cells (CSCs) maintenance, pro-tumorigenesis and activation.^486,487^ Th17 cells also enhance tumor invasion and metastasis in lung, prostate, liver, and pancreatic cancers by inducing tumor cell epithelial-mesenchymal transition (EMT), matrix metalloproteinases (MMPs) expression, and chemokine expression.^488–490^ A key mechanism for the pro-tumor activity of Th17 cells is that IL-17 promotes angiogenesis.^491^ IL-17 in TME often correlates with high vascular density and tumor overgrowth, and induces the production of angiogenic factors such as vascular endothelial growth factor (VEGF), IL-6 and IL-8 by tumor cells or stromal cells.^492,493^ Furthermore, Th17 cells and IL-17 indirectly shape a pro-tumor TME by recruiting and influencing other immunosuppressive cells. For instance, IL-17 promotes the development, tumor infiltration and immunosuppressive activity of myeloid derived suppressor cells (MDSCs),^494,495^ TAMs,^496–498^ and pro-tumor neutrophils.^499,500^ IL-17 also constrains the cytolytic activity of NK cells and CD8^+^ T cells by inhibiting IL-15-mediated cell maturation^501^ and recruiting neutrophils,^502^ respectively. Interestingly, IL-17 also promotes tumor progression through inducing terminal exhausted CD8^+^ T cell differentiation.^503^ Apart from immune cells, IL-17 increases vascular endothelial cells number in gastric cancer,^504^ triggers CAFs to produce myeloid cell stimulatory factor G-CSF,^505^ and promotes skin tumor formation by stimulating keratinocyte proliferation.^506^ Furthermore, Th17 cells secrete high level of IL-22 which enhances the tumor growth and metastasis in human colon cancer.^507,508^

On the contrary, Th17 cells and IL-17 are found positively associated with better prognosis and improved patient survival in various cancers^474,509–511^ (Table 2), indicating a tumor-protective role of Th17 cells. The underlying mechanisms for the anti-tumor activity of Th17 cells also rely on direct and indirect functions. IL-17 acts on IL-17R-expressing tumor cells and induces caspase-dependent apoptosis signaling in breast cancer.^512^ IL-17 enhances the recruitment and anti-tumor functions of NK cells,^513^ DCs,^514^ neutrophils,^515^ and pro-inflammatory macrophages.^516^ Th17 cells stimulate CXCL9 and CXCL10 production from tumor cells to attract effector CD4^+^ and CD8^+^ T cell infiltration, and increase IFN-γ^+^ T cell activity.^474,514,517^ Furthermore, IL-17-producing CD4^+^ and CD8^+^ T cells display improved potency to repress tumor growth.^518^ The multifaceted and discrepant functions of Th17 cells in the context of tumor likely derive from distinct tumor types, and more importantly, high plasticity of Th17 cells which can be transdifferentiated into other Th lineages including Th1, Th2, Tfh, and T_reg_ cells, endowing them with discrete or opposing functions.^519^ Additionally, IL-17 is produced by many cell types besides Th17 cells, such as neutrophils, γδ T cells, macrophages, MDSCs, mast cells, endothelial cells, tumor cells and CAFs.^519^ Thus, it is important to distinguish the effects of Th17 cells and IL-17 on tumor immunity.

#### Th9

Th9 cells have been receiving much attention recently due to the fact that this CD4^+^ T cell subset and its featured cytokine IL-9 exhibit unprecedented anti-tumor immunity.^100,479^ High frequency of Th9 cells was found positively correlated with better prognosis in NSCLC patients.^520^ The potent anti-tumor activity of Th9 cells relies on both direct tumor cell killing and indirect roles in shaping anti-tumor immunity. Studies have shown that Th9 cells express high level of granzymes and display direct cytotoxic activity on melanoma cells.^521,522^ Th9 cells can induce robust CD8^+^ CTL and NK cell responses by secretion of cytokines IL-9 and IL-21.^98,522,523^ IL-9 may also enhance CD8^+^ T cell function through promoting recruitment of DCs into the tumor tissue^524^ and enhancing their antigen cross-presentation.^525^ Thus, administration of IL-9 neutralizing antibody inhibits tumor-specific CD8^+^ T cell responses and results in tumor progression.^524^ By increasing intratumor ATP, Th9 cells induce monocytes infiltration and production of IFN-α/β.^526^ Moreover, the anti-tumor activity of Th9 cells depends on mast cell activation.^521,527^ Notably, intratumoral Th9 cells are found less-exhausted and highly proliferative and cytolytic, and only Th9 cells could completely eradicate advanced tumors compared to other tumor-killing CD4^+^ T cell subsets such as Th1 and Th17 cells.^528^ Hence, Th9 cells represent an effective population of CD4^+^ T cells for adoptive cell therapy.^526,529,530^

Despite considerable evidence showing the potent anti-tumor activity of Th9 cells, pro-tumoral roles of Th9 cells have also been reported. Overexpression of IL-9 is detected in various cancers (Table 2), which is strongly associated with augmented tumorigenesis and shorter disease-free survival period.^92,531,532^ IL-9 can directly enhance tumor cell survival and migration through activation of JAK1 and JAK3, and STAT (STAT3 and STAT5) signaling pathways.^532–534^ In chronic lymphocytic leukemia (CLL) patients, an autocrine-positive feedback loop of Th9/IL-9 axis promotes malignant T cell survival.^535,536^ In addition, IL-9 promotes tumor progression by inducing EMT and metastatic spreading in lung cancers.^488^ IL-9 contributes to tumor growth by mediating immunosuppression of mast cells and T_reg_ cells.^537^ IL-9 in TME functions as an immunosuppressor for adaptive immunity in which IL-9 depletion or neutralization could restore the immunological memory for effective tumor rejection.^538^ Given those inconsistent results, further studies are needed to fully delineate the function of Th9 cells in tumors especially their clinical relevance in human.

#### T_reg_ cells

As a major immunosuppressive subset of CD4^+^ T cells, T_reg_ cells are found substantially infiltrated in many solid tumors.^539–541^ The high frequency of T_reg_ cells is mainly associated with worse clinical outcomes in majority of tumor types such as HCC, melanoma, breast, lung, cervical, gastric, bladder, renal, endometrial, and ovarian cancers.^542–544^ However, T_reg_ infiltration may also correlate with better prognosis in CRC, HNSCC, Hodgkin’s lymphoma, estrogen receptor-negative breast cancer, esophageal cancer, and oral and oropharyngeal squamous cell carcinomas.^543,545,546^ This discrepancy may be related to different TME, T_reg_ cell plasticity and their interplay with other cells. For instance, T_reg_ cells infiltrated in CRC are enriched for less immunosuppressive Foxp3^lo^ population rather than more immunosuppressive Foxp3^hi^ subset.^547^ Th17 cell-mediated pro-inflammatory and pro-tumor responses in CRC can be attenuated by T_reg_ cells.^548^ In addition, T_reg_ cells in CRC can also be induced to express pro-inflammatory cytokines including IL-17, IFN-γ, and TNF-α, exerting an anti-tumor immunity.^549,550^ Therefore, high T_reg_ cells together with a low frequency of CD8^+^ CTLs are better prediction for unfavorable prognosis in various types of cancer.^542,551^

Compared to T_reg_ cells in non-tumor tissues, intratumoral Foxp3^+^ T_reg_ cells are mostly active and highly proliferative,^552^ expressing elevated levels of activation markers CD25, ICOS, TNFR superfamily members OX40, 4-1BB, and GITR, various IRs, and chemokine receptors CCR4 and CCR8.^542,553^ Emerging evidence has revealed a variety of mechanisms contributing to T_reg_ cell immunosuppression: (1) T_reg_ cells can directly kill effector T cells, APCs and NK cells by expressing perforin and granzyme B, and induce cell apoptosis by FasL/Fas signaling.^554,555^ (2) T_reg_ cells mediate immunosuppression through producing inhibitory cytokines, including IL-10, TGF-β, IL-35, IL-33, and IL-37.^556–558^ (3) T_reg_ cells express a spectrum of high levels of coinhibitory molecules, such as CTLA-4, PD-1, Lag-3, Tim-3, and TIGIT.^539,559–561^ For instance, CTLA-4 competes with costimulatory receptor CD28 on effector T cells for binding to CD80/CD86 on APCs.^562^ CTLA-4 further downregulates CD80/CD86 expression via trans-endocytosis and trogocytosis.^563–565^ In addition, T_reg_ cells maintain memory CD8^+^ T cell quiescence by suppressing their effector and proliferative programs through CTLA-4 signaling.^566^ (4) T_reg_ cells exert immunoregulatory functions by influencing other immune cells. Engagement of CTLA-4 and Lag-3 on T_reg_ cells with CD80/CD86 and MHC II molecules on DCs respectively, results in suppression of antigen-presenting function and subsequent activation of effector T cells.^541,567^ In addition, T_reg_ cells suppress NKT cell cytotoxic activity in a cell-cell contact-dependent manner,^568^ while facilitate the immunosuppressive activity of MDSCs.^569,570^ (5) T_reg_ cells dampen the anti-tumor immunity by shaping an immunosuppressive TME involved in suppressive metabolites. High expression of ectonucleotidase CD39 and CD73 on T_reg_ cells can convert extracellular ATP or ADP into adenosine which induces broadly inhibitory signals in effector T cells, NK cells, and DCs.^571,572^ IL-2, as an essential cytokine for effector T cell activation and proliferation, is consumed by T_reg_ cells which express high level of CD25, the high-affinity IL-2Rα.^541,573^ T_reg_ cells also increase indoleamine 2, 3-dioxygenase (IDO) production which mediates tryptophan metabolism and causes effector T cell dysfunction.^574,575^

Another essential aspect regarding to tumor-infiltrating T_reg_ cells is their origin. Comprehensive transcriptomic and TCR repertoire analyses have revealed both nT_reg_ and iT_reg_ cells serve as the cell sources,^570,576,577^ and tumor-infiltrating T_reg_ cells are both recruited from the periphery or TdLNs, and expanded within the TME.^578,579^ A variety of chemokine receptors on T_reg_ cells and their cognate ligands are involved in the recruitment of T_reg_ cells, including CCR4 (CCL17 and CCL22), CCR8 (CCL1, CCL8, CCL16 and CCL18), CCR2 (CCL2), CCR5 (CCL5), CCR6 (CCL20), CCR10 (CCL28 and CCL27), CXCR3 (CXCL9, CXCL10 and CXCL11), and CXCR4 (CXCL12).^345,580,581^ Among distinct mechanisms, signals from tumor antigen stimulation, ICOS/ICOSL, TNFR2, 4-1BB, OX40, and GITR significantly drive T_reg_ cell expansion and functionality.^540,580,582^ In addition, the nutrient-deprived TME plays critical roles in reprogramming T_reg_ cell metabolism and activity.^583^ Glycolysis, fatty acid oxidation and oxidative phosphorylation are all important for the differentiation and function of tumor-infiltrating T_reg_ cells.^570,584,585^ Particularly, lactic acid uptake in T_reg_ cells promotes PD-1 expression which dampens the efficacy of anti-PD-1 immunotherapy,^586,587^ and uptake of free fatty acids and low-density lipoprotein via scavenger receptor CD36 is required for intratumoral T_reg_ cell survival, amplification and suppressive function.^583,588,589^

### Tfh and tertiary lymphoid structures (TLSs)

Tfh cells mainly support B cell responses and antibody production in infectious disease and vaccination.^131,132^ It is surprising to found a close link between Tfh cell response and anti-tumor immunity.^131,590^ Persistent antigenic stimulation during chronic viral infection and tumor redirects CD4^+^ T cell differentiation toward Tfh cells.^131,591,592^ Recent studies have revealed a positive correlation between the presence of Tfh and B cells with prolonged survival and better prognosis in a variety of human tumors, including melanoma,^593^ breast cancer,^594^ colorectal cancer,^595^ and lung cancers.^596^

The underlying mechanisms by which Tfh cells exert protective functions in infection and tumor are: (1) Tfh cell response significantly promotes the formation of TLSs which are ectopic tissue structures consisting B cells, T cells, NK cells and APCs in nonlymphoid organs under chronic inflammatory stimulation.^479,597^ Mature TLSs within tumors represent anti-tumor contextures with pro-inflammatory cytokines, activated complement cascade, and effective cytotoxic lymphocytes.^132,598^ Tumor-infiltrating Tfh cells expressing high levels of CXCL13 and IL-21 are enriched in intratumoral TLSs and strongly associated with infiltration of CD8^+^ T cells and B cells, as well as prolonged survival in cancer patients.^599–601^ (2) Tfh cells can enhance CD8^+^ T cell response in chronic viral infection and tumor through producing CXCL13 and IL-21,^436,592,602^ which will be further discussed at later section. (3) Tfh cells promote B cell and GC response and production of functional antibodies.^603^ Potent anti-tumor immunity requires antibody-mediated effector functions such as antibody-dependent cell cytotoxicity (ADCC), complement activation and antibody-mediated tumor cell phagocytosis.^604^ Tumors with high Tfh cells and mature TLSs mostly have high density and diversity of B cells and plasma cells, as well as tumor-targeting antibodies, which further induces effective anti-tumor immunity.^598,605^ (4) Tfh cells support the generation of memory B cells which are crucial for rapid response upon reinfection and long-term protection.^606,607^ (5) Tfh cells contribute to PD-1-based ICB.^590,608^ It is noteworthy that high PD-1 expression on Tfh cells does not indicate cell exhaustion, instead, promote Tfh cell expansion, activity and function.^609,610^ In clinical studies, the densities of Tfh cells, TLSs and tumor-infiltrating B cells positively correlate with the overall survival and responsiveness in patients treated with immunotherapy in various tumor types.^593,611,612^ The benefit of Tfh cells for anti-PD-1 therapy partially depends on their activity to recruit CD8^+^ T cells through CXCL13/CXCR5 signaling axis.^613,614^ Consistently, histological analysis confirms a spatial proximity of CXCL13^+^ Tfh cells, CXCR5^+^ CD8^+^ T cells and CD20^+^ B cells within TLSs, which enhances the efficacy of PD-1 ICB.^615^

## CD4^+^ T cell help enhances anti-tumor response of CD8^+^ CTLs

### Help mechanisms

**Fig. 6:**
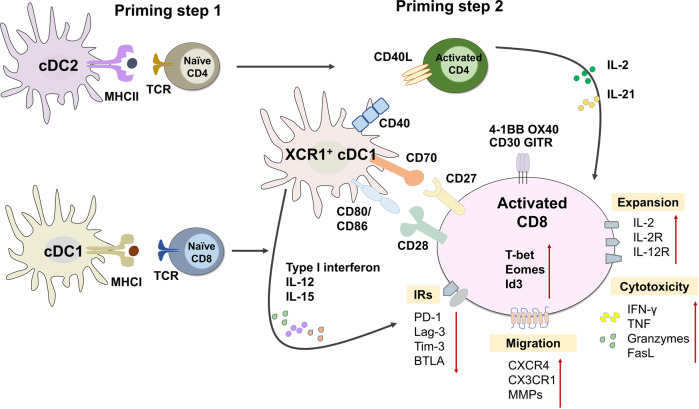
CD4^+^ T cells support CD8^+^ CTL response in anti-tumor immunity. Effective CD8^+^ CTL priming is a two-step process dependent on CD4^+^ T cell help which is bridged by XCR1^+^ resident cDC1s. CD4^+^ and CD8^+^ T cells are activated separately by different populations of DCs. Through CD40/CD40L signaling, activated CD4^+^ T cells enhance the expression of CD80/CD86 and CD70 on cDC1s, which interact with CD28 and CD27 on CD8^+^ T cells to promote their activation. CD4^+^ T cell-helped cDC1s also secrete high levels of type I interferon, IL-12 and IL-15 to promote CD8^+^ T cell survival and effector function. CD4^+^ T cells can directly promote CD8^+^ CTL response through IL-2 and IL-21. Consequently, CD4^+^ T cell-helped CD8^+^ T cells exhibit enhanced expansion, cytotoxic activity, migratory capacity, and expression of TNFR and key transcription factors, while downregulated IRs

Although CD8^+^ CTLs play the predominant roles in anti-tumor immunity, it is now well-appreciated that CD4^+^ T cells are pivotal to support the effective anti-tumor CD8^+^ T cell responses (Fig. 6). Growing evidence has indicated that a cooperation between CD4^+^ and CD8^+^ T cells within tumor milieu is required for effective tumor regression.^449^ By comparing the transcriptomic profiles of CTLs with or without CD4^+^ T cell help, it has been demonstrated that CD4^+^ T cells can help CTLs in multiple cellular processes, including priming, clonal expansion, effector function, memory formation and response to cancer immunotherapies.^616,617^ Full CD8^+^ T cell priming is a two-step process in which CD4^+^ T cells and CD8^+^ T cells first encounter antigens separately on different types of cDCs (cDC2 and cDC1 respectively) that may occur at different location of the second lymphoid tissues.^617–619^ In the second priming step, CD4^+^ T cells and CD8^+^ T cells recognize their antigen on the same DCs (mainly XCR1^+^ resident cDC1s).^620–622^ CD4^+^ T cells enhance DC activation and their antigen-presenting capability via CD40/CD40L signaling to fully prime CTL response.^623,624^ Therefore, eliciting CD4^+^ T cell response or pre-stimulating DCs with CD40 agonist are essential strategies for effective anti-tumor vaccines.^625,626^

CD4^+^ T cell help also promotes the clonal expansion and effector function of CTLs. Helped CTLs have upregulated expression of IL-2, IL-2R and IL-12R to support their survival, proliferation and effector differentiation.^308,627^ Helped CTLs exhibit enhanced cytotoxic activities, including increased production of IFN-γ, TNF, granzymes and Fas ligand, while downregulated IRs such as PD-1, Lag-3, Tim-3, and BTLA.^301,628^ On the contrary, helpless CTLs display dysfunctional and exhausted phenotypes.^629,630^ Furthermore, CD4^+^ T cells help CTL migratory capacity to enter tumor tissues by upregulating their CXCR4 and CX3CR1 expression, and promote CTL extravasation at tumor site by increasing MMPs expression.^628^ More importantly, CD4^+^ T cell help is required for generating long-term memory CD8^+^ T cells.^302,304,631^ CD4^+^ T cell help promotes IL-15 signaling for T_CM_ maintenance, as well as IFN-γ and granzyme B production from T_EM_.^632^ In the absence of CD4^+^ T cell help, memory CTLs exhibit reduced CD27 expression and IL-2 production,^633^ and impaired recall response likely due to massive cell apoptosis, which are associated with increased expression of the death ligand TRAIL and decreased expression of anti-apoptotic protein Bcl-2.^634,635^ Mechanistically, CD4^+^ T cells enhance the expression of key TFs for effector and memory CTLs, such as T-bet, Eomes and Id3.^617,636^

### Help signals

The help from CD4^+^ T cells mostly depends on costimulatory and cytokine signals (Fig. 6). In the second step of CTL priming, CD4^+^ T cell help triggers upregulation of CD80/CD86 and CD70 on cDC1s, which interact with CD28 and CD27 on CD8^+^ T cells, respectively.^637,638^ CD28 costimulation is important but not sufficient to generate fully functional CTL response.^639,640^ Costimulation through CD70/CD27 is critical for CD8^+^ CTL priming, clonal expansion and differentiation into both effector and memory CTLs.^641,642^ Besides, other TNFR family members such as 4-1BB, OX40, CD30, and GITR may also play critical roles in mediating CD4^+^ T cell help.^643,644^ CD4^+^ T cell-helped cDC1s have increased expression of type I interferon, IL-12 and IL-15 to promote effector CD8^+^ T cell survival, differentiation and function.^635,645^ CD4^+^ T cell help augments IL-2Rα expression on primed CD8^+^ T cells, together with IL-2 produced by CD4^+^ T cells, contributing to CTL clonal expansion, effector differentiation and function.^308,646^ In addition, CD4^+^ T cell-derived IL-21 is required for CX3CR1-expressing CD8^+^ T cell differentiation and cytolytic function,^360,602,647^ promotes TCF-1^+^ stem-like CD8^+^ T cell generation and maintenance and prevents effector CD8^+^ T cell exhaustion.^648,649^ Tfh cells expressing CXCL13 attract CXCR5^+^ CD8^+^ T cell migration in chronic infection and cancer.^650,651^ Collectively, costimulatory and cytokine signals from CD4^+^ T cells collaboratively and non-redundantly support CD8^+^ CTL response.

## T cells in autoimmune diseases

A healthy immune system is a functional network important for host homeostasis by protecting from infection while preventing self-reactivity. Disruption of this delicate immune balance causes autoimmune diseases. To date, more than 80 types of autoimmune diseases have been described, affecting approximately 5–8% of the world population.^652^ The autoimmune diseases can be systemic, such as systemic lupus erythematosus (SLE) and rheumatoid arthritis (RA), or organ specific, such as multiple sclerosis (MS) and Type 1 Diabetes (T1D). Although the mechanisms underlying autoimmune disorders are complicated and poorly understood, the roles of autoreactive T cells in driving the immunopathogenesis have been characterized in various autoimmune disorders (Table 3).

**Table 3:** CD4^+^ T helper cell subsets in autoimmune diseases

Th subset	Mediator	Pathogenesis	Autoimmune disease	Functions	Refs
Th1	IFN-γ	promote	MS	activate pro-inflammatory M1-like microglia	^654,655,657,673,674^
Th17	IL-17A-F, IL-21, IL-22, IFN-γ, GM-CSF	promote	MS	activate macrophages, astrocytes, epithelial and endothelial cells and oligodendrocytes	^102,500,668,669,671–674^
				recruit neutrophils	^102,500,668^
				disrupt BBB	^667^
				support formation of TLOs	^663,675^
				promote pathogenic myeloid cells	^683^
	IL-17, IL-1β, TNFα, GM-CSF	promote	RA	induce tissue-destructive enzymes, pannus growth, osteoclastogenesis and angiogenesis	^690–693^
				enhance proliferation of fibroblast-like synoviocytes	^694^
				stimulate GM-CSF secretion from fibroblast-like synoviocytes and ILCs	^695^
	IL-17A, IL-17F, IL-21, IL-22, IL-23	promote	SLE	stimulate keratinocytes, synoviocytes, fibroblasts, macrophages and neutrophils	^704^
				induce the NETosis	^705^
Th1-like Th17	IFN-γ, IL-17, granzymes, GM-CSF, IL-22	promote	MS	produce inflammatory cytokines	^707–709^
				cross BBB	^711,712^
				promotes the neuroinflammation	^711–713^
Th22	IL-22, IFN-γ, TNF-α, IL-17	promote	MS, SLE, RA, psoriasis, ITP, AIH, AITD, MG, SSc	disrupt BBB	^667^
				affect endothelial cells	^667^
				regulate astrocytes, oligodendrocytes, T_reg_ cells	^716,727^
				contribute to bone destruction	^731,732^
				promote fibroblasts proliferation and inflammatory responses	^731,732^
				induce osteoclast formation	^731,733^
Th9	IL-9	promote	IBD, SLE, MS, SSc, UC, RA, psoriasis, IRP, thrombocytopenia	suppress epithelial cell proliferation	^736^
				disrupt mucosal barrier function	^93^
				promote Th17 cell migration and differentiation	^739,746^
				induce astrocytes response	^745,747^
				promote B cell proliferation and autoantibodies production	^749,750^
				enhance MMPs production by neutrophils	^738,754^
		prevent	Gastritis, MS	dampen the pathogenic activity of Th17 cells	^755^
				interfere with IL-17 and Th17 cell polarization	^756^
				maintain T_reg_ differentiation	^757^
Tfh	CD40L, IL-4, IL-21, CXCL13	promote	MS, RA, SLE, MG, Sjögren’s syndrome, psoriasis, AD, autoimmune thyroid, hepatitis disease, IBD and T1D	drive autoreactive B cell response and autoantibody development	^769–773,793,794^
				promote the inflammatory Th17 responses	^778^
				induce pathogenic CD8^+^ T cell responses	^784,795^
				promote osteoclasts, fibroblast-like synoviocytes, keratinocytes and synovial macrophages	^797–801^
				counteract Treg cell suppressive activity	^802,803^
				help pathogenic epitope spreading	^817,819,820^
T_reg_	CTLA-4, Lag-3, TIGIT, CD73, CD39, IL-10, TGF-β, IL-35	prevent	MS, asthma, T1D, MG, RA, SLE	prevent Tconv overactivation differentiate into Th-like T_reg_ cells to suppress Th cells	^832,833^
					^835–843^
	IFN-γ	promote	T1D, MS, autoimmune hepatitis, Sjögren’s syndrome	pro-inflammatory T_reg_: IFN-γ^+^Foxp3^+^ Th1-like T_reg_ cells	^853–859^
	IL-4, IL-13	promote	SSc, allergy, asthma, TAK, IOI	pro-inflammatory T_reg_: Foxp3^+^ Th2-like T_reg_ cells	^865–871^
	IL-17	promote	RA, SLE, psoriasis, mucosal autoimmunity, glomerulonephritis	pro-inflammatory T_reg_: IL-17^+^Foxp3^+^ Th17-like T_reg_ cells	^841,872–875^
	pro-inflammatory cytokines	promote	Diabetes, MG, MS, RA, SLE	instability of T_reg_ lineage: exFoxp3 cells	^883–889^
				impaired immunosuppressive function	
CD8	IFN-γ, TNF, granzyme B, perforin	promote	T1D, MS, vitiligo, Crohn disease, SLE, vasculitis, IBD	disrupt self-tissues by cytotoxic effector molecules	^919,922,923^
				enhance ROS production from monocytes	^919^
				presence of progenitor autoreactive T cells	^931^

### Th1, Th17, and Th1-like Th17 cells: important inflammation mediators

As a major pro-inflammatory CD4^+^ T cell subset, Th1 cells play critical roles in promoting pathogenesis of autoimmune diseases. MS is a chronic autoimmune disease characterized by immune dysfunction and inflammation in the central nervous system (CNS) where the immune cell infiltration triggers demyelination, axonal damage, and neurodegeneration.^653^ Experimental autoimmune encephalomyelitis (EAE) is the most used experimental model for MS. Th1 cells are found to be the most frequent CD4^+^ Th cells in the CNS of EAE and large amount of IFN-γ is detected in MS patients.^654,655^ Adoptive transfer of Th1 cells is sufficient to induce EAE manifestation in mouse models.^656^ The neuropathological roles of Th1 cells in the CNS are associated with microglia, the CNS-resident macrophages. Th1-associated factors could activate a pro-inflammatory M1-like microglia differentiation,^657^ and promote inflammation in EAE.^657^ However, later studies using IL-12p35 subunit, IL-12Rβ2 chain or IFN-γ deficient mice demonstrated that Th1 cells are not required in the pathogenesis of EAE and MS.^658,659^ Instead, loss of IL-23p19 subunit or IL-23R chain result in resistance to EAE.^660^ With the discovery of shared subunits between IL-23/IL-23R and IL-12/IL-12R, Th17 cells have been uncovered playing critical roles in autoimmune diseases.^661–663^ Th17 cells produce a variety of pro-inflammatory cytokines, such as IL-17A-F, IL-21 and IL-22, and pathogenic Th17 cells induced by IL-6, IL-1β, and IL-23 produce high levels of IFN-γ and GM-CSF,^115^ which can further act on several other cell types to amplify the inflammatory responses.

Th17 cells and IL-17 are highly involved in the pathogenesis of MS.^664^ In MS patients, IL-17-producing CD4^+^ T cells are largely found in the peripheral blood and cerebrospinal fluid.^665,666^ IL-17A infuses into the CNS and contributes to the disruption of blood–brain barrier (BBB).^667^ Pro-inflammatory cytokines produced by Th17 cells act on CNS-resident macrophages to enhance their activation, inflammatory cytokines and chemokines production, antigen-presenting activity, and recruit neutrophils into the inflammatory sites, thus promoting the axonal damage and neuroinflammation in EAE.^102,500,668^ Th17 cells, in cooperation with Th1 cells, affect astrocytes function by upregulation of inflammatory cytokines and chemokines while downregulation of neurotrophic factors.^669^ Therefore, inhibition of IL-17 signaling in astrocytes has been shown to ameliorate the EAE.^670^ IL-17 signaling also alters the expression of adhesion molecules on endothelial cells and actin cytoskeleton on epithelial barriers.^671,672^ In addition, Th17 cell- or IL-17-mediated pro-inflammatory responses inhibit the survival and maturation of oligodendrocytes whose apoptosis and dysfunction are highly associated with the demyelination and neurodegeneration in MS.^673,674^ Like TLSs in TME, tertiary lymphoid organs (TLOs) are observed in the chronically inflamed tissues in autoimmune diseases to sustain the local immune activation.^663,675^ IL-17 is required for the formation of TLOs by inducing CXCL13 and CCL19 production to recruit lymphocytes into TLOs.^676,677^ Furthermore, Th17 cell-derived GM-CSF has been identified as a key factor driving the inflammation during EAE development.^678,679^ It has been discovered that some CNS-infiltrated Th cells were IL-17A^+^GM-CSF^+^,^680^ and GM-CSF-producing T cells are increased in the peripheral blood and brain lesion.^681,682^ GM-CSF in turn enhances pathologic Th17 generation and maintenance,^680^ and acts on a variety of pathogenic myeloid cell types including inflammatory monocytes, monocyte-derived dendritic cells and microglia to promote EAE pathogenesis.^683^

RA is an autoimmune disorder characterized by the chronic inflammation in the synovial membrane. In autoimmune arthritis, Th17 cells are the dominant initiators and executors of inflammation. Increased level of IL-17 has been found in serum, synovial fluid and synovial tissue of patients with rheumatoid arthritis.^684,685^ Th17 activity and IL-17 correlate with the disease severity of clinical symptoms.^686,687^ Self-reactive T cells become activated and differentiated into CCR6^+^ Th17 cells in the periphery. Response to CCL20 expressed by synoviocytes, CCR6^+^ Th17 cells migrate to the joints to initiate inflammation by producing large amount of IL-17, IL-1β and TNFα.^688,689^ IL-17 contributes to the joint destruction by inducing tissue-destructive enzymes, pannus growth, osteoclastogenesis and angiogenesis.^690–693^ IL-17 enhances the proliferation of fibroblast-like synoviocytes through mTOR and MAPK p38 signaling.^694^ In addition, GM-CSF, produced directly by Th17 cells and Th17 cell-stimulated fibroblast-like synoviocytes and ILCs, is abundant in RA synovium and mediates chronic joint inflammation.^695^

SLE is a chronic and heterogeneous autoimmune disease featured by accumulation of autoantibodies and immune dysfunctions with systemic inflammation and tissue destruction in multiple organs such as skin, joint, kidney, brain, heart and blood.^696,697^ Emerging evidence has demonstrated that Th17 cells and IL-17 play essential roles in SLE pathogenesis.^698,699^ IL-17-producing T cells are increased in the peripheral blood and inflamed organs of SLE patients,^700,701^ and the IL-17 level positively correlates with the disease severity.^702,703^ IL-17A stimulates inflammatory cytokines and chemokines production by keratinocytes, synoviocytes, fibroblasts, macrophages and neutrophils.^704^ IL-17 also induces neutrophil extracellular trap formation (NETosis) which has been found promoting the pathogenesis of SLE.^705^ In addition, IL-23, a key cytokine for Th17 differentiation, is observed elevated in SLE patients and correlates with severe renal disease.^703,706^

Intriguingly, Th17 cells are highly plastic and can transdifferentiate into pathogenic Th1-like Th17 cells which are defined by producing high levels of both IFN-γ and IL-17, and co-expressing chemokine receptors CXCR3 and CCR6, as well as TFs T-bet and RORγt.^707^ Th1-like Th17 cells display stronger pathogenicity than Th17 cells, which may relate to the production of inflammatory cytokines GM-CSF and IL-22 and chemokine receptors CCR4, CCR6 and CXCR3.^708,709^ In inflammatory arthritis, both Th17 and Th1 lineage-specific TFs are highly expressed in the inflamed joints of patients. The cytokine milieu within the joints, including high levels of IL-12 but low IL-23 and TGF-β, converts Th17 cells into Th1-like cells. The direct evidence supporting the Th17 origin of Th1 cells results from the shared TCR clonality between Th1-like cells and Th17 cells.^710^ Th1-like Th17 cells are capable of crossing BBB and accumulate in the CNS where they promote the neuroinflammation in EAE mice and MS patients.^711–713^ Moreover, a CCR6^+^CXCR6^+^ cytotoxic Th17 population with expression of granzymes, IFN-γ and GM-CSF is identified to promote EAE pathology.^714^ Interestingly, a stem-like Th17 population is discovered by combined scRNA-seq and TCR-sequencing analysis and characterized by TCF-1 and SLAMF6 expression.^715^ Such Th17 progenitor cells are non-pathogenic but can give rise to GM-CSF^+^ and IFN-γ^+^ pathogenic Th17 populations under induction of IL-23, which greatly contributes to autoimmunity.^715^

### Th22: inflammation promotors

Th22 cells and IL-22 play critical roles in promoting autoimmune diseases. The proportion of Th22 cells and IL-22 level have been found increased in the serum and/or local tissues in numerous autoimmune disorders, including MS,^716^ SLE,^717^ RA,^718^ psoriasis,^719^ ITP,^720^ autoimmune hepatitis (AIH),^721^ autoimmune thyroid diseases (AITD),^722^ myasthenia gravis (MG),^723^ and systemic sclerosis (SSc).^724^ The IL-22 level is dynamically changed along with the disease progression.^725^ High CCR6 expression facilitates Th22 cell migration into the CNS.^726^ IL-22R expression was upregulated in the brain tissues of MS patients and IL-22 synergized with IL-17 to disrupt BBB tight junctions by affecting endothelial cells.^667^ IL-22 also regulates the survival and function of astrocytes and oligodendrocytes, and inhibits Foxp3 expression in T_reg_ cells, therefore promotes the pathogenesis of MS.^716,727^ In SLE, Th22 cells may represent a better prognostic marker of tissue involvement than Th17 cells.^728^ CCR6^+^ Th22 cells and IL-22 are increased in SLE patients with lupus skin diseases and significantly correlate with the SLE disease activity index (SLEDAI).^729,730^ The IL-22 level is also increased in the serum and kidney in patients with lupus nephritis, and treatment with anti-IL-22 monoclonal antibody could markedly reduce renal injury and inflammatory cells infiltration.^717^ In RA, Th22 cells positively correlate with disease activity score.^718,729^ High level of IL-22 in synovial tissue contributes to bone destruction and promotes fibroblasts proliferation and inflammatory responses.^731,732^ IL-22 also induces osteoclast formation through MAPK p38/NF-κB and JAK2/STAT3 signaling.^731,733^ Given the important function of Th22/IL-22 in promoting pathogenesis in many autoimmune diseases, targeting Th22/IL-22 has been considered as great therapeutic potentials.^734^

### Th9: dual-function in autoimmune diseases

Th9 cells and IL-9 have been implicated to play pathological roles in autoimmune diseases.^91^ IL-9, Th9 cells and Th9 cell-associated molecular features (PU.1, IL-4, TGF-β, etc.) have been found elevated in patients with various autoimmune diseases in ulcerative colitis (UC),^735^ inflammatory bowel disease (IBD),^736^ SLE,^737^ RA,^738^ psoriasis,^739^ immune-related pancytopenia (IRP),^740^ and thrombocytopenia,^741^ which greatly correlates with disease severity. In IBD, Th9 cells contribute to the pathogenesis through producing IL-9 which suppresses epithelial cell proliferation and disrupts the mucosal barrier function.^93,736^ In MS/EAE, Th9 cells and IL-9 function in initiating disease development and promoting inflammation in CNS. Adoptive transfer of myelin oligodendrocyte glycoprotein (MOG)-specific Th9 cells into Rag1^−/−^ mice sufficiently induces EAE more severe than transferring Th1 cells.^742,743^ IL-9 deficiency or neutralization exhibit attenuated EAE progression with reduced infiltration of Th17 cells and pro-inflammatory macrophages in the CNS, as well as decreased IL-17 and IFN-γ levels.^744,745^ Strikingly, cooperative functions of Th9 and Th17 cells have been revealed during autoimmune disorders. Th17 cells can produce IL-9 which acts as the pathogenic mediator in MS and psoriasis in animal models.^739,746^ In turn, IL-9 induces astrocytes to produce CCL20 which promotes Th17 cell migration into CNS and aggravates EAE development.^745,747^ Furthermore, the frequency of Th9 cells and serum IL-9 are positively associated with SLE disease severity.^748^ In murine lupus models, IL-9 is associated with increased anti-double-stranded DNA (dsDNA) antibodies via promoting B cell proliferation and autoantibody production.^749,750^ The enriched Th9 cell response in SLE is associated with NO^751^ which is elevated in SLE patients and enhances Th9 cell differentiation through TGF-β and IL-4 signaling^752^ and mTOR-HIF1α pathway.^753^ In RA patients, IL-9 and IL-9R are highly expressed in the synovial tissues, associated with synovial inflammatory infiltrates and the degree of ectopic lymphoid structures.^738^ Mechanistically, synovial IL-9 promotes the survival and MMPs production of neutrophils and facilitates Th17 cell differentiation.^754^

On the other hand, due to the complex immune microenvironment and regulatory mechanisms of autoimmune diseases, protective roles of Th9 cells are also observed. For instance, IL-9 dampens the pathogenic activity of Th17 cells in autoimmune gastritis.^755^ IL-9 inversely correlates with the inflammation and neurodegeneration in MS patients as high level of IL-9 interferes with IL-17 production and Th17 cell polarization.^756^ IL-9R deficient mice have increased Th1 and Th17 cell development but impaired T_reg_ cell activity, which is attributed to the important role of IL-9 in modulating Th17 and T_reg_ cell differentiation.^757^ Collectively, IL-9 and Th9 cells have both deleterious and protective roles in autoimmune diseases, and future comprehensive studies are required to fully delineate their functions.^748^

### Tfh: enhance autoreactive B cell and CD8^+^ T cell responses

Tfh cells are strongly associated with a wide range of autoimmune diseases in both autoantibody-dependent and -independent conditions. The first evidence of dysfunctional Tfh cells promoting autoimmunity comes from a study in 2005, in which Vinuesa et al. demonstrated that *Roquin* gene mutation caused excessive Tfh cell differentiation and systemic autoimmunity in mice.^758^ Deficiency of SAP, an adapter protein required for Tfh cell–B cell interactions,^759^ ameliorates the autoimmune phenotype with reduced autoantibody and disease severity.^760^ Increased frequencies of circulating Tfh cells are observed in majority of autoimmune disorders, including MS, RA, SLE, MG, Sjögren’s syndrome, psoriasis, atopic dermatitis (AD), autoimmune thyroid and hepatitis disease, IBD, and T1D.^761–763^ SLE is a well-known autoantibody-mediated autoimmune disease.^761,764^ Activated Tfh cells, aberrant GC responses and high level of autoantibodies are frequently found in SLE murine models^765,766^ and in lupus nephritis patients.^767,768^ The autoreactive B cells in SLE patients are typically somatically mutated and the anti-dsDNA antibodies have experienced somatic hypermutation and affinity maturation, indicating that they have been “helped” by T/Tfh cells.^769^ Similarly, the pathological progression in RA is strongly associated with autoantibodies which are mainly Tfh cell-helped high-affinity IgG antibodies.^770,771^ Tfh cells are expanded in patients with active RA, which positively correlates with autoantibody titers and disease severity.^772,773^ In RA joints, CXCL13-expressing Tfh cells co-localize with B cells and provide their help, which further promotes ectopic lymphoid structure formation and RA pathogenesis.^774,775^ Hence, the decreased percentage of Tfh cells has been used as an indicative biomarker for effectiveness of autoimmune disease treatments.^776,777^ In mouse EAE models, CXCR5^+^PD1^+^ Tfh cells are substantially infiltrated in the CNS tissue and promote the inflammatory B cell and Th17 cell responses, contributing to the disease pathogenesis.^778^ Furthermore, activated-memory circulating Tfh cells (CCR7^+^ICOS^+^) are increased in patients with relapsing MS, positively correlate with the levels of autoantibodies and disease severity, but are decreased after therapeutic treatment.^779^ Of note, while the pathogenic autoantibodies are predominantly derived from GC response and helped by GC-Tfh cells,^762,780^ Tfh cells can also support extrafollicular responses and autoantibodies production.^781,782^ T1D is an autoantibody less-dependent autoimmune disease in which overexpression of Tfh cell-related genes such as CXCR5, ICOS, PD-1, Bcl-6, and IL-21 are also observed.^783,784^ T1D can be induced by transferring Tfh cells in a mouse model.^783^Tfh cells positively correlate with the blood glucose levels and multiple autoantibodies in T1D patients.^785^ The frequency of activated autoantigen-specific Tfh cells (CXCR5^+^PD-1^+^ICOS^+^) is increased in both patients with recently diagnosed T1D or at risk of T1D.^786,787^

The pathogenic activity of Tfh cells largely depends on the signature cytokine IL-21 which promotes autoimmunity through helping B cells and driving effector function of CD8^+^ T cells as well as other cell types.^788,789^ IL-21 polymorphisms and overexpression are highly associated with autoantibodies, disease pathogenesis and clinical activity in many autoimmune disorders.^788,790–792^ IL-21 signaling strongly drives GC response, B cell activation, plasma cell differentiation and memory B cell formation, somatic hypermutation, and antibody class switching.^793,794^ In addition, IL-21R is highly expressed in CD8^+^ T cells and IL-21 signaling induces pathogenic CD8^+^ T cell responses. In T1D where the destruction of pancreatic β cells is primarily mediated by CD8^+^ T cells, IL-21-producing Tfh cells are increased significantly^784^ and IL-21R expression is elevated in CD8^+^ T cells.^795^ While IL-21 overexpression drives T1D development,^795^ IL-21R deficiency inhibits T1D mellitus.^796^ The functions of autoreactive CD8^+^ T cell responses in autoimmunity will be discussed in later chapter. Moreover, IL-21 can promote inflammation and pathogenesis by acting on other cells, such as osteoclasts,^797^ fibroblast-like synoviocytes,^798,799^ keratinocytes^800^ and synovial macrophages.^801^ In addition, Tfh cells counteract the suppressive activity of T_reg_ cells in autoimmune diseases through IL-21.^802,803^ Therefore, inhibition of Tfh cells and IL-21 signaling offers effective therapeutic strategies in autoimmune diseases.^804–806^ For example, treatment with steroids, immunosuppressive drugs or low-dose of IL-2, a potent inhibitor of Tfh cell differentiation,^149^ could significantly reduce the number of activated Tfh cells and result in improved clinical outcomes.^807–809^

Notably, many autoimmune diseases are likely triggered by infections due to pathogenic antigen mimics.^810,811^ For example, enteroviral infection has a strong association with T1D^812,813^; exposure to *Aggregatibacter actinomycetemcomitans* triggers the autoimmunity in RA^814^; EBV infection has a clear link with MS development^815,816^; autoantibodies in SLE are likely generated from response to commensal and/or environmental microbes^817^; patients infected with SARS-CoV-2 exhibit markedly increased autoantibodies.^818^ The underlying mechanisms are highly involved in Tfh cell-helped epitope spreading during infections. Specifically, self-reactive T cells cross-recognize microbial antigens and provide help to B cells bearing different specificities (bystander autoimmune B cells).^817,819^ For instance, influenza virus haemagglutinin-specific Tfh cells can help self-antigen MOG-specific B cells to produce autoantibodies when those B cells cocapture haemagglutinin and MOG.^820^ Collectively, Tfh cells potently drive the pathogenesis of autoimmune diseases through enhancing autoreactive B cell and CD8^+^ T cell responses.

### T_reg_ cells: critical autoimmune protectors

Autoimmune diseases are characterized as a failure of self-tolerance. As one of the most important T cell populations in maintaining immunological self-tolerance and homeostasis, T_reg_ cells play indispensable roles in autoimmunity.^821,822^ Mutations in *Foxp3* gene cause immunodysregulation polyendocrinopathy enteropathy X-linked syndrome (IPEX) which is a rare chromosome X-linked immunodeficiency syndrome with severe autoimmune disorders.^823,824^ Furthermore, mutations of T_reg_ cell-related signature genes, such as CD25,^825^ CTLA-4,^826,827^ LRBA,^828^ and AIRE,^829,830^ result in T_reg_ cell abnormality and severe autoimmune disorders. Depletion of Foxp3^+^ T_reg_ cells indeed leads to severe autoimmunity and immunopathology which can be rescued by reconstituting T_reg_ cells.^831^ By sensing IL-2 produced by autoreactive Tconv cells, T_reg_ cells co-localize with Tconv cells to prevent their overactivation.^832,833^ T_reg_ cells employ a variety of suppressive molecules for inhibitory functions, such as surface receptors CTLA-4, Lag-3, TIGIT, CD73, and CD39, and inhibitory cytokines IL-10, TGF-β, and IL-35.^822,834^ In addition, T_reg_ cells are able to adapt to the environment stimuli and mirror to corresponding effector Th cells under inflammatory conditions.^835^ T_reg_ cells can gain expression of signature TFs and chemokine receptors of Th1,^836,837^ Th2,^838,839^ Th17,^840,841^ and Tfh (known as T follicular regulatory (Tfr)) cells.^842,843^ By responding to different stimuli, these Th-like T_reg_ cells migrate into the same inflammatory sites with Th effector cells, and exert stronger abilities to suppress corresponding Th cell responses.^835^ The change of T_reg_ cell numbers in different autoimmune diseases has been largely studies, however, the results are strikingly inconsistent.^834,844^ The frequency of T_reg_ cells seems decreased in EAE^845^ and asthma,^846^ but unaffected in T1D^847^ and MG.^848^ Nevertheless, T_reg_ cell numbers are found either decreased,^849,850^ increased^845,851^ or unchanged in RA and SLE.^845,852^ Despite of inconsistence in cell number, it is well-acknowledged that the functions of T_reg_ cells in autoimmune milieu are compromised.^844^

Emerging evidence has suggested that the plasticity and instability of T_reg_ cells contribute to their dysfunction. While the Th-like T_reg_ cells exhibit advantages for controlling host homeostasis, aberrant plasticity can affect T_reg_ cell-mediated immunosuppression and exacerbate autoimmune diseases. It has been shown that the frequency of IFN-γ^+^Foxp3^+^ Th1-like T_reg_ cells are increased in various autoimmune diseases, such as T1D,^853^ MS,^854,855^ autoimmune hepatitis,^856^ and Sjögren’s syndrome.^857^ Th1-like T_reg_ cells accumulate at inflamed sites but fail to suppress effector T cell response and control the disease progression.^858,859^ Inflammatory cytokines TNF, IL-6, and IL-12,^860–862^ and PI3K-Akt-FoxO signaling pathway have been suggested to be involved in T_reg_ cell conversion.^855,863,864^ Th2-like T_reg_ cells are increased in patients with SSc,^865^ allergy,^866^ asthma,^867,868^ takayasu’s arteritis (TAK)^869^ and idiopathic orbital inflammation (IOI),^870^ and IL-33 derived from dermal fibroblasts contributes to Th2-like T_reg_ transdifferentiation.^871^ In addition, IL-17^+^Foxp3^+^ Th17-like T_reg_ cells are largely identified in RA,^872^ SLE,^873^ psoriasis,^874^ and mucosal autoimmunity,^841,875^ playing critical roles in disease pathogenesis. The conversion of T_reg_ cells into Th17 cells is driven by cytokines IL-1β, IL-6, IL-4, and IL-23,^862,872,876,877^ Toll-like receptor 2 (TLR2) stimulation,^878^ pathogenic infection^879^ and IRF4.^880^ In contrast, IL-33,^870^ SOCS1,^881^ and IDO^882^ have been suggested to prevent T_reg_ cell plasticity and restore their suppressive function.

Furthermore, under inflammatory or pathologic settings, instability of T_reg_ lineage with unstable Foxp3 expression and impaired immunosuppressive function is observed.^883,884^ Decreased Foxp3 expression is found in T_reg_ cells isolated from autoimmune diabetes,^885^ MG,^886,887^ MS,^888^ and SLE.^889^ T_reg_ cells loss of Foxp3 expression (exFoxp3) exhibit activated-memory T cell phenotype and acquire effector function, such as producing pro-inflammatory cytokines and inducing autoimmune pathogenesis.^890,891^ Under arthritic conditions, T_reg_ cells lose Foxp3 expression and transdifferentiate into Th17 cells (exFoxp3 Th17), which is driven by synovial fibroblast-derived IL-6. These exFoxp3 Th17 cells are more potent osteoclastogenic Th17 cells, contributing to the pathogenesis of RA.^872^ The mechanisms underlying T_reg_ cell stability have been greatly associated with the expression of master regulator Foxp3. Impairment of TGF-β/IL-2 signaling leads to diminished Foxp3 expression, T_reg_ cell function and autoimmune manifestations.^885,892–894^ Furthermore, the epigenic regulations of Foxp3 have been suggested playing both positive and negative roles in T_reg_ stability.^895^ Current consensus suggests that Foxp3 acetylation^896,897^ and O-linked *N*-acetylglucosamine (O-GlcNAc)^898^ stabilize its expression and strengthen T_reg_ stability and suppressive function, whereas methylation,^899,900^ phosphorylation^901,902^ and ubiquitination^903^ of Foxp3 induce instability of T_reg_ cells. The CNSs in Foxp3 locus are critical for Foxp3 transcription and are associated with autoimmune diseases.^904–906^ Methylation of T_reg_-specific demethylation region (TSDR)—a highly conserved CpG motif within CNS2—destabilizes Foxp3 expression and disrupts the suppressive activity of T_reg_ cells.^899,907^ Apart from epigenetic regulation, T_reg_ cell stability/suppressive function are profoundly controlled at transcriptional levels. Deficiency of TFs Helios,^908^ Ikzf4,^909^ RelA,^910^ Smad2/Smad3,^893^ AP-1^911^ and Id3^912^ significantly affects the stability of Foxp3 expression. In contrast, TFs BATF3,^913^ IRF4,^913^ E47,^912^ and Spi-B^912^ repress Foxp3 expression and T_reg_ cell induction.

### Autoreactive CD8^+^ T cells: new players in autoimmunity

Tradition views hold that CD8^+^ T cells mainly participate in protection against viral infections and tumors. However, increasing evidence from recent studies implicates that excessive CD8^+^ T cell functionality causes self-tissue damages and autoimmune disorders.^914,915^ In human, autoimmune disease susceptibility is highly associated with HLA class I (human MHC I) polymorphisms, prone to autoantigen presentation to CD8^+^ T cells.^916,917^ Autoreactive CD8^+^ T cells have been implicated in the pathogenesis of multiple autoimmune diseases, including T1D,^918^ MS,^919^ Crohn disease,^920^ and vitiligo.^921^ Pathogenic CD8^+^ T cells express high levels of cytotoxic effector molecules such as IFN-γ, TNF, granzyme B and perforin.^919,922,923^ In the nonobese diabetic (NOD) mouse model of T1D, by 10–15 weeks of age, the pancreata exhibit severe insulitis and are largely infiltrated with CD8^+^ T cells recognizing NRP-V7, a peptide from the diabetes antigen IGRP. The increased frequency of NRP-V7-reactive CD8^+^ T cells coincides with the time of glucose intolerance, suggesting that the progression of pancreatic islet inflammation is driven by self-reactive CD8^+^ T cell populations.^924,925^ In MS, autoreactive CD8^+^ T cells are expanded and enriched in the CNS of patients with relapsing–remitting disease.^926^ In EAE models, myelin basic protein (MBP)-specific CD8^+^ T cells are recruited to the CNS and enhance ROS production from monocytes in the brain lesion.^919^ In addition, CD8^+^ T cells contribute to autoimmune arthritis.^927^ The number of CD8^+^ T cells is increased in active RA patients but decreased in patients in remission.^928^ The elevated pro-inflammatory cytokine production by CD8^+^ T cells positively correlates with 28-joint disease activity score (DAS28) in autoimmune arthritis.^928^

Recent work has revealed a great heterogeneity of autoreactive CD8^+^ T cells. Pathogenic CD8^+^ T cells in T1D, MS/EAE and vitiligo contexts are predominant effector, effector memory or resident memory cells that initiate and promote disease progression.^919,922,929,930^ Even though autoreactive CD8^+^ T cells maintain effector functions, evidence also suggests that they display exhausted features. Autoimmune CD8^+^ T cells in MS and T1D have upregulated expression of IRs PD-1, Lag-3, and Tim-3.^919,931^ The exact function of exhausted CD8^+^ T cells in autoimmunity is not fully understood. However, some evidence has suggested a protective role of this population since T cell exhaustion represents a hyporesponsive phenotype. For instance, exhausted CD8^+^ T cells in T1D and SLE patients are associated with a slow disease progression and improved prognosis.^932,933^ Intriguingly, TCF-1^hi^TOX^hi^ stem-like progenitor CD8^+^ T cells have been identified in autoimmune diseases, which sustain the autoreactive T cell population.^931^ In T1D, this autoimmune progenitor CD8^+^ T cells are located at the pancreatic dLNs where they self-renew and give rise to autoimmune effector CD8^+^ T cells.^934^ Compared to the short-lived autoimmune effector cells, stem-like progenitors can induce T1D upon adoptive transfer of as few as 20 cells into recipient mice.^934^ Notably, the fate and functionality of self-reactive CD8^+^ T cells require TOX-dependent transcriptional and epigenetic reprogramming.^935^ Taken together, CD8^+^ T cells also function as autoimmune mediators, and further studies are required to better understand their cell heterogeneity, functional states and regulatory mechanisms in autoimmune diseases for developing effective therapeutic strategies.

## γδ T cells

γδ T cells are a unique and rare T cell population that are mainly enriched in peripheral mucosal barriers, such as skin, lung and gut tissues, playing critical roles in both maintaining physiological homeostasis and mediating immune responses in disease conditions. During intrathymic T cell development, DN3 cells rearrange the TCR components and those expressing TCR γ and δ chains develop into γδ T lineage (known as γδ-selection).^7^ It has been suggested that the γδ T cell fate relies on strong and prolonged TCR signal (instructive model),^7^ Id3 regulation,^936^ Sonic hedgehog (Shh) signaling,^937^ CD27 costimulation, cytokine IL-7, lymphotoxin (LT) signal from αβ thymocytes (known as *trans*-conditioning),^938^ and Notch signaling.^7^ Nevertheless, the requirement of Notch signal for γδ T cell differentiation is controversial and varies between mouse and human. Compared to αβ T cells, γδ-lineage commitment is less Notch dependent in mice^938^; however, γδ T cell development in human is highly dependent on NOTCH signaling.^939^ TCR signals through γδ-TCR complex not only promote the survival and maturation of pre-established γδ T cells,^7^ but also play an instructive role in γδ T-cell lineage commitment.^940^ In addition, more studies have revealed that γδ T cell development is orchestrated at transcriptional,^7^ epigenetic^941^ and metabolic levels.^942^

### γδ T cells in tissue surveillance and infection

Unlike αβ T cells that acquire effector function in the periphery, γδ T cells develop into effector cells during the development in the thymus. This early effector-programming of γδ T cells allows them to respond rapidly to pathogenic infections, inflammation, and tissue damage, endowing them with innate-cell like features. To date, two major subsets of effector γδ T cells are identified: IFN-γ producing Tγδ1 and IL-17 producing Tγδ17 cells, expressing key TFs T-bet and RORγt, respectively.^938^ Besides, γδ T effector cells can be distinguished by surface markers: Tγδ1 cells express CD27, CD122, NK1.1, and high level of CD45RB whereas Tγδ17 cells lack of the former three molecules but express CCR6, scavenger receptor SCART2 and low level of CD45RB.^943,944^ Distinct γδ T effector subpopulations have preferential Vγ usage and peripheral locations, such as IFN-γ producing cells are Vγ1^+^Vδ6.3^+^ (liver and spleen), Vγ5^+^ (skin), Vγ7^+^ (intestine), and IL-17 producing cells are mainly Vγ6^+^ (tongue, dermis, uterus, testis, adipose tissue, and brain) and Vγ4^+^ (lung, dermis, and lymph nodes).^945,946^ γδ T cell effector differentiation is regulated by transcriptional networks. In addition to T-bet and RORγt, TCF-1, LEF-1, Eomes, and Id3 are critical for IFN-γ producing γδ T cells, while c-Maf, Sox4, Sox13, HEB, Blk, and RelB are enriched for IL-17 producers.^947,948^ Of note, TCF-1 represses c-Maf/RORγt to limit Tγδ17 cells whereas c-Maf represses Tγδ1 fate by antagonizing TCF-1/LEF-1, indicating that an antagonism between c-Maf and TCF-1 controls the balance of these two γδ T effector subsets.^943^ Furthermore, γδ TCR signal strength impacts the effector fate, which TCR-Egr-Id3 pathway is required for IFN-γ production while TCR-E protein-TCF-1 axis supports IL-17-producing γδ T cell development.^936,949^ Thymic development of Tγδ1 cells requires Skint-1 signal from epithelial cells,^950^ while Tγδ17 cells can be differentiated in the periphery under IL-6, TGF-β, IL-1β, IL-18, and IL-23.^951,952^ With the advances in single-cell analysis, more insightful discoveries about the heterogeneity and developmental trajectory of tissue-specific γδ T cells have been further unveiled.^953^

Given the broad colonization in peripheral tissues, γδ T cells play crucial roles in tissue homeostasis and surveillance. γδ T cells sense “tissue status” by interaction with butyrophilins (BTNs) and BTN-like (BTNL) molecules which are members of the immunoglobulin superfamily.^954^ For example, BTNL1/BTNL6 heterodimers expressed on intestinal epithelial cells shape intestinal Vγ7^+^ T cells and BTNL3/BTNL8 heterodimers induce responses by colonic Vγ4^+^ T cells.^955^ γδ T cells promote wound healing and tissue repair in epithelial and mucosal barriers by producing functional factors and modulating other cells.^945^ In the skin, Vγ5^+^ dendritic epidermal T cells (DETCs) promote keratinocyte proliferation and hyaluronan production by producing keratinocyte growth factor (KGF) and insulin growth factor 1 (IGF1).^956,957^ Vγ7^+^ γδ T cells in intestines are highly associated with intestinal epithelial homeostasis through KGF1^958^ and IL-22.^959^ Gingival Vγ6^+^ T cells contribute to oral pathophysiology by producing IL-17 and amphiregulin.^960,961^ Notably, the function of Tγδ17 cells in tissue physiology can be paradoxical dependent on specific context. IL-17 producing Vγ4^+^ and Vγ6^+^ γδ T cells are found both contributing to the steady-state skin physiology^962^ as well as predominantly mediating the early inflammatory responses in skin diseases.^963^ Also, the roles of pulmonary γδ T cells can be beneficial, deleterious or dispensable in lung physiology and pathophysiology.^945^ Moreover, γδ T cells participate in non-barrier tissue surveillance. Vγ6^+^ Tγδ17 cells promote bone regeneration by stimulating the proliferation and osteoblast differentiation of mesenchymal progenitor cells.^964^ In the adipose tissue, γδ T cells, mainly Vγ6^+^ Tγδ17 subset, modulate T_reg_ cells and adipocytes through IL-17 and TNF to promote thermogenesis.^965,966^ Vγ6^+^ Tγδ17 cells also contribute to steady-state neurophysiology^967^ and initiation of neuroinflammation in EAE and brain injury.^676,963^

γδ T cells display both innate and adaptive immune cell characteristics by expressing gene rearranged γδ TCR with limited repertoire.^968^ γδ T cells can recognize unprocessed peptides and various non-peptide antigens, such as lipids and the phosphoantigens without MHC restriction.^969^ γδ T cells constitute the first line of host defense against pathogenic infections. During the skin infection with *S. aureus*, IL-17 producing Vγ4^+^ T cells and IFN-γ/TNF producing Vγ5^+^ T cells enhance neutrophil recruitment and bacterial clearance.^970,971^ Systemic *S. aureus* infection led to accumulation of IL-17A^+^ γδ T cells in the kidney for effective infection control.^972^ In the infected intestinal tract, Vγ7^+^ γδ T cells directly kill infected cells by secreting antimicrobial peptides and cytotoxic molecules.^973^ In Mtb infected lung tissue, Vγ4^+^ γδ T cells secrete CXCL2 and TNF to promote neutrophil recruitment and Vγ4^+^ and Vγ6^+^ Tγδ17 cells contribute to granuloma formation.^974,975^ Moreover, γδ T cells exhibit a potent antiviral activity against a variety of viruses.^976^ Upon recognition of viral antigens, γδ T cells become activated and express increased pro-inflammatory cytokines (IFN-γ and TNF-α) and cytotoxic molecules (perforin and granzymes) for pathogen clearance.^976^ During SARS-CoV2 infection, the frequency of γδ T cells is reduced in the circulation but increased in the airway tissues.^976^ Both circulating and tissue-colonized γδ T cells have upregulated activation phenotypes (CD25, CD69, PD-1, IFN-γ and IL-18), suggesting an antiviral activity.^976,977^ Notably, given their major locations of mucosal tissues, γδ T cells have a close interaction with microbiota, which shape γδ T cell development and function in both homeostatic and pathological conditions. The crosstalk between γδ T cells and the microbiota has been reviewed previously.^978^ Despite the innate-like signature, γδ T cells have been recently found to have memory phenotypes that they can respond rapidly with enhanced cytokine production and pathogen clearance upon the secondary infection.^979^

### γδ T cells in tumor immunity

The unique feature of γδ T cells in recognizing antigens without MHC restriction provides a promising application in cancer immunotherapy. Human γδ T cell subtypes are usually defined by δ chain, that Vδ1-3 are the most used gene segments and used for γδ T cell type classification.^980^ Vδ1 and Vδ3 T cells are less frequent γδ T cell populations and share some similarities in peripheral tissue distribution, antigen recognition and antiviral function.^981,982^ Vδ2 T cells—frequently paired between TCR Vδ2 and Vγ9 chains (Vγ9Vδ2 T cells)—constitute a predominant γδ T cell population in human peripheral blood after infection and malignancy.^983^ The phosphoantigens recognized by Vγ9Vδ2 T cells are natural products from microorganisms or generated by mammalian cells through mevalonate pathway.^981^ The aberrant mevalonate pathway in tumor cells leads to accumulation of phosphoantigens and Vγ9Vδ2 T cell activation and expansion in TME.^984^ Vγ9Vδ2 T cells recognize phosphoantigens bound by BTN3A1/BTN2A1 heterodimers.^985^ Therefore, phosphoantigen stimulation and agonism by targeting BTN3A1 have been shown to promote Vγ9Vδ2 T cell activation and anti-tumor activity.^986,987^ Non-Vγ9Vδ2 T cells, including Vδ1 and Vδ3 T cells, recognize glycolipids presented by CD1d.^988^ Besides, human γδ T cells express a range of natural killer receptors (NKRs), such as NKG2D, DNAM-1, NKp30, NKp44, and NKp46, which promote their cytotoxic effector functions upon recognition of cognate ligands on tumor cells.^982^ Moreover, γδ T cells express various TLRs and can be activated by TLR agonists to enhance cytotoxic functions.^989^

**Fig. 7:**
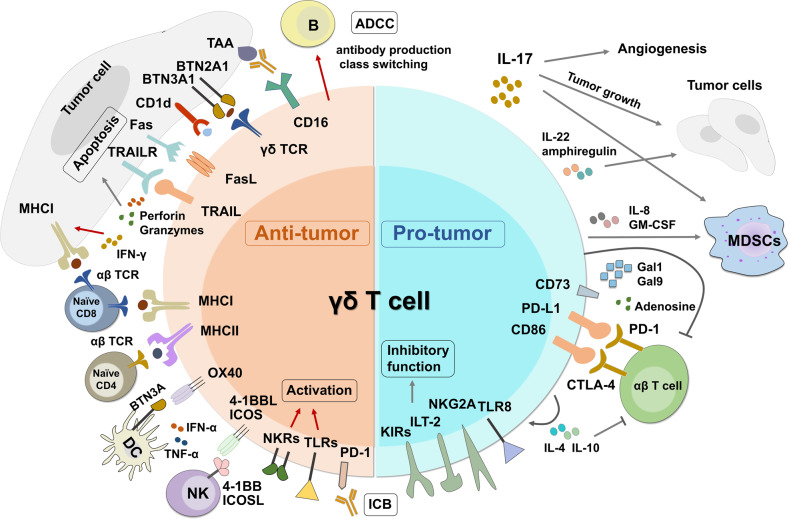
The anti- and pro-tumor immunity of γδ T cells. γδ T cells in TME play both anti- and pro-tumor activities. γδ T cells recognize phosphoantigens bound by BTN3A1/BTN2A1 heterodimers, as well as recognize glycolipids presented by CD1d. γδ T cells can directly kill tumor cells by expressing cytotoxic factors perforin and granzymes, and apoptotic receptors TRAIL and FasL. IFN-γ produced by γδ T cells enhances MHC I expression on tumor cells and their antigen presentation to CD8^+^ αβ T cells. γδ T cells are able to present antigens to CD4^+^ and CD8^+^ αβ T cells through MHC II and MHC I molecules, respectively. γδ T cells orchestrate the anti-tumor immunity through interacting and activating DCs, NK cells, and B cells. Expression of NKRs and TLRs promote γδ T cells activation and effector function. PD-1-expressing γδ T cells are the main responder to ICB in MHC I-deficient cancers. The pro-tumor activity of γδ T cells relies on both soluble factors and surface receptors by promoting tumor cell growth and angiogenesis, suppressing αβ T cell function, MDCSs induction, and inducing inhibitory functions

The function of γδ T cells in tumor immunity is versatile with both anti- and pro-tumor activities (Fig. 7). Most current evidence indicates that the presence of γδ T cells are associated with favorable outcomes in patients in CRC, breast, gastric, liver and bladder cancer, HNSCC, NSCLC and Merkel cell carcinoma.^981^ However, unfavorable prognosis of γδ T cells is also reported in CRC,^990^ gallbladder cancer,^991^ breast cancer,^992^ and acute myeloid leukemia (AML).^993^ Although different analysis techniques among studies could affect the results, at least, the γδ T types are likely associated with the prognostic prediction. Overall, IL-17^+^ γδ T cells tend to have a deleterious outcome whereas IFN-γ^+^ γδ T cells and NKR-expressing γδ T cells have improved outcomes.^991,994^ The anti-tumor activity of γδ T cells relies on multiple mechanisms^995^: (1) directly kill tumor cells by expression of perforin, granzymes and apoptotic receptors TRAIL and FasL;^996^ (2) γδ T cells upregulate CD16 (Fcγ receptor III) expression to enhance the ADCC effects of therapeutic antibodies on tumor cells;^997,998^ (3) γδ T cells have been shown to function as APCs that upon activation upregulate expression of MHC and costimulatory molecules and present antigens to CD4^+^ and CD8^+^ αβ T cells;^999–1001^ (4) γδ T cells orchestrate anti-tumor immunity through interplay with other immune cells.^1002^ IFN-γ production by γδ T cells exhibit an overall anti-tumor activity by increasing MHC I expression by tumor cells.^1003^ Vγ9Vδ2 T cells and DCs can reciprocally activate each other through both surface molecules (OX40 and BTN3A) and soluble factors (IFN-α and TNF-α).^1004,1005^ γδ T cells enhance NK cell activation and anti-tumor cytotoxicity via ICOS/ICOSL and 4-1BBL/4-1BB interaction.^1006,1007^ γδ T cells participate in humoral immunity by promoting B cell maturation, antibody production and class switching.^1008^ γδ T cells also modulate αβ T-cell activity indirectly through activating NK cells, DCs and B cells.^1002^ Intriguingly, γδ T cells are recently unveiled a critical role in mediating immune response to ICB in MHC I-deficient cancers, in which PD-1^+^ Vδ1 and Vδ3 T cells are the main contributors.^1009^

On the contrary, the pro-tumor activity of γδ T cells is largely attributed to the production of IL-17 which can promote tumor cell proliferation,^1010^ angiogenesis,^991^ accumulation of MDSCs,^990^ and create an immunosuppressive TME.^981^ In addition, pro-tumor functions of human γδ T cells may also result from expression of other mediators, such as IL-22 and amphiregulin for tumor cell growth,^1011^ PD-L1, galectins (Gal1 and 9), CD86, CD73, IL-10, and TLR8 for T cell suppression,^1012–1014^ IL-4, IL-10 and inhibitory receptors (killer Ig-like inhibitory receptors (KIRs), Ig-like transcript 2 (ILT-2), and NKG2A) for inhibitory function of Vδ T cells,^1002,1015^ and IL-8 and GM-CFS for MDSCs induction.^990^ Together, the roles of γδ T cells in the tumor milieu are complicated, and further research is required to fully elucidate the function of distinct subsets of γδ T cells to develop next-generation immunotherapies harnessing γδ T cells.

## Current immunotherapies harnessing T cell immunity

Given the central roles of T lymphocytes in health and disease, novel and effective immunotherapies harnessing the T cell immunity are under extensive development. In this section, we will briefly introduce the current immunotherapies engaging T cell function in both cancer and autoimmune disease, with an emphasis on their clinical implementation and progress.

## T cell-based cancer immunotherapy

Base on the biological roles and the modes of action, T cell-based immunotherapeutic approaches in cancer mainly include the following categories: immune checkpoint blockade (ICB) and costimulation, bispecific T cell engagers (TCEs) and adoptive cell therapy (ACT).

### ICB and costimulation

Immunomodulation of the coinhibitory and costimulatory molecules on T cells has become a powerful and effective strategy for cancer immunotherapy. Immune checkpoint molecules refer to the inhibitory receptors expressed on the immune cells and play immunosuppressive roles upon ligand interactions to maintain self-tolerance.^1016^ CTLA-4 and PD-1 are so far the most potent and successful T cell immune checkpoint molecules developed for cancer therapy in the clinic.^1017^ Since a decade ago the first U.S. Food and Drug Administration (FDA)-approved checkpoint inhibitor Ipilimumab, a monoclonal antibody (mAb) targeting CTLA-4, seven immune checkpoint inhibitors targeting PD-1/PD-L1 and another CTLA-4 mAb Tremelimumab have been consecutively approved for multiple cancer types (Table 4). Furthermore, there are nearly 6000 clinical trials assessing anti-PD-1/PD-L1 mAbs—with majority of FDA-approved ones—as monotherapy or in combination with other therapies.^1017^ Besides PD-1/PD-L1, other immune checkpoint pathways have been developed in the clinic for cancer therapy, including but not limited to Lag-3, TIGIT, Tim-3, CD96, BTLA, VISTA and B7H3.^1018,1019^ Among them, the anti-Lag-3 mAb (Telatlimab) has been approved firstly by FDA for metastatic melanoma in combination with anti-PD-1 mAb.^1020,1021^ Moreover, the advanced candidates in phase III clinical trials are mAbs targeting Lag-3, TIGIT and Tim-3 (Table 5). In contrast to inhibitory checkpoints, costimulatory molecules provide critical signals for effective T cell responses and function, making them promising therapeutic targets.^1022^ Thus, mAbs targeting costimulatory receptors, such as GITR, 4-1BB, ICOS, CD27, CD28, and OX40, are also under evaluation in clinical trials.^1023^ However, agonist antibodies have not exhibited much clinical benefits.^1024^ So far, most of the programs targeting costimulatory pathways are in early clinical phases except for one ICOS-stimulatory mAb Feladilimab entering phase III trial (Table 5).

**Table 4:** T cell-based therapies approved in the market

Therapy type	Modality	Product name	Brand	Target	Indications	Company	Approval date
ICB	mAbs	Ipilimumab	Yervoy	CTLA-4	Multiple cancer types	BMS	FDA 2010
		Tremelimumab	Imjudo	CTLA-4	Hepatocellular carcinoma	AstraZeneca	FDA 2022
		Pembrolizumab	Keytruda	PD-1	Multiple cancer types	Merck	FDA 2014
		Nivolumab	Opdivo	PD-1	Multiple cancer types	BMS	FDA 2014
		Cemiplimab	Libtayo	PD-1	Multiple cancer types	Sanofi	FDA 2018
		Dostarlimab	Jemperli	PD-1	Multiple cancer types	GlaxoSmithKline	FDA 2021
		Atezolizumab	Tecentriq	PD-L1	Multiple cancer types	Genentech/Roche	FDA 2016
		Avelumab	Bavencio	PD-L1	Multiple cancer types	EMD	FDA 2016
		Durvalumab	Imfinzi	PD-L1	Multiple cancer types	AstraZeneca	FDA 2016
	Combination	Relatlimab+Nivolumab	Opdualag+Opdivo	Lag-3+PD-1	Metastatic melanoma	BMS	FDA 2022
	bsAbs	Cadonilimab	-	PD-1xCTLA-4	Metastatic cervical cancer	Akeso Biopharma	NMPA 2022
TCE	bsAbs	Blinatumomab	Blincyto	CD19xCD3	r/r ALL	Amgen	FDA 2014
		Mosunetuzumab-axgb	Lunsumio	CD20xCD3	Follicular lymphoma	Genentech/Roche	FDA 2022
		Teclistamab-cqyv	Tecvayli	BCMAxCD3	r/r MM	Janssen Biotech	FDA 2022
		Elranatamab	-	BCMAxCD3	r/r MM	Pfizer	FDA Filing Acceptance 2023
	TCR-like antibody	Tebentafusp-tebn	Kimmtrak	HLA-A*02:01/gp100 complex	Uveal melanoma	Immunocore	FDA 2022
ACT	CAR-T	Tisagenlecleucel	Kymriah	CD19	ALL, DLBCL	Novartis	FDA 2017
		Axicabtagene ciloleucel	Yescarta	CD19	NHL, DLBCL	Kite/Gilead	FDA 2017
					DLBCL		NMPA 2021
		Brexucabtagene autoleucel	Tecartus	CD19	MCL, ALL	Kite/Gilead	FDA 2020
		Lisocabtagene maraleucel	Breyanzi	CD19	DLBCL	Juno Therapeutics/BMS	FDA 2021
		Idecabtagene vicleucel	Abecma	BCMA	MM	Bluebird Bio/BMS	FDA 2021
		Ciltacabtagene autoleucel	Carvykti	BCMA	MM	Legend/Janssen Biotech	FDA 2022
		Relmacabtagene autoleucel	Carteyva	CD19	DLBCL	JW Therapeutics	NMPA 2021

*FDA* Food and Drug Administration, *NMPA* National Medical Products Administration of China, *r/r ALL* relapsed/refractory B cell precursor acute lymphoblastic leukemia, *r/r MM* relapsed/refractory multiple myeloma, *DLBCL* diffuse large B-cell lymphoma, *NHL* non-Hodgkin’s lymphoma, *MCL* mantle cell lymphoma

**Table 5:** Selected clinical-stage T cell-based immunotherapies

Therapy type	Modality	Product name	Target	Disease	Clinical trial identifier	Sponsor	Phase
ICB	mAb	Tiragolumab	TIGIT	NSCLC	NCT04294810	Roche	III
		Ociperlimab	TIGIT	NSCLC	NCT04746924	BeiGene	III
		MBG453	Tim-3	Myelodysplastic Syndromes	NCT04266301	Novartis Pharmaceuticals	III
		Fianlimab	Lag-3	Melanoma	NCT05608291	Regeneron Pharmaceuticals	III
		Feladilimab	ICOS	Neoplasms, Head and Neck	NCT04428333	GlaxoSmithKline/Merck	II/III
	bsAb	KN046	PD-L1xCTLA-4	NSCLC	NCT04474119	Jiangsu Alphamab Biopharmaceuticals	III
		Tebotelimab	PD-1×Lag-3	Gastric Cancer	NCT04082364	MacroGenics	II/III
		Lomvastomig	PD-1xTim-3	Advanced or Metastatic ESCC	NCT04785820	Roche	II
		FS118	PD-L1×Lag-3	Advanced Cancer	NCT03440437	F-star Therapeutics	I/II
		XmAb22841	CTLA-4xLag-3	Metastatic Melanoma	NCT05695898	Xencor	I/II
		HLX301	TIGITxPD-L1	Advanced Tumors	NCT05390528	Shanghai Henlius Biotech	I/II
		AZD2936	TIGITxPD-1	NSCLC	NCT04995523	AstraZeneca	I/II
		GEN1046	PD-L1x4-1BB	Solid Tumors	NCT03917381	Genmab	I/II
		PRS-344/S095012	PD-L1x4-1BB	Solid Tumor	NCT05159388	Pieris Pharmaceuticals	I/II
		XmAb23104	PD-1xICOS	Metastatic Melanoma	NCT05695898	Xencor	I/II
		Ivonescimab (AK112)	PD-1xVEGF	Advanced NSCLC	NCT05499390	Akeso	III
		PM8002	PD-1xVEGF	NSCLC	NCT05756972	Biotheus	II/III
		Bintrafusp alfa (M7824)	PD-L1xTGFβRII	NSCLC	NCT03631706	Merck KGaA	III
		SHR-1701	PD-L1xTGFβRII	Advanced or Metastatic NSCLC	NCT05132413	Jiangsu Hengrui Medicine/Suzhou Suncadia Biopharmaceuticals	III
TCE	TAAxCD3	Epcoritamab	CD20xCD3	DLBCL	NCT04628494	Genmab/AbbVie	III
		Elranatamab	BCMAxCD3	MM	NCT05317416	Pfizer	III
		Glofitamab	CD20xCD3	DLBCL	NCT04408638	Roche	III
		Teclistamab	BCMAxCD3	MM	NCT05083169	Janssen Research	III
		Linvoseltamab	BCMAxCD3	MM	NCT05730036	Regeneron Pharmaceuticals	III
		Talquetamab	GPRC5DxCD3	MM	NCT05455320	Janssen Research	III
		Catumaxomab	EpCAM x CD3	Stomach Neoplasms	NCT04222114	LintonPharm	III
		Tarlatamab	DLL3xCD3	SCLC	NCT05740566	Amgen	III
		CC-1	PMSAxCD3	Lung Cancer Squamous Cell	NCT04496674	German Cancer Research Center	I/II
		REGN4336	PSMAXCD3	Prostate Cancer	NCT05125016	Regeneron Pharmaceuticals	I/II
		REGN4018	MUC16xCD3	Ovarian Cancer	NCT03564340	Regeneron Pharmaceuticals	I/II
		EGFR BATs	EGFRxCD3	Pancreatic Adenocarcinoma	NCT03269526	University of Virginia	I/II
		Cibisatamab	CEAxCD3	Colorectal Cancer	NCT03866239	Roche	I/II
		Runimotamab	HER2xCD3	HER2-expressing Solid Tumors	NCT03448042	Genentech	I
		AMG 596	EGFRvIII and CD3	Glioblastoma or Malignant Glioma	NCT03296696	Amgen	I
		GEM3PSCA	PSCA and CD3	PSCA-positive solid cancers	NCT03927573	AvenCell Europe GmbH	I
		ERY974	GPC3xCD3	HCC	NCT05022927	Chugai Pharmaceutical	I
	TAAx Costimulation	REGN5668	MUC16xCD28	Ovarian Cancer	NCT04590326	Regeneron Pharmaceuticals	I/II
		REGN5678	PSMAxCD28	Metastatic Castration-resistant Prostate Cancer	NCT03972657	Regeneron Pharmaceuticals	I/II
		REGN7075	EGFRxCD28	Advanced Solid Tumors	NCT04626635	Regeneron Pharmaceuticals	I/II
		GEN1046	PD-L1/4-1BB	NSCLC	NCT05117242	Genmab/BioNTech SE	II
		PRS-343	HER2/4-1BB	HER2-positive Gastric Cancer	NCT05190445	Pieris Pharmaceuticals	II
		HLX35	EGFR×4-1BB	Advanced or Metastatic Solid Tumors	NCT05360381	Shanghai Henlius Biotech	I
		CB307	PSMA×4-1BB	Advanced and/or Metastatic Solid Tumors	NCT04839991	Crescendo Biologics	I
		RO7122290	FAPx4-1BB	Metastatic Colorectal Cancer	NCT04826003	Roche	I/II
		BT7480	Nectin-4×4-1BB	Advanced Solid Tumor	NCT05163041	BicycleTx Limited	I/II
ACT (Cancer)	CAR-T	CAR-T CD19	CD19	Acute Myeloid Leukemia	NCT04257175	Sheba Medical Center	III
		CAR-T-CD19 Cells	CD19	DLBCL	NCT05020392	Wuhan Union Hospital, China	III
		CD19 CAR-T CELLS	CD19	Relapsed ALL	NCT03937544	National University of Malaysia/Gaia Science	III
		BCMA CAR-T-cells	BCMA	MM	NCT04287660	The First Affiliated Hospital of Soochow University (and 13 more)	III
		JNJ-68284528	BCMA	MM	NCT04181827	Janssen Research & Development	III
		bb2121	BCMA	MM	NCT03651128	Celgene	III
		fhB7H3.CAR-Ts	B7H3 (CD276)	Ovarian Cancer	NCT05211557	The Affiliated Hospital of Xuzhou Medical University	I/II
		CD276 CAR-T cells	B7H3 (CD276)	Advanced Pancreatic Carcinoma	NCT05143151	Shenzhen University General Hospital	I/II
		anti-MESO CAR-T cells	Mesothelin	Ovarian Cancer	NCT03916679	Second Affiliated Hospital, Zhejiang University	I/II
		ALPP CAR-T	alkaline phosphatase	Endometrial Cancer	NCT04627740	Xinqiao Hospital of Chongqing/TCRCure Biopharma	I/II
		CNA3103	LGR5	Colorectal Cancer Metastatic	NCT05759728	Carina Biotech Pty	I/II
		CT041	Claudin18.2	Gastric Cancer/Pancreatic Cancer	NCT04404595	CARsgen Therapeutics	I/II
		RD14-01	ROR1	Solid Tumor	NCT05748938	920th Hospital of Joint Logistics Support Force of People’s Liberation Army of China	I/II
		CEA CAR-T	CEA	Solid Tumor	NCT04348643	Chongqing Precision Biotech	I/II
		IVS-3001-Anti-HLA-G CAR-T	HLA-G	Solid Tumor	NCT05672459	M.D. Anderson Cancer Center	I/II
		BPX-601	PSCA	Prostate Cancer	NCT02744287	Bellicum Pharmaceuticals	I/II
		HypoSti.CAR-HER2 T cells	HER2	HER2-Positive Solid Tumors	NCT05681650	Chinese PLA General Hospital	I/II
		CLDN6 CAR-T	Claudin6	Solid Tumor	NCT04503278	BioNTech Cell & Gene Therapies GmbH	I/II
		GD2-CART01	GD2	Neuroblastoma	NCT03373097	Bambino Gesù Hospital and Research Institute	I/II
		MUC1 CAR-T	MUC1	Intrahepatic Cholangiocarcinoma	NCT03633773	Second Affiliated Hospital	I/II
		BOXR1030	Glypican 3	GPC3-Positive Solid Tumors	NCT05120271	SOTIO Biotech	I/II
	Bispecific CAR-T	bi-4SCAR CD19/22 T cells	CD19/CD22	B Cell Malignancies	NCT05432882	Shenzhen Geno-Immune Medical Institute	I/II
		bi-4SCAR CD19/70 T cells	CD19/CD70	B Cell Malignancies	NCT05436496	Shenzhen Geno-Immune Medical Institute	I/II
		bi-4SCAR CD19/79b T cells	CD19/CD79b	B Cell Malignancies	NCT05436509	Shenzhen Geno-Immune Medical Institute	I/II
		CAR-20/19-T Cells	CD19/CD20	B Cell Malignancies	NCT04186520	Medical College of Wisconsin	I/II
		bi-4SCAR GD2/CD70 T cells	GD2/CD70	Cancer Disease	NCT05438368	Shenzhen Geno-Immune Medical Institute	I/II
		bi-4SCAR GD2/PSMA T cells	GD2/PSMA	Solid Tumor	NCT05437315	Shenzhen Geno-Immune Medical Institute	I/II
		bi-4SCAR PSMA/CD70 T cells	PSMA/CD70	Cancer Disease	NCT05437341	Shenzhen Geno-Immune Medical Institute	I/II
		Dual-targeting VEGFR1 and PD-L1 CAR-T cells	VEGFR1/PD-L1	Malignant Peritoneal Effusion	NCT05477927	Sichuan University	I
		EGFR/B7H3 CAR-T	EGFR/B7H3	Advanced Lung Cancer/TNBC	NCT05341492	Second Affiliated Hospital of Guangzhou Medical University	Early I
		Dual-targeting HER2 and PD-L1 CAR-T cells	HER2/PD-L1	Peritoneal Carcinoma Metastatic	NCT04684459	Sichuan University	Early I
	TCR-T	anti-MART-1 F5 T-cell receptor	MART-1	Melanoma	NCT00509288	National Cancer Institute (NCI)	II
		Anti-gp100:154-162 TCR	gp100	Melanoma	NCT00923195	National Cancer Institute (NCI)	II
		PG13-CEA_TCR	CEA	Metastatic Cancer	NCT00923806	National Cancer Institute (NCI)	I
		WT1 TCR transduced T cells	WT1	MDS/AML	NCT02550535	Cell Medica	I/II
		afamitresgene autoleucel	MAGE-A4	Synovial Sarcoma	NCT04044768	Adaptimmune Therapeutics	II
		Anti-MAGE-A3-DP4 TCR PBL	MAGE-A3	Cervical Cancer	NCT02111850	National Cancer Institute (NCI)	I/II
		autologous MC2 TCR-T cells	MAGE-C2	Melanoma and Head and Neck Cancer	NCT04729543	Erasmus Medical Center (and 4 more)	I/II
		CD8 + T-cells, transduced with MAGE-A1 directed TCR	MAGE-A1	Advanced Solid Tumors	NCT05430555	knife GmbH	I/II
		letetresgene autoleucel	NY-ESO-1	Neoplasms	NCT02992743	GlaxoSmithKline	II
		NY-ESO-1c259 T cells	NY-ESO-1	Ovarian Cancer	NCT01567891	Adaptimmune	I/II
		NY-ESO-1(TCR Affinity Enhancing Specific T cell Therapy)	NY-ESO-1	Soft Tissue Sarcoma	NCT05549921	Sun Yat-sen University	II
		E7 TCR-T cells	HPV E7	HPV Associated Cancers	NCT05686226	The State University of New Jersey	II
		TC-E202 cells	HPV-16 E6	Cervical Carcinoma	NCT05357027	TCRCure Biopharma/Fudan University	I/II
		E6 TCR	HPV-16 E6	HPV Associated Cancers	NCT02280811	National Cancer Institute (NCI)/Kite Pharma	I/II
		TCR redirected T cells	HBV	Hepatocellular Carcinoma	NCT03899415	Beijing 302 Hospital/Lion TCR Pte	I
		MCPyV-specific HLA-A02-restricted TCR T	MCPyV	Metastatic or Unresectable MCC	NCT03747484	Fred Hutchinson Cancer Center/National Cancer Institute (NCI)	I/II
		EBV-specific TCR-T	EBV	HNSCC	NCT04139057	Xinqiao Hospital of Chongqing/TCRCure Biopharma	I/II
		anti-p53 T-cell receptor transduced peripheral blood lymphocytes	Tumor protein 53 (p53)	Metastatic Cancer	NCT00393029	National Cancer Institute (NCI)	II
		Mutant KRAS G12V-specific TCR transduced autologous T cells	Mutant KRAS G12V	Pancreatic Cancer	NCT04146298	Changhai Hospital	I/II
		anti-KRAS G12D mTCR PBL	Mutant KRAS G12D	Gastrointestinal Cancer/Pancreatic Cancer	NCT03745326	National Cancer Institute (NCI)	I/II
		TC-510 T Cells	Mesothelin	Mesothelioma	NCT05451849	TCR2 Therapeutics	I/II
		Gavo-cel (TC-210) T Cells	Mesothelin	Mesothelioma	NCT03907852	TCR2 Therapeutics	I/II
		TC-110 T Cells	CD19	NHL	NCT04323657	TCR2 Therapeutics	I/II
	TILs	Tumor Infiltrating Lymphocytes (TIL)	-	Metastatic Melanoma	NCT02278887	The Netherlands Cancer Institute	III
		Lifileucel (LN-144)	-	Metastatic Melanoma	NCT02360579	Iovance Biotherapeutics	II
		LN-145	-	Metastatic TNBC	NCT04111510	Iovance Biotherapeutics	II
		Tumor Infiltrating Lymphocytes (TIL)	-	BTC	NCT03801083	Udai Kammula	II
		Young TIL	-	Metastatic Colorectal/Pancreatic/Ovarian Cancer	NCT01174121	National Cancer Institute (NCI)	II
		Tumor Infiltrating Lymphocytes (TIL)	-	Uveal Melanoma	NCT03467516	Udai Kammula	II
		Young TIL	-	Advanced NSCLC	NCT02133196	National Cancer Institute (NCI)	II
		Tumor Infiltrating Lymphocytes (TIL)	-	Multiple advanced Solid Cancers	NCT03935893	Udai Kammula	II
		Super circulating TIL (ScTIL)	-	Gynecological Malignancies	NCT05342506	Peking Union Medical College Hospital	II
		Tumor Infiltrating Lymphocytes (TIL)	-	Metastatic Urothelial Carcinoma	NCT04383067	Sheba Medical Center	II
		Tumor Infiltrating Lymphocytes (TIL)	-	Gastrointestinal Cancer	NCT04426669	Intima Bioscience, Inc.	I/II
		Autologous tumor infiltrating lymphocytes MDA-TIL	-	Multiple advanced Solid Cancers	NCT03610490	M.D. Anderson Cancer Center	II
ACT (Autoimmunity)	CAR-T	YTB323	CD19	SLE/Lupus Nephritis	NCT05798117	Novartis Pharmaceuticals	I/II
		CT103A cells	BCMA	Autoimmune Diseases	NCT04561557	Tongji Hospital/Nanjing IASO Biotherapeutics	Early I
		Descartes-08	BCMA	MG	NCT04146051	Cartesian Therapeutics	II
		CD19/BCMA CAR-T-cells	CD19/BCMA	POEMS Syndrome/Amyloidosis/Autoimmune Hemolytic Anemia/Vasculitis	NCT05263817	Zhejiang University/Yake Biotechnology	Early I
				SLE	NCT05030779		Early I
				Sjogren’s Syndrome	NCT05085431		Early I
				Immune Nephritis	NCT05085418		Early I
		BCMA-CD19 cCAR T cells	CD19/BCMA	Relapsed/Refractory, SLE	NCT05474885	iCell Gene Therapeutics	I
		DSG3-CAAR-T	DSG3	Mucosal-Dominant PV	NCT04422912	Cabaletta Bio	I
		MuSK-CAAR-T	Musk	MuSK-MG	NCT05451212	Cabaletta Bio	I
	CAR-Treg	TX200-TR101	HLA-A*02	Kidney Transplant Rejection	NCT04817774	Sangamo Therapeutics	I/II
		QEL-001	HLA-A*02	Rejection; Transplant, Liver	NCT05234190	Quell Therapeutics	I/II

*NSCLC* non-small cell lung cancer, *ESCC* esophageal squamous cell carcinoma, *DLBCL* diffuse large B-cell lymphoma, *MM* multiple myeloma, *SCLC* small cell lung cancer, *HCC* hepatocellular carcinoma, *ALL* B acute lymphoblastic leukemia, *TNBC* triple-negative breast cancer, *MDS* myelodysplastic syndromes, *AML* acute myeloid leukemia, *MCC* Merkel cell cancer, *HNSCC* head and neck squamous cell carcinoma, *NHL* non-Hodgkin’s lymphoma, *BTC* biliary tract cancer, *SLE* systemic lupus erythematosus, *MG* myasthenia gravis, *PV* pemphigus vulgaris

(Source: clinicaltrials.gov)

### Bispecific T cell engagers (TCEs)

Emerging evidence has demonstrated that simultaneously targeting two or multiple immunomodulatory molecules display potent anti-tumor activity while reduce toxicity, leading to the revolutionary development of bispecific antibodies (bsAbs) or even trispecific antibodies (TsAbs).^1025,1026^ With the advances in antibody engineering, numerous formats have been exploited for bsAb design (reviewed in ref. 1026). Different from a combination of two mAbs, bsAbs can either bind to two molecules expressed on one cell (in-cis binding) or bridge two distinct cells (in-trans binding) to further enhance the therapeutic efficacy.^1026^ The mechanisms of action of bsAbs engaging T cells mainly include four types: (1) dual-targeting inhibitory checkpoint molecules; (2) targeting both costimulatory and inhibitory checkpoints; (3) targeting checkpoints with non-checkpoint molecules; (4) directly targeting T cells by TCE. Dual-targeting inhibitory checkpoints usually occurs between PD-1/PD-L1 and other checkpoint molecules under clinical assessment, such as CTLA-4, Lag-3, Tim-3, and TIGIT.^1026,1027^ Notably, Cadonilimab, a bsAb targeting PD-1×CTLA-4, is the first bsAb approved by Chinese National Medical Products Administration (NMPA) last year for treating relapsed or metastatic cervical cancer (r/mCC)^1028^ (Table 4). Besides, KN046 and Tebotelimab, targeting PD-L1×CTLA-4 and PD-1×LAG-3 respectively, are the most advanced bsAb candidates in late-phase clinical trials (NCT04474119 and NCT04082364) (Table 5). Other bsAbs, such as PD-1xTim-3, PD-L1×Lag-3, PD-(L)1xTIGIT, and CTLA-4xLag-3, are under evaluation in phase I/II studies (Table 5). Co-targeting checkpoint inhibitors and costimulatory molecules has a synergistic effect on enhancing T cell function and therapeutic efficacy. BsAbs in this category, including GITR×CTLA-4, 4-1BB×PD-L1,^1029^ OX40×PD-L1,^1030^ OX40×CTLA-4,^1031^ ICOS×PD-L1, and CD27×PD-L1,^1032^ are mainly under early clinical assessment. The non-checkpoint targets involved in bsAbs are mostly tumor-associated antigens (TAAs) and pro-tumor growth factors/cytokines.^1027^ Targeting TAAs can increase the tumor selectivity of immunomodulatory molecules and alleviate systemic toxicity, whereas inhibiting growth factors/cytokines further enhances the efficacy of tumor eradication. TAAs used for immune checkpoint targeting include EpCAM (CD40×EpCAM), EGFR (PD1×EGFR) and HER2 (PD1×HER2).^1033,1034^ The widely used growth factors/cytokines are pro-angiogenic VEGF and immunosuppressive TGF-β. BsAbs under late-phase clinical development are PD-1xVEGF (AK112 and PM8002) and PD-L1xTGFβRII (M7824 and SHR-1701) (Table 5). Of note, despite the rationale behind ‘trapping’ TGF-β for cancer therapy,^1035^ the unsatisfied clinical results of M7824 (also known as Bintrafusp alfa) in NSCLC and biliary tract cancers (BTCs)^1036^ raise the concern of TGF-β-targeting strategy, and further research is required to fully understand the biology of TGF-β in TME.

TCEs, also referred to bispecific T cell engagers (BiTEs), are designed bsAbs co-targeting CD3ε and specific tumor antigens to redirect cytotoxic T cells against tumor cells. Various TCE formats and platforms have been developed and reviewed elsewhere.^1037,1038^ TCEs activate T cells independent on MHC restriction and TCR epitope specificity and have been developed rapidly and extensively over the years, becoming a promising immunotherapy. To date, three BiTEs have been approved by FDA in the market: Blinatumomab (Blincyto; CD19×CD3; Amgen) in 2014 for patients with relapsed/refractory (r/r) B cell precursor acute lymphoblastic leukemia (ALL), Mosunetuzumab-axgb (Lunsumio; CD20xCD3; Roche) for follicular lymphoma, and Teclistamab-cqyv (Tecvayli; BCMAxCD3; Janssen Biotech) for r/r multiple myeloma (MM) in 2022. In addition, Elranatamab (BCMAxCD3; Pfizer) for r/r MM has received FDA and European Medicines Agency (EMA) filing acceptance which is expected to be approved in 2023 (Table 4). Apparently, FDA-approved TCEs and majority of the late-phase TCEs target antigens in hematological malignancies^1039^ (Table 5). Other hematological tumor targets in early-phase studies include CD38, CD123, CD30, CD33, FcRH5, FLT3, and CLEC12A.^1026^ However, compare to liquid tumors, development of TCEs against solid tumors are much challenging. Two bsAbs Catumaxomab (EpCAM×CD3) and Tarlatamab (DLL3×CD3) are so far in phase III studies, while other TCEs targeting PSMA, MUC16, EGFR, CEA, HER2, EGFRvIII, PSCA, and GPC3 are mostly in early-phase trails (Table 5). The immunological mechanisms underlying T cell response or non-response to TCEs are not fully understood. A recent clinical study in MM patients using BCMAxCD3 TCE has revealed that the pre-existing T cell landscape determines the response to TCE. Moreover, effector and naïve CD8^+^ T cells drive the immunological response to TCE while the exhausted CD8^+^ T cells are highly associated with the response failure.^1040^ One key challenge of CD3-TCEs in treating solid tumor is the treatment-mediated toxicity, including both cytokine release syndrome (CRS) and on-target/off-tumor toxicity.^1037,1041,1042^ Several strategies to overcome the adverse events of TCEs in solid tumors are under both clinical and preclinical investigations. One important approach is targeting peptide/MHC (pMHC) complexes, known as TCR mimetic antibodies. Indeed, Tebentafusp (Kimmtrak; Immunocore), a CD3 BiTE with TCR arm recognizing glycoprotein 100 (gp100) peptide presented by HLA-A*02:01, gained FDA approval in 2022 for the treatment of HLA-A*02:01-positive patients with unresectable or metastatic uveal melanoma.^1043^ The success of Tebentafusp has also become a major milestone for TCR-based immunotherapies. Another approach is developing conditional TCEs which are inactive prodrugs upon administration and gain activation in a tempo-spatial controlled manner within TME, such as TCEs with a masking on the binding domain.^1038^

In addition to CD3, alternative approaches targeting costimulatory molecules on T cells, such as CD28 and 4-1BB, have also implemented for TCE development. Engagement of costimulatory receptors mimics signal 2 for T cell activation. Costimulatory BiTEs targeting a variety of solid tumors are currently evaluated in phase I/II trials: MUC16, PSMA, EGFR, PD-L1, HER2, Nectin-4, and FAP (targeting tumor-associated fibroblasts) (Table 5). 4-1BB costimulation has been demonstrated to remarkedly improve T cell survival, activation and effector function, which occurs preferentially in CD8^+^ T cells.^1044^ TAAxCD28 BiTEs, when combined with TAAxCD3 BiTEs, could significantly enhance T cell activation and the anti-tumor activity of the CD3 BiTEs.^1045^ The intracellular domains of CD28 and 4-1BB are widely implemented in the CAR-T cell generation; CD28 and 4-1BB differ in both expression pattern on T cells as well as the intracellular signal cascade.^1046^ Further research especially results from clinical studies will help us to better understand the underlying mechanism of these costimulatory signals in cancer immunotherapy.

### Adoptive cell therapy (ACT)

In addition to drugs that modulate T cell function, direct T cell adoptive transfer of autologous or allogenic T cells into patients has shown substantial promise in cancer immunotherapies. According to different T cell source and ways of antigen recognition, ACT mainly divide into three types: chimeric antigen receptor (CAR)-T cells, TCR-T cells, and tumor infiltrating lymphocyte (TIL) therapy. Generally, TIL therapy is adoptively transferring tumor-specific TILs that are isolated from tumor tissues and amplified ex vivo, whereas CAR-T cell and TCR-T cell therapies are based on T cells that are genetically engineered to express receptors recognizing antigens.

CAR-T cell therapy is one of the most prevalent and advanced types of ACT. CARs are normally engineered proteins targeting tumor antigens to enhance the tumor-killing specificity and efficacy of immune cells, such as T cells, NK cell and macrophages. A classic CAR is composed of an extracellular antigen-binding domain, a hinge, a transmembrane region, one or more costimulatory domains, and an activation domain. The antigen-binding domain consists of a single-chain variable fragment (scFv) recognizing antigens. The costimulatory domains—CD28 and/or 4-1BB—are designed to augment T cell activation, proliferation and effector function. The activation domain is usually the CD3ζ domain which transduces activation signaling for T cells.^1047^ The structural engineering of CAR-T cells has been gone through five generations with distinct intracellular functional domains. In addition to the basic CAR components mentioned above, the fourth and fifth generation of CAR-T cells contain cytokines or intracellular domains of cytokine receptors, which can further enhance the effector function of T cell or adaption to the immunosuppressive TME.^1048^

In the past two decades, CAR-T cell therapy has obtained tremendous clinical success in treating cancers particularly in patients with hematological tumors. To date, seven CAR-T products with five targeting CD19 and two for BCMA have been approved in the market (Table 4). Candidates in clinical phase III pipeline are also targeting CD19 or BCMA (Table 5). CAR-T therapies targeting antigens in solid tumors are then assessed in early-phase clinical studies, such as B7H3 (CD276), mesothelin, alkaline phosphatase, LGR5, Claudin18.2, ROR1, CEA, HLA-G, PSCA, HER2, Claudin6, GD2, MUC1, and Glypican 3^1049^ (Table 5). Like TCEs, CAR-T therapy faces challenges in solid tumors due to multiple reasons: tumor antigen heterogeneity and escape, toxicity, inefficient tumor infiltration, poor persistency, and immunosuppressive TME.^1048^ Next-generation CAR-T cells for overcoming those challenges are under extensive investigations.^1049,1050^ For instance, to avoid tumor-antigen escape as well as off-target toxicity, dual CARs are designed to co-targeting two different tumor antigens, such as CD19/CD22, CD19/CD22, GD2/CD70, GD2/PSMA, EGFR/B7H3, etc. (Table 5). Another creative approach is applying Boolean logic to CAR-T cells, which can conditionally control T cell activity to increase T cell specificity and limit off-target toxicity.^1051,1052^ The logic-gates consist of OR-gate, AND-gate, NOT-gate, IF-THEN-gate and IF-BETTER-gate, and can be engineered to have constitutive expression or inducible expression.^1053–1055^ Most of the logic-gate CAR-T constructs have not yet been tested in the clinic except for IMPT-314, a CD19/CD20-targeted bispecific “OR-Gate” CAR-T therapy which has just gained FDA approval this year in patients with aggressive B-cell lymphoma. Some future directions for advancing CAR-T therapies include but not limit to improving CAR-T cell persistency, function and tumor infiltration, combination with other therapies, and development of allogeneic/universal CAR-T cells.^1048–1050^

Despite the potency, CAR-T cells target only surface antigens. In contrast, TCR-T cells can recognize intracellular antigens, which greatly increases the tumor target repertoire. TCR-T cells are much more (at least 100-fold) sensitive to antigens that a low antigen density is sufficient to activate TCR-T cells.^1056,1057^ In addition, TCR-T cells adopt a near-to-physiological signaling pathway compared to CAR-T cells.^1056^ Such enhanced sensitivity and avidity of TCR-T cells markedly improve their tumor cell recognition and killing efficacy. However, TCR-T cells recognize peptide/HLA complexes with HLA restriction, which limits their application in certain patient populations. Currently, TCR-T cell therapies have not yet been approved in the market but are assessed in early-phase clinical trials (Table 5). Given the high sensitivity of antigen detection, antigen selection is crucial for developing safe TCR-T therapies. According to the biological function, tumor antigens developed and evaluated for TCR-T therapy in the clinical trials are tissue differentiation antigens (MART-1, gp100, CEA and WT1), cancer germline antigens (MAGE-A and NY-ESO-1), viral antigens (HPV, HBV, Merkel cell polyomavirus (MCPyV), and EBV), mutation-associated neoantigens (p53, KRAS^G12V^, and KRAS^G12D^) as well as TAAs (mesothelin and CD19) (Table 5). TCR-T cell therapy also faces challenges such as treatment-associated toxicity, tumor antigen escape, low tumor infiltration and suppressive tumor milieu.^1058^ Besides, identification of tumor epitope-specific TCRs is complex. The advances of high-throughput screening using peptide libraries and barcoded tetramers and scTCR-seq facilitate the identification of antigen-specific TCRs.^1059–1061^

TILs, compared to non-TILs, display mostly effector memory T cell phenotype, can be activated and expanded ex vivo, and possess chemokine receptors for migration toward TME, thus severing great immunological reactivity against tumor cells.^1062,1063^ Although TILs can be separated from resected solid tumor tissues, the cell number is inadequate for cancer immunotherapy. High dose IL-2 exposure and nonmyeloablative lymphodepletion are key procedures to provide enough TILs for infusion and enhance the therapeutic effectiveness.^1064,1065^ Currently, TIL therapy has been evaluated in the clinical studies in multiple solid tumor types, such as melanoma, breast cancer, biliary tract cancer, CRC, NSCLC, gastrointestinal, and gynecological cancers (Table 5). Though no TIL therapy has been approved yet, the most advanced TIL product is lifileucel (LN-144), developed by Iovance Biotherapeutics, and has just completed its Biologics License Application (BLA) submission for unresectable or metastatic melanoma. Notably, the BLA application for lifileucel is supported by positive clinical data of a phase II study (C-144-01).^1066^ Besides the common challenges for T cell therapies, TIL therapy faces a key obstacle of TIL preparation. TIL therapy is the most personalized treatment; therefore, the specific TILs product must be prepared for each patient.^1067^ Several strategies have been developed to overcome this issue, such as CD8^+^ enriched young TILs,^1068^ rapid expansion by anti-CD3 antibody, IL-2 and feeder cells,^1069^ generating artificial APCs for TIL expansion,^1070^ and incorporation of costimulatory signals.^1071^ Additionally, combination of TILs with other anti-tumor therapies are also developed and tested in clinical and preclinical studies.^1072^

## T cell-based immunotherapies in autoimmune diseases

For autoimmune diseases, traditional therapeutic drugs mainly include three classes: nonsteroid anti-inflammatory drugs (NSAIDs), steroid anti-inflammatory drugs (SAIDs), and disease-modifying antirheumatic drugs (DMARDs). While NSAIDs and SAIDs are effective for pain relief and inflammation inhibition, DMARDs are mainly reducing the tissue damages caused by severe inflammation.^1073^ In recent decades, biological drugs targeting inflammatory cytokines, receptors and signaling molecules have been developed and displayed great effectiveness.^652,1074^ Among all, Th1- and Th17-associated cytokines, such as TNF-α, IL-12, IL-6, IL-23, and IL-17, are critical for the development and pathogenesis of autoimmune diseases, thus, have been extensively studied and developed for treating multiple autoimmune diseases. A number of neutralizing antibodies or fusion proteins targeting inflammatory signaling pathways have been approved in the market: TNF-α (Infliximab, Etanercept, Adalimumab, Certolizumab, and Golimumab), IL-12/IL-23 (Ustekinumab), IL-6 (Siltuximab), IL-6R (Tocilizumab, Sarilumab, and Satralizumab), IL-23 (Guselkumab, Tildrakizumab, and Risankizumab), IL-17 (Secukinumab and Ixekizumab), and IL-17RA (Brodalumab).^1075,1076^ The JAK-STAT pathways, mediating the intracellular signal transduction downstream of cytokine receptors, have also been targeting by small molecule inhibitors for autoimmune diseases.^1077,1078^ In addition, B cell depletion by mAbs targeting various B cell types, such as anti-CD19, anti-CD20 and anti-CD22, have shown beneficial effects in autoimmune disorders.^1079^

### CAR-T and CAAR-T cell therapy

Intriguingly, CAR-T cell-based immunotherapies have emerged increasing interest in autoimmune diseases and demonstrated promising clinical efficacy.^1080,1081^ Based on the recognition specificity of CARs, four strategies have been developed for CAR-T therapies in autoimmune manifestation: (1) CAR-T cells targeting autoreactive B cells; (2) Chimeric autoantibody receptor T cells (CAAR-T cells) expressing autoantigens that interact with autoantibodies on B cells; (3) CAR-T cells expressing pathogenic pMHC complexes recognized by autoreactive T cells; (4) CAR-T_reg_ cells recognizing autoantigens and exerting immunosuppressive activity.^1082,1083^ B cell depletion has become an important therapeutic strategy in autoimmune diseases.^1084^ CAR-T cells targeting pan-B cell antigens or plasma cells, such as CD19 and BCMA, can eliminate autoantibody-producing B cells; thus, exhibit strong therapeutic effects in both preclinical^1085–1087^ and particularly clinical autoimmune conditions.^1088–1090^ Several CAR-T products targeting CD19 or BCMA or these two simultaneously are under early-phase clinical studies (Table 5). However, pan-B-cell depletion has side effect of lacking immunoglobulins.^1082^ To specifically target autoimmune B cells, CAAR-T cells which express autoantigens instead of traditional scFv have been developed. Hence, autoantigen recognition by autoreactive B cells leads to specific killing of pathogenic B cells by CAR-T cells.^1091^ A number of autoantigens have been identified highly associated with various types of autoimmune diseases.^1082^ CAAR-T cells expressing pemphigus vulgaris (PV) autoantigen desmoglein-3 (Dsg-3) and muscle specific kinase (MuSK) have been tested in phase I clinical trials for patients with mucosal-dominant PV and MuSK-myasthenia gravis, respectively^1092,1093^ (Table 5). Similarly, CAR-T cells expressing the ectodomains of pMHC complexes can specifically interact and eliminate pathogenic T cells.^1094^ For instance, CAR-T cells expressing I-A^g7^-B:9-23 (R3) complex that the insulin B-chain peptide B:9-23 is presented by MHC II, directly target pathogenic B:9-23–specific CD4^+^ cells and significantly delay the onset of diabetes.^1095^ Likewise, genetically engineered CAR-T cells with insulin B chain peptide fused with MHC I component β2 microglobulin (β2m) could reduce the pathogenic CD8^+^ T cells and ameliorate diabetes in NOD mice.^1096^

### CAR-T_reg_ cell therapy

Given the potent immunosuppressive activity of T_reg_ cells, therapeutic strategies harnessing T_reg_ cell function have been proposed to restore immune tolerance in autoimmune diseases. Low-dose IL-2 therapy and engineered IL-2 with different selectivity to IL-2R (IL-2 muteins) which can preferentially induce T_reg_ cell expansion and function without activating autoreactive Teff cells have demonstrated clinical efficacy in various autoimmune diseases.^1097,1098^ However, due to lacking of specificity, polyclonal T_reg_ cells have compromised suppressive activity, whereas CAR-T_reg_ cells with engineered CAR modules directing against autoantigens display stronger suppression of effector function.^1099^ CAR-T_reg_ cells have been extensively studied in preclinical models by targeting different autoimmune antigens, including MOG for EAE,^1100^ 2,4,6-trinitrophenyl (TNP),^1101^ and CEA^1102^ for colitis, citrullinated vimentin (CV) for RA,^1103^ as well as insulin for T1D.^1104^ In organ transplantation, HLA-A2 is commonly mismatched. CAR-T_reg_ cells designed to express HLA-A*02 CAR have been shown to induce immunosuppression of allograft-specific effector T cells and prevent graft-versus-host disease (GVHD) in preclinical models.^1105,1106^ Therefore, two phase I/II clinical trials of HLA-A2-CAR-T_reg_ cells (TX200-TR101 and QEL-001) have been registered for organ transplantation (Table 5).

## Conclusions

T cells are essential for functional immune responses. In this review, we summarize the current understandings of T cell development, CD4^+^ and CD8^+^ αβ T cell and γδ T cell subsets, fate decision and regulation, functional roles in pathophysiological conditions, especially in infectious diseases, chronic infection and tumors and autoimmune diseases as well as immunotherapies harnessing T cell function in preclinical and clinical development. Cytotoxic T cells, including both CD8^+^ and CD4^+^ CTLs, can directly eliminate infected or malignant cells, while CD4^+^ T helper cells mainly regulate/help both innate and adaptive immune responses through costimulation and cytokine signals. Major effector T cells, including different CD4^+^ Th cells, effector γδ T cells and CD8^+^ T_E_ cells are summarized regarding to their cellular and molecular characteristics (Table 6). Appropriate T cell immunity is essential for maintaining host homeostasis and preventing infections and malignancy, whereas aberrant T cell immune responses elicit and promote pathogenesis, tumor growth and autoimmune disorders, which may also affect its application in immunotherapy, such as CAR-T cell-induced CRS.^1107^

**Table 6:** Effector T cell subsets and key features

Effector T cells	Effector molecules	Surface markers	Differentiation induction	Master TF	Other regulatory TF	Functions	Refs
Th1	IFN-γ, TNF-α/β, IL-2	CXCR3, CCR5	IL-12, IFN-γ	T-bet	STAT1, STAT4	Defense intracellular pathogens; Cell-based immunity; Pro-inflammation	^76,78,80–82,950^
Th2	IL-2, IL-4, IL-5, IL-10, IL-13	CCR3, CCR4	IL-4	GATA-3	STAT6, NFAT1, c-Maf, IRF4, JunB, TCF-1	Defense extracellular pathogens; Humoral immunity; Tissue repair; Allergy	^81,84,86,241^
Th9	IL-9, IL-10, IL-21	IL-4R, TGFβR, IL-2R, OX40, GITR, Notch, DR3, TSLPR	IL-4, TGF-β	IRF4, PU.1	GATA-3, SMAD	Infectious diseases; Allergy; Cancer; Autoimmunity	^91–94,99,100^
Th17	IL-17A-F, IL-21, IL-10, IL-23, IL-22, IFN-γ, GM-CSF	IL-6R, TGFβR, IL-21R, IL-23R	IL-6, TGF-β, IL-21, IL-23	RORγt	RORα, c-Maf, p65, NFAT, c-Rel	Defense extracellular pathogens (fungi); Mucosal immunity; Autoimmunity	^102,104,108,111,117,252^
Tfh	IL-4, IL-21	PD-1, CXCR5, CD40, CD40LG, ICOS, SAP	IL-6, IL-21	Bcl-6	BATF, STAT1/3/4/5, Foxp1, KLF2, IRF4, Ets1, BACH2, Ascl2, Tox2, Bhlhe40, STAT5 and Blimp-1 (Inhibition)	Humoral immunity; Autoimmunity	^130–132,136,140,145,150^
Treg	IL-10, TGF-β, IL-35	CD25	TGF-β, IL-2	Foxp3	c-Rel, AP-1, NFAT, Smad2, Smad3, FoxO1, FoxO3, STAT5	Immunosuppression; Autoimmunity; Cancer	^151,152,154,156^
CD4 CTL	pro-inflammatory cytokines, perforin, granzymes, granulysin	KLRG1, NKG2A, NKG2D, CRTAM, Fas, TRAIL	IL-2, IL-12, IL-6, IFN-α	RUNX3	T-bet, Eomes, ThPOK (Inhibition)	Infectious diseases; Longevity; Cancer	^77,256,258,268,275,279^
Tγδ1	IFN-γ	CD27, CD122, NK1.1, CD45RB^hi^	Skint-1	T-bet	TCF-1, Lef1, Eomes, Id3	Tissue physiology; Defense pathogenic infections; (Anti-)Cancer	^938,943,947^
Tγδ17	IL-17A	CCR6, SCART2, CD45RB^lo^	IL-6, TGF-β, IL-1β, IL-18, IL-23	RORγt	c-Maf, Sox4, Sox13, HEB, Blk, RelB	Tissue physiology and pathophysiology; Defense pathogenic infections; (Pro-)Cancer	^938,943,944,948,952^
CD8 T_E_	IL-2, IFN-γ, TNF, perforin, granzymes, CCL5, CCL3	FasL, KLRG1, CX3CR1, CXCR6, CCR5	IL-2, IL-12, IL-21	T-bet	Blimp-1, Id2, STAT4, Zeb2	Viral infection; Cancer	^169,173,182,193,219,223^

T cell immunity is extremely critical but complex with significant cell heterogeneity, differentiation plasticity, functional diversity and exquisite regulatory mechanisms, which also display context-dependent features. For instance, upon acute infection, both CD4^+^ and CD8^+^ T cells differentiate into effector CD8^+^ T cells with robust expansion and cytotoxic functions, whereas those in chronic infection develop into exhaustion state with progressive loss of effector function and elevated inhibitory phenotype. The discrepancy of either tumor-promoting or tumor-protective effects of Th2, Th17, Th9, T_reg_, and Tγδ17 cells is mainly attributed to different tumor types. The differentiation plasticity of Th17 cells in tumor and autoimmune diseases is also highly dependent on the microenvironmental niche. The heterogeneity, plasticity and instability of T_reg_ cells, such as Th-like T_reg_ and exFoxp3 T_reg_ cells, play important and contradictory roles in autoimmune diseases. The diverse T cell differentiation and function depend on distinct but intersected molecular regulations at transcriptional, epigenetic and metabolic levels.

Despite a comprehensive elaboration on multiple aspects of T cells, some limitations in this review are: (1) classic αβ T and γδ T cells are mainly focused here, while rare T cell populations such as mucosal-associated invariant T (MAIT) cells and NKT cells also play essential roles in immune responses. (2) Most of the current understandings on T cell immunity are derived from mouse studies, albeit highly evolutionary conservation between mouse and human, T cell response in human subjects is more clinically relevant. (3) Universal features of T cells signature and function in each disease setting are summarized. However, context-specific T cells are present in response to discrete types of pathogens or cancers. (4) We mainly summarized T cell immunity at the cellular level regarding to cell development, differentiation and functionality, whereas the molecular signaling pathways are important to understand the underlying mechanisms. For instance, TCR signaling pathway is critical for T cells in almost every aspect and contributes to human health and disease, which has been comprehensively reviewed recently.^1108^ Collectively, given the importance and complexity of T cell immunity, both comprehensive and delicate research are required to fully reveal T cell signature and function. Especially with the advances in single-cell technologies, future investigations need to focus on characterizing new T cell subsets, context-specific T cell heterogeneity, functional states, differential plasticity, dysfunction and programmability to provide insights into novel therapeutic strategies in human diseases.
